# Recent Advances in Search of Bioactive Secondary Metabolites from Fungi Triggered by Chemical Epigenetic Modifiers

**DOI:** 10.3390/jof9020172

**Published:** 2023-01-28

**Authors:** Mengyao Xue, Xuwen Hou, Jiajin Fu, Jiayin Zhang, Jiacheng Wang, Zhitong Zhao, Dan Xu, Daowan Lai, Ligang Zhou

**Affiliations:** State Key Laboratory of Agrobiotechnology, Department of Plant Pathology, College of Plant Protection, China Agricultural University, Beijing 100193, China

**Keywords:** fungal biosynthetic gene cluster, cryptic secondary metabolite, chemical epigenetic modification, biosynthetic regulation, DNA methyltransferase, histone deacetylase, biological activities

## Abstract

Genomic analysis has demonstrated that many fungi possess essential gene clusters for the production of previously unobserved secondary metabolites; however, these genes are normally reduced or silenced under most conditions. These cryptic biosynthetic gene clusters have become treasures of new bioactive secondary metabolites. The induction of these biosynthetic gene clusters under stress or special conditions can improve the titers of known compounds or the production of novel compounds. Among the inducing strategies, chemical-epigenetic regulation is considered a powerful approach, and it uses small-molecule epigenetic modifiers, which mainly act as the inhibitors of DNA methyltransferase, histone deacetylase, and histone acetyltransferase, to promote changes in the structure of DNA, histones, and proteasomes and to further activate cryptic biosynthetic gene clusters for the production of a wide variety of bioactive secondary metabolites. These epigenetic modifiers mainly include 5-azacytidine, suberoylanilide hydroxamic acid, suberoyl bishydroxamic acid, sodium butyrate, and nicotinamide. This review gives an overview on the method of chemical epigenetic modifiers to trigger silent or low-expressed biosynthetic pathways to yield bioactive natural products through external cues of fungi, mainly based on the research progress in the period from 2007 to 2022. The production of about 540 fungal secondary metabolites was found to be induced or enhanced by chemical epigenetic modifiers. Some of them exhibited significant biological activities such as cytotoxic, antimicrobial, anti-inflammatory, and antioxidant activity.

## 1. Introduction

The discovery of novel natural compounds with diverse structures and biological activities is an important aspect in new drug research and development [1,2]. Fungal secondary metabolites are highly complex and have a rich diversity that makes fungi a treasure of bioactive secondary metabolites. Traditional methods used to discover bioactive natural products from fungi usually include sample collection, the cultivation of fungal strains, extraction, bioassay-guided isolation, structural elucidation, and bioactivity evaluation. The genomic analyses of fungi have shown that a large number of gene clusters controlling the expression of secondary metabolites are usually kept in silent status under traditional laboratory culture conditions [3,4]. It is urgent to activate the expression of these silenced genes to obtain more secondary metabolites with novel structures and remarkable biological activities [5]. Focusing on silencing gene activation, a variety of successful strategies have been achieved such as the one strain many compounds (OSMAC) method by changing cultivation parameters (i.e., carbon source, nitrogen source, light intensity, ambient pH, shaking, aeration, incubation temperature, redox status, and metal ions), global regulation, epigenetic manipulation, and genome mining strategies [6,7,8,9,10,11]. Among these, chemical epigenetic manipulation has been demonstrated to be an effective method for enhancing secondary metabolite expression without altering genes or causing the hereditable manipulation of organisms [12]. Notably, epigenetic modification was proven to be effective to the host to trigger the latent biosynthetic pathways to yield cryptic natural products [13].

Molecular and chemical epigenetic modifications are two aspects in search of secondary metabolites from fungi. The molecular epigenetic modification method is mainly through the knockout or overexpression of coding genes of epigenetic related enzymes, while chemical epigenetic modification method is the exogenous addition of chemical epigenetic modification enzyme inhibitors such as DNA methyltransferase (DNMT) inhibitors, histone deacetylase (HDAC) inhibitors, and histone acetyltransferase (HAT) inhibitors. These inhibitors can promote gene transcription, then activate silent biosynthetic gene clusters, and improve the chemical diversity of secondary metabolites of fungi [14,15].

In the past decade, biosyntheses of diverse compounds were successfully activated by treating fungi with epigenetic modifiers. Some reviews were published about natural fungal product development under chemical epigenetic modulation [16,17,18,19]. In this mini-review, we focused on the production of secondary metabolites by using chemical epigenetic modifiers according to the epigenetic-related enzymes to summarize the effects of these chemical modifiers on the biosynthesis of secondary metabolites in fungi.

## 2. Chemical Epigenetic Modifiers and Their Action Mechanisms

**Figure 1:**
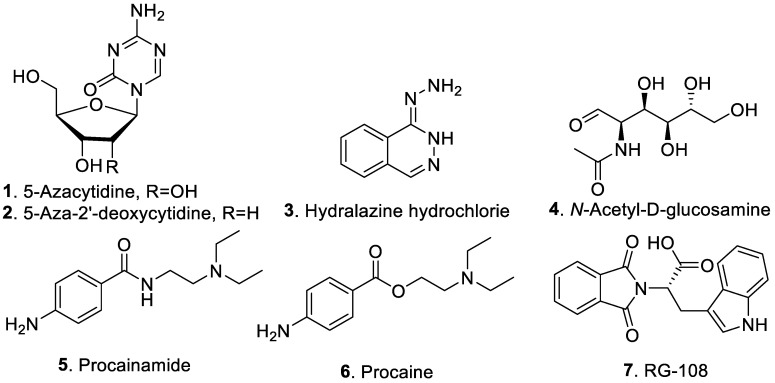

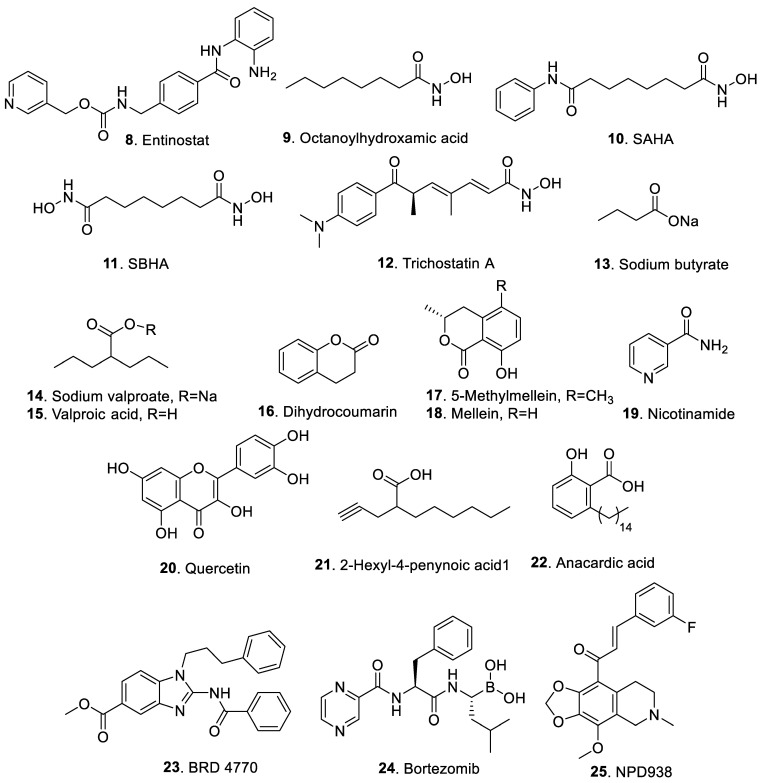
Structures of the epigenetic small organic chemicals **1**–**25** that affect secondary metabolism in fungi.

Chemical epigenetic modifiers are natural or synthetic small molecular compounds that target epigenetic enzymes, leading to epigenetic alterations of the organisms [17,20,21]. The structures of commonly used chemical epigenetic modifiers for generating specialized metabolism in fungi are shown in Figure 1 with their names and action mechanisms listed in Table 1. Many of these compounds act by inhibiting enzyme machinery essential for transferring methyl, acetyl, and alkyl groups to DNA or histones. The target sites of DNMT, HDAC, and proteasome inhibitors are DNA, heterochromatin, and proteasome, respectively [18,22,23].

### 2.1. DNA Methyltransferase Modifiers

DNA methyltransferases (DNMTs) are a conserved family of cytosine 5′-carbon atom methylases that play an essential role in maintaining DNA methylation patterns, transcriptional activation, and silencing [24]. Inhibition by DNMT inhibitors results in passive demethylation through consecutive DNA replication cycles [17]. DNMT inhibitors include 5-azacytidine (5-Aza, **1**), 5-aza-2′-deoxycytidine (decitabine, **2**), hydralazine hydrochloride (**3**), *N*-acetyl-D-glucosamine (GlcNAc, **4**), procainamide (**5**), procaine (**6**), and *N*-phthalyl-L-tryptophan (RG-108, **7**) (Table 1).

The typical DNMT inhibitor is 5-Aza (**1**), which is a derivative of the nucleoside cytidine; 5-Aza (**1**) can be incorporated into DNA and less into RNA, resulting in the trapping and inactivation of DNMT. Additionally, 5-Aza (**1**) rapidly depletes cellular DNMT and reduces the methylation level of the genomic DNA [25]. Therefore, 5-Aza (**1**) is widely used as a potent DNMT inhibitor in the field of epigenetics [24], including fungal secondary metabolism [26].

### 2.2. Histone Deacetylase Modifiers

Histone deacetylases (HDACs) are a group of enzymes that remove the acetyl group from the lysine residue(s) of histones and non-histone proteins and thereby regulate the gene transcription level. Transcriptional regulation in eukaryotes occurs within a chromatin setting and is strongly influenced by the post-translational modification of histones. HDACs act as transcription repressors and consequently promote chromatin condensation in cells.

HDACs are divided into three classes, namely classes I, II, and III [27]. HDAC inhibitors alter gene expression patterns and endorse changes in nonhistone proteins occurring at the post-translational level [26,28]. They are structurally classified into four groups, including hydroxamates, cyclic peptides, aliphatic acids, and benzamides [29]. HDAC inhibitors applied in fungal secondary metabolisms mainly include entinostat (MS-275, **8**), octanoylhydroxamic acid (OHA, **9**), suberoylanilide hydroxamic acid (vorinostat, SAHA, **10**), suberoylbishydroxamic acid (SBHA, **11**), trichostatin A (TSA, **12**), sodium butyrate (NaBut, **13**), sodium valproate (SVP, **14**), valproic acid (VPA, **15**), dihydrocoumarin (**16**), 5-methylmellein (**17**), mellein (**18**), nicotinamide (**19**), quercetin (**20**), and 2-hexyl-4-pentynoic acid (HPTA) (**21**) (Table 1).

SAHA (**10**), TSA (**12**), and sodium butyrate (**13**) are frequently used as HDAC inhibitors in fungi. Both SAHA (**10**) and TSA (**12**) present a hydroxamic group that binds to the zinc ion of class I and II HDAC inhibitors, thus preventing HDAC activities. Sodium butyrate (**13**) inhibits the histone deacetylase activity, leading to differentiation in eukaryotic cells [17]. TSA (**12**) and other HDAC inhibitors such as SAHA (**10**), sodium butyrate (**13**), and valproic acid (**15**) have been shown to enhance the chemical diversity of secondary metabolites produced by fungi from the genera *Clonostachys*, *Diatrype*, and *Verticillium* [26]. Valproic acid (**15**) is frequently used to inhibit class I HDACs and also induces the proteosomal degradation of class II HDACs [30].

### 2.3. Other Chemical Epigenetic Modifiers

Other chemical epigenetic modifiers applied in fungal secondary metabolisms include histone acetyltransferase inhibitors (i.e., anacardic acid, **22**) [31], histone methytransferase inhibitors (i.e., BRD4770, **23**) [32], and proteasome inhibitors (i.e., bortezomib, **24**) [33]. NPD938 (**25**) is an epigenetic modifier with an unclear action mechanism that is considered to enhance the production of fungal secondary metabolites via the global regulator LAE1 [34].

**Table 1:** Commonly used chemical epigenetic modifiers for generating specialized metabolism in fungi.

Modifier	Mechanism of Action	Ref.
5-Azacytidine (**1**)	Inhibition of DNA methyltransferase	[26,35]
5-Aza-2′-deoxycytidine (**2**)	Inhibition of DNA methyltransferase	[36]
Hydralazine hydrochloride (**3**)	Inhibition of DNA methyltransferase	[13]
*N*-Acetyl-D-glucosamine (**4**)	Inhibition of DNA methyltransferase	[25]
Procainamide (**5**)	Inhibition of DNA methyltransferase	[32]
Procaine (**6**)	Inhibition of DNA methyltransferase	[37]
*N*-Phthalyl-L-tryptophan (**7**)	Inhibition of DNA methyltransferase	[38]
Entinostat (**8**)	Inhibition of HDAC of class I	[39]
Octanoylhydroxamic acid (**9**)	Inhibition of HDAC of classes I and II	[40]
Suberoylanilide hydroxamic acid (**10**)	Inhibition of HDAC of classes I and II	[41,42]
Suberoylbishydroxamic acid (**11**)	Inhibition of HDAC of classes I and II	[42]
Trichostatin A (**12**)	Inhibition of HDAC of classes I and II	[43]
Sodium butyrate (**13**)	Inhibition of HDAC of classes I and II	[25]
Sodium valproate (**14**)	Inhibition of HDAC of classes I and II	[44]
Valproic acid (**15**)	Inhibition of HDAC of classes I and II	[44]
Dihydrocoumarin (**16**)	Inhibition of NAD^+^-dependent HDAC of class III	[45]
5-Methylmellein (**17**)	Inhibition of NAD^+^-dependent HDAC of class III	[46]
Mellein (**18**)	Inhibition of NAD^+^-dependent HDAC of class III	[46]
Nicotinamide (**19**)	Inhibition of NAD^+^-dependent HDAC of class III	[47]
Quercetin (**20**)	Inhibition of NAD^+^-dependent HDAC of class III; inhibition of protein kinases; inhibition of DNA topoisomerases; regulation of gene expression	[18]
2-Hexyl-4-pentynoic acid (**21**)	Inhibition of histone deacetylase	[48]
Anacardic acid (**22**)	Inhibition of histone acetyltransferase	[31]
BRD4770 (**23**)	Inhibition of histone methytransferase	[32]
Bortezomib (**24**)	Inhibition of proteasome	[33]
NPD938 (**25**)	The action mechanism was not clear	[34,49]

## 3. Effects of DNA Methyltransferase Modifiers

The chemical modifiers of DNA methyltransferase (DNMT), which include 5-azacytidine, 5-aza-2′-deoxycytidine, and procaine, have been reported to display effects on the production of fungal secondary metabolites.

### 3.1. Effects of 5-Azacytidine

One of the main DNA methyltransferase modifiers is 5-Azacytidine (5-Aza), which has been used in the chemical epigenetic regulation of fungal secondary metabolism [16,18,26,50]. The addition of 5-Aza in the media could activate or inhibit the production of secondary metabolites of many fungi. Some examples of 5-Aza affecting the production of fungal secondary metabolites are listed in Table S1. The structures of compounds **26**–**132** isolated from fungi treated with 5-Aza are shown in Figure S1.

The supplementation of 5-Aza in the culture medium of the endophytic fungus *Alternaria* sp. at 250 μM induced the production of mycotoxins, including alternariol (**26**), alternariol-5-*O*-methyl ether (**27**), 3′-hydroxyalternariol-5-*O*-methyl ether (**28**), altenusin (**29**), tenuazonic acid (**30**), and altertoxin II (**31**) [51].

The cultures of the gorgonian-derived fungus *Aspergillus* sp. XS-20090066 were treated with 5-Aza at 100 μM in rice medium. The production of six bisabolane-type sesquiterpenoids, including (7*R*)-hydroxysydonic acid (**32**), (7*S*)-sydonic acid (**33**), (*S*)-5-(hydroxymethyl)-2-(2′,6′,6′-trimethyltetrahydro-2*H*-pyran-2-yl)phenol (**34**), (7*S*,11*S*)-12-hydroxysydonic acid (**35**), (7*S*)-11-dehydrosydonic acid (**36**), and (*S*)-sydowic acid (**37**), was activated. Compounds (7*R*)-hydroxysydonic acid (**32**), (7*S*)-sydonic acid (**33**), and (*S*)-5-(hydroxymethyl)-2-(2′,6′,6′-trimethyltetrahydro-2*H*-pyran-2-yl)phenol (**34**) showed a broad spectrum of activities against five tested bacteria, *Staphylococcus aureus*, *Bacillus cereus*, *Kocuria rhizophila*, *Pseudomonas putida*, and *P. aeruginosa*, with MIC ≤ 25 μM. In particular, (*S*)-5-(hydroxymethyl)-2-(2′,6′,6′-trimethyltetrahydro-2*H*-pyran-2-yl)phenol (**34**) showed pronounced antibacterial activity against *S. aureus* with an MIC value of 3.13 μM, which was close to the positive control ciprofloxacin (MIC, 2.5 μM) [52].

When 5-Aza was added to the medium of *Aspergillus clavatus* at 2 μM, the production of cytochalasin E (**38**), patulin (**39**), and pseurotin A (**40**) significantly increased [25].

Aflatoxins are a group of potent mycotoxins with carcinogenic, hepatotoxic, and immunosuppressive properties, and they are mainly produced by *Aspergillus flavus* and *A. parasiticus*. *A. flavus* is a common saprophyte and opportunistic pathogen for producing aflatoxins and many other secondary metabolites. 5-Aza was found to inhibit aflatoxin B1 (**41**) biosynthesis of *A. flavus* at 1 mM [53,54,55]. 5-Aza also inhibited aflatoxin B1 (**41**) biosynthesis of *A. parasiticus* at 1 mM [56].

The production of both (*Z*)-9-octadecenoic acid (**42**) and 12-methyl-tetradecanoic acid methylester (**43**) was stimulated by the addition of 5-Aza at 1 μM to the cultures of endophytic fungus *A. niger* isolated from the roots of *Terminalia catappa* [15].

The addition of 5-Aza at 100 μM to the culture broth of *A. sydowii* changed its profile of secondary metabolites. The analysis of the extract of culture broth led to the isolation of three new bisabolane-type sesquiterpenoids, namely (7*S*)-sydonic acid (**33**), (7*S*)-7-*O*-methylsydonol (**44**), (7*S*,11*S*)-12-hydroxysydonic acid (**45**), and 7-deoxy-7,14-didehydrosydonol (**46**), along with eight known compounds including (*S*)-sydonol (**47**), anhydrowaraterpol B (**48**), (*E*)-5-(hydroxymethyl)-2-(6′-methylhept-2′-en-2′-yl)phenol (**49**), AGI-B4 (**50**), sydowinin A (**51**), sydowinin B (**52**), and diorcinol (**53**). The isolated compounds were evaluated for their anti-diabetic and anti-inflammatory activities. Among them, (*S*)-sydonol (**47**) not only increased insulin-stimulated glucose consumption but also prevented lipid accumulation in 3T3-L1 adipocytes. Additionally, (*S*)-sydonol (**47**) exhibited significant anti-inflammatory activity through inhibiting superoxide anion generation and elastase release by fMLP/CB-induced human neutrophils [57].

The addition of 5-Aza at 50 μM in rice medium of *A. terreus* GZU-31-1 led to the discovery of five butanolide derivatives, namely asperbutyrolactones A (**54**) and B (**55**), aspulvinone E (**56**), butyrolactone I (**57**), and butyrolactone VI (**58**), and four known diphenyl ether derivatives, namely asterric acid (**59**), penicillither (**60**), methyl asterrate (**61**), and 4′,6′-dichloroasterric acid (**62**). Of these, asperbutyrolactones A (**54**) and B (**55**) were previously undescribed compounds. The isolated metabolites were tested for their anti-inflammatory effects on the production of nitric oxide in lipopolysaccharide-induced microglial cells (RAW 264.7 cells). Asperbutyrolactone A (**54**), penicillither (**60**), methyl asterrate (**61**), and 4′,6′-dichloroasterric acid (**62**) exhibited more potent anti-inflammatory activity with IC_50_ values of 16.31, 20.16, 9.53, and 21.64 μM, respectively, than the positive control (indomethacin, IC_50_, 24.0 μM) [58].

Two endophytic fungi *Botryosphaeria rhodina* and *Phomopsis* sp. MD 86 from *Nothapodytes nimmoniana* (Icacinaceae) were screened to produce camptothecine (CPT, **63**). The production of CPT (**63**) was greatly increased when two endophytic fungi were treated with 10 mg/L of 5-Aza, respectively [59].

Treatment of *Chaetomium* sp. with 5-Aza at 6 mM resulted in an enhanced accumulation of isosulochrin (**64**) [60].

Four novel compounds, including two cyclopentenones, globosporins A (**65**) and B (**66**), and two monoterpenoid indole alkaloids, globosporines C (**67**) and D (**68**), as well as three known compounds, pseurotin A (**40**), mappianine E (**69**), and 19(20)*Z*-5-carboxymethylvallesiachotamine (**70**), were isolated from the endophytic fungus *Chaetomium globosporum* from *Euphorbia humifusa* by exposure to 5-Aza at 120 mg/L. Two indole alkaloids, globosporines C (**67**) and D (**68**), showed antimicrobial activities against three phytopathogenic bacteria, *Xanthomonas oryzae* pv. *oryzae*, *X. oryzae* pv. *oryzicola*, and *Pseudomonas syringae* pv. *lachrymans* with MIC values in the range of 14–72 μg/mL. Mostly, globosporine D (**68**) was proven to have potent anti-phytopathogenic activity against *X. oryzae* pv. *oryzae* in vitro and in vivo, which suggests that globosporine D (**68**) has the potential to be developed as a bactericidal candidate for the prevention of rice bacterial leaf blight disease [61].

The treatment of *Cladosporium cladosporioides* with 5-Aza at 0.1 μM–10 mM elicited the de novo production of several oxylipins, which were identified as (9*Z*,12*Z*)-11-hydroxyoctadeca-9,12-dienoic acid (**71**), its methyl ester (**72**), and glycerol conjugate (**73**), in substantial yields [26].

The cultivation of *Cochliobolus lunatus* TA26–46 with 5-Aza at 10 μM led to the isolation of seven new diethylene glycol phthalate esters, cochphthesters A–G (**74**–**80**), along with four known analogues: 1,2-benzenedicarboxylic acid,1,2-bis [2-(2-hydroxyethoxy) ethyl] ester (**81**), 1,2-benzenedicarboxylic acid, 1,1-(oxydi-2,1-ethanediyl) 2,2-bis [2-(2-hydroxyethoxy) ethyl] ester (**82**), 1,2-benzenedicarboxylic acid, 1,2-bis [2-[[2-[[2-(2-hydroxyethoxy) ethoxy] carbonyl] oxy] ethoxy] ethyl] ester (**83**), and 1,2-benzenedicarboxylic acid, oxydi-2,1-ethanediyl dimethyl ester (**84**) [62].

The cultivation of *Cochliobolus lunatus* TA26-46 with 5-Aza at 10 µM in Czapek-Dox liquid medium led to the isolation of induced compounds, including two α-pyrones, namely cochliobopyrones A (**85**) and B (**86**), three isocoumarins, namely 3-methyl-6,8-dihydroxyisocoumarin (**87**), 6-hydroxy-8-methoxy-3-methylisocoumarin (**88**), and (*S*)-orthosporin (**89**), and one chromone, altechromone A (**90**) [63].

The treatment of *Cophinforma mamane* with 5-Aza at 1 μM significantly reduced the production of thiodiketopiperazines (TDKPs) botryosulfuranols A (**91**), B (**92**), and C (**93**) [64].

Treatment of the culture broth of *Cordyceps indigotica* with 5-Aza at 100 μM led to the production of aromatic polyketide glycosides indigotides A (**94**) and B (**95**) [65].

Endophytic fungus *Diaporthe perseae* from *Gloriosa superba* tubers was treated with 5-Aza at 1 μM. The production of colchicine (**96**) was found to be increased [66].

Two new glycosylated polyketides, namely lunalides A (**97**) and B (**98**), were produced in the cultures of *Diatrype* sp. treated with 0.1–10 μM of 5-Aza [26].

*Dimorphosporicola tragani* CF-090383 was found to produce three dendrodolide mycotoxins, namely dendrodolides E (**99**), G (**100**), and I (**101**), when 5-Aza was added to the fermentations at 100 μM [67].

*Lophiotrema* sp. F6932 was fermented in CF02LB medium supplemented with 5-Aza at 50 μM, which led to the identification of two spirobisnaphthalenes, palmarumycins CP30 (**102**) and C8 (**103**). Palmarumycin C8 (**103**) showed inhibitory activity against *Staphylococcus aureus* with an IC_90_ value of 18 μg/mL and also possessed antiproliferative activities with IC_50_ values of 1.1 μg/mL and 2.1 μg/mL against MIA PaCa-2 and PANC-1 cell lines, respectively [68].

When the cultures of *Muscodor yucatanensis* Ni30 were treated with 5-Aza at 50 μM, the growth rate, mycelia morphology, and pigmentation were defected, and the production of VOCs, ergosterol (**104**), and xylaguaianol C (**105**) was enhanced [69].

Two new polyketides modified with a rare methylsulfonyl group, 3-methoxy-6-methyl-5-(methylsulfonyl)benzene-1,2,4-triol (**106**) and neosartoryone A (**107**), were isolated from *Neosartorya udagawae* HDN13-313 cultivated with 5-Aza at 73 mg/L. Their methylsulfonyl group was proven to be derived from DMSO, which was used as the solvent to dissolve 5-Aza. Neosartoryone A (**107**) showed lipid-lowering activity in vitro comparable to the positive control simvastatin [70].

The guttates collected from cultures of *Penicillium citreonigrum* treated with 50 µM of 5-Aza were highly enriched in secondary metabolites, including sclerotiorin (**108**), ochrephilone (**109**), dechloroisochromophilone III (**110**), dechloroisochromophilone IV (**111**), 6-((3*E*,5*E*)-5,7-dimethyl-2-methylenenona-3,5-dienyl)-2,4-dihydroxy-3-methylbenzaldehyde (**112**), sclerotioramine (**113**), pencolide (**114**), atlantinones A (**115**), and B (**116**). While pencolide (**114**) was detected in the exudates of both the control and 5-Aza-treated cultures, all of the other metabolites were found exclusively in the guttates of the 5-Aza-modified fungus. Both sclerotiorin (**108**) and sclerotioramine (**113**) caused modest inhibition on *Staphylococcus epidermidis*. Only sclerotioramine (**113**) was active against the growth of *Candida* strains [35].

The cultivation of *Penicillium minioluteum* with 5-Aza at 500 μM led to the isolation of a novel type of aspertetronin dimers, named miniolins A–C (**117**–**119**), along with their precursor aspertetronin A (**120**). The miniolins showed moderate cytotoxic activity against HeLa cell lines [71].

*Penicillium variabile* HXQ-H-1A was isolated from the mangrove rhizosphere soil collected from Fujian, China. An addition of 5-Aza at 0.2 mM in the medium led to production of a highly modified fatty acid amide, varitatin A (**121**). It displayed significant cytotoxicity against HCT-116 cells with an IC_50_ value of 2.8 μM. Moreover, it exhibited 50% and 40% inhibitory activity against tyrosine kinases PDGFR-β and ErbB4 at a concentration of 1 μM, respectively [72].

An addition of 5-Aza at 500 mM to the culture medium of the plant endophytic fungus, *Pestalotiopsis crassiuscula*, obtained from the leaves of *Fragaria chiloensis*, dramatically induced the production of 4,6-dihydroxy-3,7-dimethylcoumarin (**122**), pestalotiopyrone G (**123**), and 2′-hydroxy-6′-hydroxy-methyl-4′-methylphenyl 2,6-dihydroxy-3-(2-isopentenyl)benzoate (**124**) [73].

Analysis of the culture broth extract of the endophytic fungus *Pestalotiopsis microspora* treated with 5-Aza at 500 μM led to the isolation of a new compound, 4′-formamidophenyl-5-methoxybenzoate (**125**), along with seven known polyketides, pestalotiopyrone G (**123**), 4-hydroxybenzoic acid (**126**), LL-P880α (**127**), 2′-hydroxy 6′-hydroxymethyl-4′-methylphenyl-2,6-dihydroxy-3-(2-isopentenyl)benzoate (**128**), pestalotiollide B (**129**), endocrocin (**130**), and 1′-hydroxy-4-methoxy-6-pentyl-2*H*-pyran-2-one (**131**). The compounds, except for 1′-hydroxy-4-methoxy-6-pentyl-2*H*-pyran-2-one (**131**), belonged to the newly induced secondary metabolites [74].

It was found that resveratrol (**132**) was enhanced in case of treatment with 5-Aza at 10 μM to yield 48.94 μg/mL in the culture of *Xylaria psidii*, which was an endophytic fungus isolated from the leaves of *Vitis vinifera* [75].

Some fungal species such as *Cladosporium reesinae*, *Hypoxylon* sp., and *Neurospora crassa* were also treated with 5-Aza, and the production of secondary metabolites was induced. Unfortunately, the increased metabolites have not been structurally identified. Treatment of 5-Aza at 10 μM to the cultures of *Cladosporium reesinae* NRL-6437 increased the production of unidentified antimicrobial metabolites [76]. The cultures of *Hypoxylon* sp. CI-4 were treated with 5-Aza at 100 μM to induce the production of VOCs (volatile organic compounds) which composed of terpenes, alkanes, alkenes, organic acids, and benzene derivatives [77]. 5-Aza was added in the cultures of *Neurospora crassa* at 30 μM, and it stimulated the light-induced carotenoid synthesis by 30%, whereas a higher concentration of 5-Aza was toxic to carotenoid synthesis and mycelial growth [78].

### 3.2. Effects of Other DNA Methyltransferase Modifiers

Except for 5-Aza, other DNA methyltransferase modifiers, including 5-aza-2′-deoxycytidine, *N*-acetyl-D-glucosamine (GlcNAc), and procaine, were also found to activate the production of fungal secondary metabolites. The structures of compounds **133**–**138** isolated from fungi treated with other DNA methyltransferase modifiers are shown in Figure S2.

Three new α-pyrone derivatives, namely (*S*)-6-(sec-butyl)-5-(hydroxymethyl)-4-methoxy-2*H*-pyran-2-one (**133**), (5*S*,7*R*)-7-ethyl-4,5-dimethoxy-7-methyl-5,7-dihydro-2*H*-furo [3,4-b]pyran-2-one (**134**), and (5*R*,7*R*)-7-ethyl-4,5-dimethoxy-7-methyl-5,7-dihydro-2*H*-furo [3,4-b]pyran-2-one (**135**), were induced by 5-aza-2′-deoxycytidine at 10 mg/L in fermentation culture of the endophytic fungus *Penicillium herquei*, which was obtained from the fruiting body of *Cordyceps sinensis* [79].

*N*-Acetyl-D-glucosamine (GlcNAc) is a chitin compound. For the cultures of *Aspergillus clavaus*, GlcNAc at 0.5 μM significantly increased the production of pseurotin A (**40**) compared to the control [25].

The addition of procaine at 1 μM in the cultures of marine-derived fungus *Aspergillus unguis* DLEP2008001 induced the production of three metabolites, namely aspergillusidone F (**136**), unguinol (**137**), and unguisin A (**138**) [37].

### 3.3. Effects of Combinational Treatment with Two DNA Methyltransferase Modifiers

Combinational treatment with two DNA methyltransferase modifiers can increase the production of secondary metabolites of fungi. The structures of compounds **139**–**149** isolated from fungi treated with two DNA methyltransferase modifiers are shown in Figure S3.

When *Aspergillus clavatus* was treated with the combination of *N*-acetyl-D-glucosamine (GlcNAc, 5 μM) with 5-Aza (0.5 μM), the production of pseurotin A (**40**) was significantly increased. It was possible that both GlcNAc and 5-Aza had synergistic effects on the production of pseurotin A, which should be further studied in detail (**40**) [25].

Four new polyketide derivatives, pestalotiopols A–D (**139**–**142**), together with seven known compounds, (*S*)-6-(hydroxymethyl)-4-methyl-5,6-dihydro-2*H*-pyran-2-one (**143**), heterocornols A (**144**), E (**145**), and F (**146**), pestalotiophol B (**147**), dendocarbin B (**148**), and 2α-hydroxyisodrimeninol (**149**), were isolated from the chemical-epigenetic cultures of *Pestalotiopsis* sp. containing 5-aza-2′-deoxycytidine (10 μM) and RG-108 (10 μM). Among these compounds, pestalotiopols A (**139**) and B (**140**) and heterocornols A (**144**) and E (**145**) exhibited cytotoxicity against four human cancer cell lines (i.e., carcinoma cell line BGC-823, hepatocellular carcinoma cell line SMMC-7721, carcinoma cell line Ichikawa, and kidney cancer cell line 7860), with IC_50_ values of 16.5–56.5 mM [80].

## 4. Effects of Histone Deacetylase Modifiers

Histone deacetylase modifiers that activate the production of fungal secondary metabolites include octanoylhydroxamic acid (OHA), trichostatin A (TSA), SAHA, SBHA, sodium valproate, and nicotinamide.

### 4.1. Effects of Suberoylanilide Hydroxamic Acid

Suberoylanilide hydroxamic acid (SAHA) is also called vorinostat. It is the most widely used histone deacetylase modifier for the induction of the secondary metabolite production of fungi [29]. Some examples of SAHA affecting the production of fungal secondary metabolites are listed in Table S2. The structures of compounds **150**–**290** isolated from fungi treated with SAHA are shown in Figure S4.

The production of two new 3-(4-oxopyrano)-chromen-2-ones, namely aspyranochromenones A (**150**) and B (**151**), along with nine known metabolites, namely 6,7-dihydroxymellein (**152**), terrein (**153**), (*3R*)-6-hydroxymellein (**154**), (*R*)-orthosporin (**155**), 6,7-dimethoxymellein (**156**), 6-methoxymellein (**157**), (2*E*,6*E*,10*E*)-12-hydroxyfarnesol (**158**), 5,6-dihydroxymellein (**159**), and ethyl 3-methylorsellinate (**160**), was induced when the cultures of the endophytic fungus *Aspergillus* sp. AST0006 were treated with 250 μM of SAHA [81].

When SAHA (100 μM) was added in the fermentation of *Aspergillus calidoustus* and *Aspergillus westerdijkiae*, respectively, the diketopiperazine alkaloid phenylahistin (**161**) in *A. calidoustus* and the polyketide penicillic acid (**162**) in *A. westerdijkiae* were found to be increased [82].

The fungus *Aspergillus nidulans* treated with SAHA at 100 μM obviously induced the production of fellutamides fellutamide B (**163**), antibiotic 1656G (**164**), and antibiotic 3127 (**165**) [83]. When *A. nidulans* was treated with SAHA at 100 μM, the production of other metabolites aspercryptins A1 (**166**) and A2 (**167**) was also induced [84].

Nygerone A (**168**) is a new fungal metabolite featuring a unique 1-phenylpyridin-4(1*H*)-one core. It was obtained from *Aspergillus niger* ATCC1015 treated with SAHA at 10 μM [41].

*Aspergillus terreus* PF26 from the marine sponge *Phakellia fusca* was treated with SAHA at 500 μM. The production of terrein (**153**) was then enhanced. The production of (3*R*)-6-hydroxymellein (**154**) as the precursor of terrein (**153**) was also promoted [85].

SAHA was applied in the cultures of the marine-derived fungus *Aspergillus terreus* RA2905 at a concentration of 100 μM. It was found that the metabolic profile was significantly changed. Four new compounds, including a pair of enantiomers, (+)- and (-)-asperfuranones (**169** and **170**), together with two benzyl pyrones, asperpyranones A (**171**) and B (**172**), were identified from its ethyl acetate extract. These four compounds displayed antifungal activities against *Candida albicans* with MIC values of 32, 16, 64, and 64 μg/mL and PTP1B inhibitory activities with the IC_50_ values of 45.79, 17.32, 35.50, and 42.32 μM, respectively. Asperpyranone A (**171**) exhibited antibacterial activity against *Pseudomonas aeruginosa* with a MIC value of 32 μg/mL [86].

Cultivation of the marine-derived *Aspergillus versicolor* MCCC 3A00080 with the addition of SAHA significantly enhanced the diversity of the secondary metabolites. From the cultures treated with SAHA at 20 mg/L, a new biphenyl derivative, named versiperol A (**173**), along with two known compounds, diorcinol (**53**) and 2,4-dimethoxyphenol (**174**), were isolated. Among the isolated compounds, versiperol A (**173**) exhibited modest inhibition on the bacterium *Staphylococcus aureus* growth with an MIC value of 8 μg/mL [87].

The marine algicolous fungus *Aspergillus versicolor* OUCMDZ-2738 was treated with SAHA at 10 μM. Eight metabolites, diorcinol (**53**), 3-[6-(2-methylpropyl)-2-oxo-1*H* pyrazin-3-yl] propanamide (**175**), brevianamide X (**176**), brevianamide R (**177**), brevianamide Q (**178**), diorcinol C (**179**), diorcinol E (**180**), and methyl diorcinol-4-carboxylate (**181**), were induced for production. Both diorcinol (**53**) and methyl diorcinol-4-carboxylate (**181**) showed selective antibacterial activities against *Pseudomonas aeruginosa**,*** with minimum inhibitory concentrations (MICs) of 17.4 µM and 13.9 µM, respectively [88].

Through the addition of SAHA at 20 μM in the cultures of the *Aspergillus wentii* strain (na-3) isolated from the tissue of the brown alga *Sargassum fusiforme*, two new aromatic norditerpenes, aspewentins A (**182**) and B (**183**), along with an oxygenated derivative, aspewentin C (**184**), were obtained [89].

The addition of SAHA at 100 μM in the cultures of *Aspergillus westerdijkiae* induced the production of polyketide penicillic acid (**162**) [82]. A broad spectrum of biological activities including antibacterial, antifungal, antiviral, antitumor, and herbicidal activity has been reported for penicillic acid (**162**). This illustrates the potential of epigenetic manipulation for improving the fermentation efficiency of penicillic acid (**162**) [90].

The addition of SAHA at 100 μM in cultures of *Asteromyces cruciatus* led to the induced production of primarolides A (**185**) and B (**186**) [91].

The addition of SAHA at 500 μM to the culture of the filamentous fungus *Beauveria felina* significantly changed its secondary metabolite profile and led to the isolation of eight cyclodepsipeptides, including desmethylisaridin E (**187**), isaridin E (**188**), desmethylisaridin C2 (**189**), isaridin C2 (**190**), isaridin F (**191**), destruxin A (**192**), reseotoxin B (**193**), and roseocardin (**194**). Among them, desmethylisaridin E (**187**) inhibited superoxide anion production, and desmethylisaridin C2 (**189**) inhibited elastase release, with IC_50_ values of 10.00 μM and 10.01 μM, respectively [92].

The addition of SAHA at 300 μM in the cultures of *Bjerkandera adusta* led to the induced production of six tremulane sesqiterpenoids, namely 11,12-dihydroxy-1-tremulen-5-one (**195**), (3*S*,6*R*,7*R*)-tremul-1-ene-6,11,12-triol (**196**), ceriponol A (**197**), conocenol B (**198**), and conocenolides A (**199**) and B (**200**) [93].

The chemical epigenetic manipulation of *Botrytis cinerea* strain B05.10 with SAHA at concentrations ranging from 50 to 200 μM led to the isolation of a new cryptic metabolite, botrycinereic acid (**201**). This compound was also overproduced by inactivating the *stc2* gene, which encodes an unknown sesquiterpene cyclase [10].

Treatment of *Chaetomium* sp. with SAHA at 6 mM resulted in an enhanced accumulation of isosulochrin (**64**) [60].

The addition of SAHA at 1 mM to the cultures of *Chalara* sp. 6661 resulted in the production of four new modified xanthones, which were aniline-modified chalanilines A (**202**) and B (**203**) and adenosine-coupled xanthones A (**204**) and B (**205**). The aniline moiety in chalanilines A (**202**) and B (**203**) was verified to be derived from SAHA (vorinostat) [94].

The cultures of *Cladosporium cladosporioides* were treated with SAHA at a concentration of 10 mM to produce a complex series of perylenequinones, two of which were characterized as new metabolites, cladochromes F (**206**) and G (**207**), along with five known cladochromes A (**208**), B (**209**), D (**210**), and E (**211**) and calphostin B (**212**) [26].

When the cultures of *Cladosporium reesinae* NRL-6437 were treated with SAHA at 10 μM, the production of antimicrobial metabolites was activated. However, their structures have not been characterized [76].

The treatment of SAHA at 300 μM on the cultures of *Cladosporium sphaerospermum* L3P3 led to the induced production of cladosins H–K (**213**–**216**) and a related known compound cladodionen (**217**). The aniline moiety in cladosins H–K (**213**–**216**) was considered to be derived from the degradation of SAHA, indicating that the well-known histone deacetylase inhibitor SAHA could be metabolized by L3P3 and provide aniline as a precursor for the biotransformation of chemically reactive polyketides. Cladosin I (**214**) showed promising cytotoxicity against the HL-60 cell line with an IC_50_ value of 2.8 μM [95].

SAHA was found to significantly enhance the alkaloid production of *Claviceps purpurea* Cp-1 strain. Particularly, the titers of total ergot alkaloids gradually increased with the increase of SAHA concentration in the fermentation medium, and the highest production of ergot alkaloids could be achieved at the concentration of 500 μM SAHA. Specially, the titers of ergometrine (**218**) and total ergot alkaloids were as high as 95.4 mg/L and 179.7 mg/L, respectively, which were twice of those of the control. Furthermore, mRNA expression levels of the most functional genes in the ergot alkaloid synthesis (EAS) gene cluster were up-regulated under SAHA treatment. It was proposed that SAHA might increase histone acetylation in the EAS gene cluster region in the chromosome, which would loosen the chromosome structure and subsequently up-regulate the mRNA expression levels of genes involved in the biosynthesis of ergot alkaloids, thereby resulting in the marked increase in the production of ergot alkaloids [96].

The basidiomycete *Cyathus stercoreus* (Nidulariaceae) was treated with SAHA at 200 μM. Nine novel sesquiterpenoids were identified as cystercorolide (**219**), cystercorodiol A (**220**), 4-*O*-acetylcybrodol (**221**), 14-dehydroxycybrodol (**222**), cystercorodiol B (**223**), 4-*O*-acetylcystercorodiol B (**224**), 1-*O*-methoxycystercorodiol B (**225**), cystercorodiol C (**226**), and cystercorotone (**227**), along with four known sesquiterpenes, epicoterpene D (**228**), russujaponol F (**229**), riparol B (**230**), and cybrodol (**231**). Among these, cystercorodiol A (**220**), 4-*O*-acetylcybrodol (**221**), cystercorotone (**227**), and cybrodol (**231**) all at concentration of 200 μM showed weak antibacterial activity against *Escherichia coli* ATCC25922, with the inhibitory rates of 34.7%, 33.0%, 32.3%, and 29.6%, respectively [97].

A novel chlorinated pentacyclic polyketide, daldinone E (**232**) was induced from a *Daldinia* sp. fungal isolate treated with SAHA at 800 μM. Daldinone E (**232**) exhibited DPPH radical scavenging activities with potency comparable to the positive control, ascorbic acid [98].

SAHA (500 μM) was added in the medium of the dark septate endophytic fungus *Drechslera* sp., inducing the release of hexosylphytosphyngosine (**233**) to the culture medium [40].

When the endophytic fungus *Lachnum palmae* from *Przewalskia tangutica* was treated with SAHA at 500 μM, the production of eighteen dihydroisocoumarins, including seven previously undescribed halogenated ones, namely palmaerones A–G (**234**–**240**), along with 11 known ones, which were mellein (**18**), (3*R*)-6-hydroxymellein (**154**), (*R*)-6-methoxymellein (**157**) (*R*)-5-cholro-6-hydroxymellein (**241**), (3*R*,4*R*)-5-cholro-4,6-dihydroxymellein (**242**), palmaerin A (**243**), palmaerin B (**244**), palmaerin D (**245**), *trans*-4-hydroxymellein (**246**), *cis*-4-hydroxymellein (**247**), and (*R*)-5-hydroxymellein (**248**), was induced. Palmaerones A–G (**234**–**240**) were screened against three fungal strains (*Cryptococcus neoformans*, *Penicillium* sp. and *Candida albicans*) and two bacteria strains (*Bacillus subtilis* and *Staphylococcus aureus*). Among them, palmaerone E (**238**) exhibited potential antimicrobial activities against all the test strains, with MIC values in the range of 10–55 mg/mL. Generally, the brominated dihydroisocoumarins showed better antimicrobial activities than the chlorinated dihydroisocoumarins. Furthermore, palmaerones A (**234**) and E (**238**) exhibited moderate inhibitory effects on NO production in LPS-induced RAW 264.7 cells, with IC_50_ values of 26.3 mM and 38.7 mM, respectively, and no obvious toxicities were observed at 50 mM. Palmaerone E (**238**) showed weak cytotoxicity against HepG2 with an IC_50_ value of 42.8 mM [99].

SAHA at 100 μM was added into the cultures of *Microascus* sp. from the Floridian marine sediment. A cyclic pentadepsipeptide named EGM-556 (**249**) was isolated [42].

SAHA was added in the cultures of *Muscodor yucatanensis* Ni30 at 50 μM and led to the enhanced production of bioactive VOCs. Two main induced compounds, ergosterol (**104**) and xylaguaianol C (**105**), were isolated from the epivariant [69].

The cultures of plant endophytic fungus *Penicillium* sp. HS-11 in modified Martin’s medium supplemented with SAHA at 80 mg/L led to the isolation and identification of two induced metabolites, 4-epipenicillone B (**250**) and (*R*)-chrysogine (**251**) [100].

Cultivation of the endophytic fungus *Penicillium* sp. KMU18029, with the addition of SAHA at 100 μM, led to the isolation of two pairs of meroditerpenoids, pyrandecarurins A (**252**) and B (**253**) and pileotins A (**254**) and B (**255**), along with their potential precursor decaturenoid (**256**) as well as the known meroterpenoids 15-hydroxydecaturin A (**257**), oxalicines A (**258**) and B (**259**), and penisarin B (**260**). Decaturenoid (**256**) showed moderate activity against AChE with an IC_50_ value of 13.9 μM [101].

The addition of SAHA at 200 μM in the cultures of *Penicillium brasilianum* led to biosynthesis reduction of brasiliamide A (**261**), verruculogen TR2 (**262**), and penicillic acid (**162**) [27].

Four secondary metabolites, sclerotiorin (**108**), sclerotioramine (**113**), and isochromophilones XIV (**263**) and XV (**264**), were isolated from *Pencillium mallochii* CCH01 treated with SAHA at 1 mM. Sclerotiorin (**108**) showed broad and important inhibition activities against *Curvularia lunata*, *Curvularia clavata*, *Fusarium oxysporum* f. sp. *mornordica*, and *Botryosphaeria dothidea*, with IC_50_ values of 2.1 µg/mL, 21.0 µg/mL, 40.4 µg/mL, and 27.8 µg/mL, respectively. Both sclerotioramine (**113**) and isochromophilone XV (**264**) showed selective antifungal activity on *Colletotrichum graminicola*, with IC_50_ values of 29.9 µg/mL and 9.7 µg/mL, respectively. Furthermore, both sclerotiorin (**108**) and sclerotioramine (**113**) exhibited strong antibacterial activities on *Bacillus subtilis* [102].

Seven polyketides, including four new ones, namely varilactones A (**265**) and B (**266**) and wortmannilactones M (**267**) and N (**268**), as well as three biogenetically related known wortmannilactones E (**269**), F (**270**), and H (**271**), were isolated from the fungus *Penicillium variabile* HXQ-H-1 cultivated in potato-based medium with SAHA at 300 μM [103].

SAHA (500 μM) was added to the culture broth of the endophytic fungus *Phoma* sp. LG0217 isolated from *Parkinsonia microphylla*. The metabolite profile was changed and resulted in the production of (10′*S*)-verruculide B (**272**), vermistatin (**273**), and dihydrovermistatin (**274**). When the fungus was cultured in the absence of SAHA, it produced (*S*,*Z*)-5-(3′,4′-dihydroxybutyldiene)-3-propylfuran-2(5*H*)-one (**275**) and nafuredin (**276**). (10′S)-Verruculide B (**272**) showed inhibitory activity on protein tyrosine phosphatases (PTPs) [104].

*Spiromastix* sp., a deep-sea sediment-derived fungus, was treated with SAHA at 500 μM. Nine new guaine-type sesquiterpenes named spiromaterpenes A (**277**), B (**278**), C (**279**), D (**280**), E (**281**), F (**282**), G (**283**), H (**284**), and I (**285**) were isolated. Among them, spiromaterpenes D (**280**), E (**281**), and F (**282**) exhibited significant effects against NO production on lipopolysaccharide (LPS)-induced microglia cells BV2. In addition, spiromaterpene E (**281**) was the most active guaine-type sesquiterpene to show anti-neuroinflammatory activity [105].

A highly modified fatty acid ester named funitatin A (**286**) was firstly isolated from the Yellow River wetland-derived fungus *Talaromyces funiculosus* HPU-Y01 cultivated with 300 μM of SAHA. Funitatin A (**286**) featured a rare dimeric cyclopaldic acid structure and showed promising antimicrobial activity against both *Proteus* species and *Escherichia coli*, with MIC values of 3.13 μM [106].

*Talaromyces wortmannii* treated with SAHA at 100 μM resulted in the isolation of four new wortmannilactones derivatives, namely wortmannilactones I–L (**287**–**290**). These four compounds showed potent inhibitory activity against NADH-fumarate reductase, with IC_50_ values ranging from 0.84 to 1.35 μM [107].

Resveratrol (**132**) is an important stilbene that has a high demand due to its therapeutic, cosmeceutical, and nutraceutical activities. *Xylaria psidii* was an endophytic fungus isolated from the leaves of *Vitis vinifera*. The addition of SAHA (5 μM) to the medium of *Xylaria psidii* increased the production of resveratrol (**132**) [75].

Some fungal species such as *Aspergillus niger*, *Botryosphaeria mamane*, *Cladosporium reesinae*, and *C. reesinae* were also treated with SAHA, and the production of their secondary metabolites was induced. However, the induced metabolites were not identified. Treatment of SAHA at 100 μM to the cultures of *A. niger* induced the production of new secondary metabolites confirmed by HPLC, but they were not further identified [108]. The cultures of *Botryosphaeria mamane* were treated with SAHA at 100 μM to induce the production of eight main unidentified metabolites detected by HPLC [44]. The treatment of SAHA at 10 μM of the cultures of *C. reesinae* NRL-6437 increased the production of antimicrobial metabolites, which were not further structurally identified [76]. The cultures of *Hypoxylon* sp. CI-4 were treated with SAHA at 50 μM. The production of VOCs was induced, and they were preliminarily identified as terpenes, alkanes, alkenes, organic acids, and benzene derivatives by GC-MS [77].

### 4.2. Effects of Suberoylbishydroxamic Acid

Suberoylbishydroxamic acid (SBHA) is also called suberohydroxamic acid, and its structure is similar to that of SAHA. Some examples of SBHA affecting the production of fungal secondary metabolites are listed in Table S3. The structures of compounds **291**–**351** isolated from fungi treated with SBHA are shown in Figure S5.

Supplementation of SBHA (500 μM) to the culture medium of the endophytic fungus *Alternaria* sp. induced the production of mycotoxins, including alternariol (**26**), alternariol-5-*O*-methyl ether (**27**), 3′-hydroxyalternariol-5-*O*-methyl ether (**28**), altenusin (**29**), tenuazonic acid (**30**), and altertoxin II (**31**) [51].

The addition of SBHA at 500 μM to the culture medium of *Arthrobotrys foliicola* induced the production of a coumarin-type secondary metabolite represented by a single intensive peak in the HPLC profile of the ethyl acetate extract. The compound was identified as 4-ethyl-7-hydroxy-8-methyl-2*H*-chromen-2-one (**291**) [109].

The addition of SBHA at 500 μM in the cultures of *Chaetomium indicum* led to the production of chaetophenols A–F (**292**–**297**) [110]. Two spirolactone polyketides spiroindicumides A (**298**) and B (**299**) were also isolated from *C. indicum* cultivated in the presence of SBHA at 500 μM [111].

The treatment of *Cladosporium cladosporioides* with SBHA at 0.1–10 mM elicited the production of cladochromes A (**208**), B (**209**), D (**210**), E (**211**), F (**206**), and G (**207**), and calphostin B (**212**) [26].

The addition of SBHA at 500 μM to the culture medium of *Cladosporium colocasiae* dramatically altered the production of two new acetylenic sterols, namely (3β,7α)-cholest-5-en-23-yne-3,7-diol (**300**) and (3β,7α)-cholest-5-en-23-yne-3,7,25-triol (**301**). (3β,7α)-Cholest-5-en-23-yne-3,7-diol (**300**) showed antibacterial activity against *Bacillus subtilis*, affording a zone of inhibition of 12 mm at 100 μg/disk [112]

Four 2,3-dihydrobenzofurans, annullatins A–D (**302**–**305**), and one aromatic polyketide, annullatin E (**306**), were isolated from the entomopathogenic fungus *Cordyceps annullata* by the addition of SBHA at 500 μM in the medium [113].

Six novel aromatic polyketides, namely indigotides C–F (**307**–**310**), 13-hydroxyindigotide A (**311**), and 8-*O*-methylindigotide B (**312**) along with indigotides A (**94**) and B (**95**), were induced from the entomopathogenic fungus *Cordyceps indigotica* by the addition of SBHA at 1 mM in the medium [114].

The cultivation of a deep-sea-derived fungus *Eutypella* sp. MCCC 3A00281 by SBHA at 1 mM led to the isolation of 26 eremophilane-type sesquiterpenoids, namely eutyperemophilanes A–Z (**313–338**). Among these compounds, eutyperemophilanes I (**321**) and J (**322**) showed significant inhibitory effects on the nitric oxide (NO) production that was induced by lipopolysaccharide (LPS) in RAW 264.7 macrophage cells [115].

*Fusarium oxysporum* sp. *conglutinans* was treated with SBHA at 500 μM. Two fusaric acid derivatives, namely 5-butyl-6-oxo-1,6-dihydropyridine-2-carboxylic acid (**339**) and 5-(but-9-enyl)-6-oxo-1,6-dihydropyridine-2-carboxylic acid (**340**), were induced [116].

The addition of SBHA (1 mM) to the culture medium of *Gibellula formosana* significantly enhanced the production of isariotin A (**341**) [38]

The addition of SBHA (500 μM) to the culture medium of *Paraconiothyrium brasiliense* activated the production of one pyridinone named JBIR-54 (**342**) [117].

Seven metabolites, including a new one, 13-angeloyloxy-diplosporin (**343**), and six known ones 3-methoxymethyl-agistatine D (**344**), gynuraone (**345**), mellein (**18**), cytosporone B (**346**), dothiorelone A (**347**), and dothiorelone C (**348**), were isolated from the endophytic fungus *Phomopsis* sp. 0391 that was cultivated in the presence of SBHA at 500 μM. Both cytosporone B (**346**) and dothiorelone A (**347**) displayed significant lipase inhibitory activities with IC_50_ values of 115 μg/mL and 275 μg/mL, respectively, compared to the positive control (tetrahydrolipstatin, IC_50_, 43 μg/mL) [118].

The addition of SBHA to the medium at 1 μM led to significant changes in the secondary metabolite profile of the entomopathogenic fungus, *Torrubiella luteorostrata*, and induced production of three new prenylated tryptophan analogs, luteorides A–C (**349**–**351**) [119].

### 4.3. Effects of Valproic Acid and Sodium Valproate

Both valproic acid (VPA) and sodium valproate (SVP) have very similar structures and the same epigenetic regulation effects [44]. Some examples of VPA or SVP that affect the production of fungal secondary metabolites are listed in Table S4. The structures of compounds **352**–**371** isolated from fungi treated with VPA or SVP are shown in Figure S6.

When the cultures of *Aspergillus clavatus* were treated with VPA at 60 μM, cytochalasin E (**38**) was significantly enhanced production [25].

VPA at 500 μM induced the production of fumiquinazoline C (**352**) in the endophytic fungus *Aspergillus fumigatus* GA-L7 isolated from *Grewia asiatica* L. It was further revealed that all the genes involved in the biosynthesis of fumiquinazoline C (**352**) were overexpressed significantly, resulting in the overall enhancement of fumiquinazoline C (**352**) production by about ten-fold [120].

The weekly supplementation of VPA at 50 μM to the cultures of *Cordyceps militaris* significantly improved cordycepin (**353**) production by 41.2% compared to the untreated control, and the gene regulatory network of *C. militaris* was also adapted [121].

The addition of VPA (100 μM) to the cultures of the endophytic fungus *Diaporthe* sp. isolated from *Datura inoxia* significantly altered its secondary metabolic profile and resulted in the isolation of three novel cytotoxic secondary metabolites, namely xylarolide A (**354**), diportharine A (**355**), and xylarolide B (**356**), along with one known compound, xylarolide (**357**). Among these compounds, both xylarolide A (**354**) and xylarolide (**357**) displayed significant growth inhibition on pancreatic cancer MIAPaCa-2 cells with IC_50_ values of 20 μM and 32 μM, respectively, and against prostate cancer PC-3 cells with IC_50_ values of 14 μM and 18 μM, respectively. Moreover, xylarolide A (**354**) displayed significant DPPH scavenging activity with an EC_50_ value of 10.3 μM [122].

When the endophytic fungus *Diaporthe* sp. PF20 from *Piper nigrum* was treated with VPA at 100 μM, piperine (**358**) production was enhanced [123].

*Dimorphosporicola tragani* CF-090383 was examined to produce three mycotoxins when VPA was added to the fermentation at 100 μM. The induced mycotoxins were identified as dendrodolides E (**99**), G (**100**), and I (**101**) [67].

The treatment of VPA at 50 μM in the cultures of *Doratomyces microspora* resulted in the enhanced production of seven antimicrobial compounds, *p*-hydroxy benzaldehyde (**359**), phenyl acetic acid (**360**), phenyllactic acid (**361**), indole-3-carboxylic acid (**362**), indole-3-acetic acid (**363**), cyclo-(proline-methionine) (**364**), and cyclo-(phenylalanine-proline) (**365**) [27].

The cultures of *Drechslera* sp. were treated with VPA at 500 μM. The production of benzophenone (**366**) was increased [40].

Incorporating SVP at 1 mM affected the metabolite profile of the endophytic fungus *Macrophomina phaseolina* from the roots of *Brugmasnsia aurea*. Two compounds induced production and were identified as 3-acetyl-2-methyl dihydro-furan-2(3*H*)-one (**367**) and 2-methyl-3-methylthio-butanoic acid (**368**) [124].

By treating with SVP at 10 μM in the sponge-associated *Penicillium chrysogenum* HLS111, three new heterodimeric tetrahydroxanthone–chromanone lactones, chrysoxanthones A–C (**369**–**371**), were isolated. They exhibited moderate antibacterial activities against *Bacillus subtilis* with MIC values of 5–10 µg/mL [125].

The induced compounds by SVP or VPA in the cultures of *Botryosphaeria mamane* and *Phomopsis heveicola* were only detected by LC-MS or GC-MS and were not further identified. *Botryosphaeria mamane* was an endophytic fungus isolated from *Bixa orellana*. An addition of SVP at 100 μM induced the production of two metabolites in the cultures of *B. mamane* by LC-MS analysis [44]. VPA at 0.5–25 μg/mL increased the production of volatile compounds secreted by the endophytic fungus *P. heveicola* of the tropical plant *Piper longum*. These increased volatile compounds were only preliminarily identified by GC-MS analysis [126].

### 4.4. Effects of Sodium Butyrate

Some examples of sodium butyrate (NaBut) that affect the production of fungal secondary metabolites are listed in Table S5. The structures of compounds **372**–**410** isolated from fungi treated with NaBut are shown in Figure S7.

NaBut at 9 μM significantly increased the production of cytochalasin E (**38**), patulin (**39**), and pseurotin A (**40**) compared to the control in the suspension culture of *Aspergillus clavaus* [25].

Two novel brominated resorcylic acid lactones, namely 5-bromozeaenol (**372**) and 3,5-dibromozeaenol (**373**), together with four known analogues, aigialomycin B (**374**), zeaenol (**375**), LL-Z1640-1 (**376**), and LL-Z1640-2 (**377**), were produced by the marine-derived fungus *Cochliobolus lunatus* TA26-46 treated with NaBut at 10 mM [127].

When *Diaporthe* sp. PF20, the endophytic fungus from *Piper nigrum*, was treated with NaBut at 100 μM, the production of piperine (**358**) was enhanced [123].

When NaBut was added in the medium of the mangrove-derived endophytic fungus *Leucostoma persoonii* at 100 μM, the production of cytosporones B (**346**), C (**378**), and E (**379**) was enhanced, and the novel cytosporone R (**380**) was induced. These cytosporones showed antibacterial activities on methicillin-resistant *Staphylococcus aureus* (MRSA) [128].

The marine-derived *Penicillium brevicompactum* was treated with NaBut at 10 mM. The production of both anthranilic acid (**381**) and ergosterol peroxide (**382**) was enhanced [129].

The addition of NaBut at 1 mM in the medium of endophytic fungus *Phomopsis* sp. XP-8 isolated from the bark of Tu-Chung (*Eucommia ulmoides*) decreased yields of pinoresinol (**383**), pinoresinol monoglucoside (**384**), and pinoresinol diglucoside (**385**) [130].

Two new compounds, named phaseolorin J (**386**) and phomoparagin D (**387**), along with three known chromones, phaseolorin D (**388**), chaetochromone B (**389**), and pleosporalin D (**390**), and six known compounds, cytochalasins J (**391**), J1 (**392**), J2 (**393**), J3 (**394**), H (**395**), and phomopchalasin D (**396**), were isolated from the cultures of *Phomopsis asparagi* DHS-48 treated with NaBut at 50 μM. Both phaseolorin J (**386**) and cytochalasin J2 (**393**) moderately inhibited the proliferation of concanavalin A-induced T and lipopolysaccharide-induced B murine spleen lymphocytes. Phomoparagin D (**387**) exhibited significant in vitro cytotoxicity against the tested human cancer cell lines HeLa and HepG2, which was comparative to the positive controls adriamycin and fluorouracil [131].

When the coral-derived fungus *Trichoderma harzianum* XS-20090075 was treated with 10 μM NaBut, the production of terpenoids was induced, including three new terpenoids, namely harzianolic acid (**397**), harzianone E (**398**), and 3,7,11-trihydroxy-cycloneran (**399**), together with 11 known sesquiterpenoids, methyl 3,7-dihydroxy-15-cycloneranate (**400**), catenioblin C (**401**), ascotrichic acid (**402**), cyclonerotriol (**403**), (10*E*)-12-acetoxy-10-cycloneren-3,7-diol (**404**), cyclonerodiol (**405**), cyclonerodiol oxide (**406**), epicyclonerodiol oxide (**407**), ophioceric acid (**408**), *ent*-trichoacorenol (**409**), and trichoacorenol (**410**). Both harzianone E (**398**) and methyl 3,7-dihydroxy-15-cycloneranate (**400**) exhibited weak antibacterial activity against *Photobacterium angustum* [132].

### 4.5. Effects of Nicotinamide

Nicotinamide belongs to the NAD^+^-dependent HDAC inhibitor. Some examples of nicotinamide affecting the production of fungal secondary metabolites are listed in Table S6. The structures of compounds **411**–**439** isolated from fungi treated with nicotinamide are shown in Figure S8.

The addition of nicotinamide at 62.5 μg/mL in the cultures of *Aspergillus awamori* induced the production of secondary metabolites based on LC-MS analysis. Some differential metabolites were speculated according to the accurate molecular weight data. These putative metabolites need further identification [133].

Nicotinamide at 50 μM was added to the medium, in which the fungus *Chaetomium cancroideum* was cultured. It significantly enhanced the production of five polyketides, including chaetophenols B (**293**) and C (**294**), chaetophenol G (**411**), and cancrolides A (**412**) and B (**413**) [134].

The cultivation of *Chaetomium mollipilium* with nicotinamide at 100 μM stimulated its secondary metabolism, leading to the production of new polyketides, mollipilin A–E (**414**–**418**), along with two known compounds, mollipilin F (**419**) and aureonitol (**420**). Both mollipilins A (**414**) and B (**415**) exhibited moderate growth inhibitory effects on human colon cancer (HCT-116) cells [135].

The endophytic fungus *Eupenicillium* sp. LG41 from the Chinese medicinal plant *Xanthium sibiricum* was treated with nicotinamide at 1.5 mg/L. Two decalin-containing compounds, eupenicinicols C (**421**) and D (**422**), along with their biosynthetic precursors, eujavanicol A (**423**) and eupenicinicol A (**424**), were isolated. Among them, eupenicinicol D (**422**) was active against the bacterium *Staphylococcus aureus* with an MIC value of 0.1 μg/mL and also showed marked cytotoxicity against the human acute monocytic leukemia cell line THP-1 [136].

The production of both ergosterol peroxide (**382**) and deoxynivalenol (DON, **425**) was significantly reduced by nicotinamide at 500 μg/mL in *Fusarium* head blight pathogen *Fusarium graminearum* of wheat plants [137].

Cultures of the endophytic fungus *Graphiopsis chlorocephala* from *Paeonia lactiflora* were treated with nicotinamide at 10 μM. This led to the activated production of benzophenones, which were identified as cephalanones A–F (**426**–**431**) and 2-(2,6-dihydroxy-4-methylbenzoyl)-6-hydroxybenzoic acid (**432**) [138].

The addition of nicotinamide at 100 μM in the cultures of *Penicillium brasilianum* led to the decreased production of brasiliamide A (**261**), verruculogen TR2 (**262**), and penicillic acid (**162**) [27].

The production of nine phenolic metabolites, namely *p*-hydroxybenzaldehyde (**359**), phenyl acetic acid (**360**), *p*-anisic acid (**433**), *p*-anisic acid methyl ester (**434**), benzyl anisate (**435**), syringic acid (**436**), sinapic acid (**437**), acetosyringone (**438**), and gentisaldehyde (**439**) was induced by nicotinamide at 100 μM in fermentation of the marine-derived fungus *Penicillium brevicompactum*. Among them, syringic acid (**436**), sinapic acid (**437**), and acetosyringone (**438**) exhibited potent in vitro free radical scavenging with IC_50_ values from 20 to 30 µg/mL and antiproliferative activities with IC_50_ values from 1.14 to 1.71 µM against the HepG2 cancer cell line [129].

### 4.6. Effects of Trichostatin A

The structures of compounds **440**–**451** isolated from fungi treated with trichostatin A (TSA) are shown in Figure S9. TSA at 1 μM was found to increase the production of secondary metabolites in the cultures of *Alternaria alternata* and *Penicillium expansum* with TLC examination. However, the increased compounds were not further identified [28].

TSA at 0.5 μM significantly increased the production of cytochalasin E (**38**), patulin (**39**), and pseurotin A (**40**) in the cultures of *Aspergillus clavatus* [25].

The histone deacetylase gene *rpdA* expression was stimulated by TSA at 1 μM in *Aspergillus nidulans*. Unfortunately, the fungal secondary metabolism was not further studied [139].

Four new meroterpenoids identified as (*R*)-4-((2,2-dimethylchroman-6-yl)methyl)-3-(4-hydroxyphenyl)-5-methoxyfuran-2(5*H*)-one (**440**), 1-(2,2-dimethylchroman-6-yl)-3-(4-hydroxyphenyl)propan-2-one (**441**), (*R*,*E*)-3-(2,2-dimethylchroman-6-yl)-4-hydroxy-5-((2-(2-hydroxypropan-2-yl)-2,3-dihydrobenzofuran-5-yl)methylene)furan-2(5*H*)-one (**442**), and methyl (*R*)-2-(2-(2-hydroxypropan-2-yl)-2,3-dihydrobenzofuran-5-yl) acetate (**443**), along with nine known compounds, including ergosterol (**104**), flavipesolides A–C (**444**–**446**), rubrolide S (**447**), 5-[(3,4-dihydro-2,2-dimethyl-2H-1-benzopyran-6-yl)-methyl]-3-hydroxy-4(4-hydroxyphenyl)-2(5*H*)-furanone (**448**), (3*R*,4*R*)-3,4-dihydro-4,8-dihydroxy-6,7-dimethoxy-3-methylisocoumarin (**449**), (3*R*)-3,4-dihydro-6,8-dimethoxy-3-methylisocoumarin (**450**), and terretonin C (**451**), were isolated from the cultures of *Aspergillus terreus* OUCMDZ-2739 with 10 μM of TSA in the medium. Under the same condition, without TSA, *A. terreus* OUCMDZ-2739 produced different compounds, supporting that the chemical-epigenetic modification of fungi could enrich the chemodiversity of the fungal products [140].

TSA was applied to the liquid medium of *Trichoderma atroviride* at 300 nM. The production of antimicrobial compounds was induced, and the expression of two secondary metabolism-related genes *pbs-1* and *tps-1*, which encoded a peptaibol synthase and a terpene synthase, respectively, were activated. The induced antimicrobial compounds could be further identified [43].

### 4.7. Effects of Other Histone Deacetylase Modifiers

Except for the histone deacetylase (HDAC) modifiers mentioned above, other HDAC modifiers, including dihydrocoumarin (DHC), entinostat (MS-275), 2-hexyl-4-pentynoic acid, 5-methylemellein, quercetin, and octanoylhydroxamic acid (OHA), were also screened to have obvious effects on fungal secondary metabolism. Some examples of other histone deacetylase modifiers affecting the production of fungal secondary metabolites are listed in Table S7. The structures of compounds **452**–**462** isolated from fungi treated with other histone deacetylase modifiers are shown in Figure S10.

Dihydrocoumarin was an inhibitor of the sirtuin family of NAD^+^-dependent histone deacetylases. When the cultures of *Monascus ruber* M7 were treated with dihydrocoumarin at 5 mM, the production of monasfluol B (**452**), acetyl-monasfluol B (**453**), and azaphilone C (**454**) increased. However, the production of citrinin (**455**) decreased [45].

*Hypomyces* sp. CLG4 was cultured in the presence of entinostat (MS-275) at 500 μM. Six metabolites were significantly induced and identified as (3*R*)-6-hydroxymellein (**154**), (3*R*)-6-methoxymellein (**157**), 6-demethylkigelin (**456**), (3*R*)-6,7-dimethoxymellein (**457**), pyrolin (**458**), and terrain (**459**) [141].

When the fungus *Aspergillus versicolor* was treated with 2-hexyl-4-pentynoic acid (HPTA) at concentrations of 12.5 mg/L, 37.5 mg/L, and 62.5 mg/L, respectively, the production of three metabolites were greatly increased when HPTA was added in the medium at 37.5 mg/L. They were identified as diorcinol (**53**), curvularin (**460**), and cyclo-(L-Trp-L-Phe) (**461**) [48].

Sirtuin is an NAD^+^-dependent histone deacetylase (HDAC) that is highly conserved in prokaryotes and eukaryotes. 5-Methylmellein and its structurally related compound, mellein, inhibited SirA activity with IC_50_ values of 120 µM and 160 µM, respectively. Adding 5-methylmellein to *Aspergillus nidulans* cultures increased the production of secondary metabolites. Unfortunately, the stimulated metabolites were not identified [46].

The cultures of *Drechslera* sp. were treated with octanoylhydroxamic acid (OHA) at 500 μM. The production of benzophenone (**366**) increased [40].

Quercetin, which was at a concentration of 100 μM, induced the biosynthesis of vinblastine (**462**) as a target product in the endophytic fungi *Aspergillus amstelodami* VR177L and *Penicillium concavoradulozum* VE89L [142].

### 4.8. Effects of Combinational Treatment with Two Histone Deacetylase Modifiers

The structures of compounds **463** and **464** isolated from fungi treated with two histone deacetylase modifiers are shown in Figure S11. Nicotinamide is a class III inhibitor of HDAC, and SAHA is a class I and II inhibitor of HDAC. Under the combination addition of SAHA (200 μM) and nicotinamide (100 μM) in the cultures of *Penicillium brasilianum*, penicillic acid (**162**) production was significantly suppressed [27].

When the fungus *Stagonospora nodorum* was co-treated with 50 μM of nicotinamide and 500 μM of SAHA, the production of alternariol (**26**), 4’-methoxy-(2*S*)-methylbutyrophenone (**463**), and (3*R*)-mellein methyl ether (**464**) was induced [143].

## 5. Effects of Other Chemical Epigenetic Modifiers

Other effective chemical epigenetic modifiers screened for a fungal secondary metabolism included histone acetyltransferase modifiers (i.e., anacardic acid) [31], histone methyltransferase modifiers (i.e., BRD4770) [32], proteasome modifiers (i.e., bortezomib) [33,144], and modifiers with unclear mechanisms (i.e., NPD938) [34,49].

### 5.1. Effects of Histone Acetyltransferase Modifier Anacardic Acid

Anacardic acid was a histone acetyltransferase inhibitor and was first found in the nutshells of *Anacardium occidentale* [145]. The structures of compounds **465**–**467** isolated from fungi treated with anacardic acid are shown in Figure S12.

In fermentation culture of the endophytic fungus *Anteaglonium* sp. FL0768, anacardic acid at 500 μM slightly affected the metabolite profile, affording scorpinone (**465**) as the major metabolite together with 1-hydroxydehydroherbarin (**466**) and a different methylated hexaketide, ascochitine (**467**) [31].

### 5.2. Effects of Histone Methyltransferase Modifier BRD4770

Methyl-2-(benzoylamino)-1-(3-phenylpropyl)-1*H*-benzimidazole-5-carboxylate, which was named BRD4770, is a histone methyltransferase inhibitor. The structures of compounds **468**–**470** isolated from fungi treated with BRD4770 are shown in Figure S13.

The crude extract of the endophytic fungus *Diaporthe longicolla* was found to have potent antioxidant and antibacterial activity, which were selected for the treatment of the epigenetic modulator BRD4770. The dose of 100 nM BRD4770 used to treat the cultures of endophytic fungus *D. longicolla* was noted as an effective concentration in inducing the isolation of bioactive cryptic metabolites, thereby increasing antibacterial and antioxidant activities. A comparative study of BRD4770-treated and non-treated crude chromatograms of RP-HPLC with standard solutions of berberine (**468**), caffeine (**469**), and theobromine (**470**) confirmed the presence of respective compounds in treated cultures. This study successfully established the importance of BRD4770, which also interacted with epigenetic targets and significantly induced and downregulated the production of cryptic metabolites in the endophytic fungus *D. longicolla* [32].

### 5.3. Effects of Proteasome Modifier Bortezomib

Many natural products were screened to have proteasome regulatory activities. However, they were rarely used for the regulation of fungal secondary metabolism [18,20]. The structures of compounds **471**–**475** isolated from fungi treated with proteasome modifier bortezomib are shown in Figure S14.

The addition of the proteasome modifier bortezomib at 300 μM to the fermentation broth of the sponge-derived fungus *Pestalotiopsis maculans* 16F-12 led to the isolation of four new bergamotene sesquiterpenes, xylariterpenoids H–K (**471**–**474**), which belong to sesquiterpenoids [144].

The fungus *Pleosporales* sp. was treated with bortezomib at 125 μg/mL. An additional metabolite was isolated and identified as (*R*)-2-(2-hydroxypentyl)-5-carboxy-7-methoxychromone (**475**) [33].

### 5.4. Effects of the ModifierNPD938 with Unclear Mechanisms

NPD938 was an epigenetic modifier with an unclear action mechanism. The structures of the compounds **476**–**486** isolated from fungi treated with NPD938 are shown in Figure S15.

The addition of NPD938 at 30 μM to the cultures of *Fusarium* sp. RK97-94 led to the induced production of three lucilactaene analogures, namely dihydroNG391 (**476**), dihydrolucilactaene (**477**), and 13α-hydroxylucilactaene (**478**). Among these, dihydroNG391 (**476**) exhibited weak in vitro antimalarial activity (IC_50_ value as 62 μM). Both dihydrolucilactaene (**477**) and 13α-hydroxylucilactaene (**478**) showed very potent antimalarial activity (IC_50_ values of 0.0015 μM and 0.68 μM, respectively) on *Plasmodium falciparum.* The structure–activity relationship showed that the removal of epoxide from NG391 (**479**) to obtain dihydrolucilactaene (**477**) resulted in a 1200-fold increase of antimalarial activity, suggesting that this epoxide was extremely detrimental for antimalarial activity. In addition, the opening of the tetrahydrofuran ring of 13α-hydroxylucilactaene (**478**) to form dihydrolucilactaene (**477**) resulted in a 100-fold increase of activity, confirming that the tetrahydrofuran ring was not more important for activity than the intact pyrrolidone ring and removal of epoxide. Furthermore, dihydrolucilactaene (**477**) exhibited weak cytotoxic activity against HeLa and HL-60 cells with IC_50_ values of 21 μM and 37 μM, respectively [49].

NPD938 enhanced tenuazonic acid (**30**) production in the rice blast fungus *Pyricularia oryzae* [34]. NPD938 also induced the production of *P*-pyridoxatin (**480**), *M*-pyridoxatn (**481**), F14329 (**482**), terpendoles C (**483**) and K (**484**), paspaline (**485**), and emindole SB (**486**) in the fungus *Tolypocladium album* [34].

## 6. Effects of Two Types of Chemical Epigenetic Modifiers

Some examples of two types of chemical epigenetic modifiers affecting the production of fungal secondary metabolites are listed in Table S8. The structures of compounds **487**–**570** isolated from fungi treated with two types of chemical epigenetic modifiers are shown in Figure S16.

The supplementation of 5-Aza (250 μM) and SAHA (500 μM) to the culture medium of the endophytic fungus *Alternaria* sp. induced the production of mycotoxins, including alternariol (**26**), alternariol-5-*O*-methyl ether (**27**), 3′-hydroxyalternariol-5-*O*-methyl ether (**28**), altenusin (**29**), tenuazonic acid (**30**), and altertoxin II (**31**). Furthermore, in the presence of both 5-Aza (250 μM) and SAHA (500 μM) in the medium of *Alternaria* sp., the yield of tenuazonic acid (**30**) from the endophytic fungus more than doubled, as compared with the only 5-Aza (250 μM) or SAHA (500 μM) addition in the medium [51].

Three new eremophilane-type sesquiterpenes, dihydrobipolaroxin B (**487**), dihydrobipolaroxin C (**488**), and dihydrobipolaroxin D (**489**), along with one known analogue, dihydrobipolaroxin (**490**), were isolated from the cultures of the deep marine-derived fungus, *Aspergillus* sp. SCSIOW2, treated with a combination of 1 mM of SBHA and 1 mM of 5-Aza. All four dihydrobipolaroxins exhibited moderate nitric oxide inhibitory activities [146].

One new diphenylether-*O*-glycoside named diorcinol 3-*O*-α-D-ribofuranoside (**491**) along with seven known compounds, (7*S*)-sydonic acid (**33**), (*S*)-sydowic acid (**37**), diorcinal (**492**), 3,3′-dihydroxy-5,5′-dimethyldibenzofuran (**493**), cordyol (**494**), gibellulin B (**495**), and cyclo-(L-Trp-L-Phe) (**496**), were isolated from the culture of the deep marine-derived fungus *Aspergillus* sp. SCSIOW3 treated with a combination of 1 mM of SBHA and 1 mM of 5-Aza. Both diorcinal (**492**) and cordyol (**494**) exhibited significant biomembrane protective effects on erythrocytes. Diorcinal (**492**) also showed algicidal activity against *Chattonella marina*, a bloom forming alga responsible for large-scale fish deaths [147].

The combination of *N*-acetyl-D-glucosamine (GlcNAc) (0.5 μM) with sodium butyrate (NaBut) (9 μM) significantly increased production of pseurotin A (**40**) compared to the control in the cultures of *Aspergillus clavatus* [25].

5-Aza (500 μM) in combination with SBHA (500 μM) was applied on an endophytic fungus *Aspergillus fumigatus* isolated from the terrestrial plant *Cynodon dactylon*. They significantly changed the metabolic profile and resulted in the production of nineteen compounds, including ten alkaloids: 3-dehydroxymethylbisdethio-3,10a-bis(methylthio) gliotoxin (**497**), bisdethiobis (methylthio) gliotoxin (**498**), fumitremorgin C (**499**), fumiquinazoline J (**500**), pyripyropene A (**501**), chaetominine (**502**), fumigaclavine A (**503**), 9-deacetoxyfumigaclavine C (**504**), fumigaclavine C (**505**), and 3-hydroxyacetyl indole (**506**); six polyketides: helvolinic acid (**507**), rhizoctonic acid (**508**), monomethylsulochrin (**509**), 3-hydroxy-1,6-dimethoxy-8-methyl 9*H*-xanthen-9-one (**510**), questin (**511**), and 6-hydroxy-8-methoxy-3-methylisocoumarin (**512**); and three benzene derivatives: circinoporic acid (**513**), koaburaside (**514**), and methyl shikimate (**515**). Among them, three known alkaloids, namely bisdethiobis (methylthio) gliotoxin (**498**), fumitremorgin C (**499**), and 3-(hydroxyacetyl) indole (**501**), were enhanced in production to show immunosuppressive activities. Three compounds, namely helvolinic acid (**507**), 6-hydroxy-8-methoxy-3-methylisocoumarin (**512**), and koaburaside (**514**), belonged to the newly induced metabolites. Other compounds were increased for their production [148].

The supplementation of SAHA (100 µM) and 5-Aza (100 µM) to Czapek-Dox liquid medium of the endophytic fungus *Aspergillus versicolor* induced the production of 17 compounds, including two new nucleoside derivatives, kipukasins K (**516**) and L (**517**), and one new bisabolane sesquiterpene, aspergillusene E (**518**), along with four known nucleoside derivatives, kipukasin I (**519**), kipukasin H (**520**), kipukasin D (**521**), and kipukasin E (**522**), and ten known bisabolane sesquiterpenes, (7*R*)-hydroxysydonic acid (**32**), (7*S*)-sydonic acid (**33**), (*E*)-5-(hydroxymethyl)-2-(6′-methylhept-2′-en-2′-yl)phenol (**523**), (*Z*)-5-(hydroxymethyl)-2-(6′-methylhept-2′-en-2′-yl)phenol (**524**), 7-deoxy-7,14-didehydrosydonol (**525**), (7*R*)-sydonol (**526**), (7*R*)-methoxysydonol (**527**), (7S)-sydonol (**47**), (7S)-methoxysydonol (**528**), and aspergiterpenoid A (**529**). Both kipukasins K (**516**) and aspergillusene E (**518**) displayed antibacterial activities against *Staphylococcus epidermidis* and *Staphylococcus aureus* with MIC values of 8–16 µg/mL [149].

The deep-sea-derived fungus *Eutypella* sp. by the co-treatment with 5-Aza (1 mM) and SBHA (1 mM) resulted in the activation of a sesquiterpene-related biosynthetic gene cluster to produce at least 21 sesquiterpenes, including 17 undescribed eutypeterpenes A-Q (**530**–**546**). Four known sesquiterpenes were identified as xylariterpenoids A (**547**) and B (**548**), eudesma-3-en-11,15-diol (**549**), and eudesma-4-en-11,15-diol (**550**). Among the compounds, eutypeterpene N (**543**) was the most active to inhibit LPS-induced NO production in RAW 264.7 macrophage cells with an IC_50_ value of 8.6 μM. Ten compounds, eutypeterpenes B (**531**), C (**532**), E (**534**), M (**542**), O (**544**), P (**545**), and Q (**546**), xylariterpenoids A (**547**), eudesma-3-en-11,15-diol (**549**), and eudesma-4-en-11,15-diol (**550**), showed similar inhibitory effects, with IC_50_ values from 11.5 μM to 18.7 μM against NO production compared to that (IC_50_, 17.0 μM) of the positive control quercetin [150].

The concomitant addition of SBHA (1 mM) and RG-108 (1 mM) to the cultures of *Gibellula formosana*, an entomopathogenic fungus, induced the production of two new highly oxidized ergosterols, formosterols A (**551**) and B (**552**), and five new isariotin analogs, 12′-*O*-acetylisariotin A (**553**), 1-*epi*-isariotin A (**554**), and isariotins K–M (**555**–**557**), together with six known compounds, isariotin A (**341**), formosterol C (also named 22,23-epoxy-3,12,14,16-tetrahydroxyergosta-5,7-dien-11-one, **558**), isariotin C (**559**), isariotin E (**560**), TK-57-164A (**561**), and beauvericin (**562**) [38].

The concomitant addition of SBHA (500 μM) and RG–108 (500 μM) to the culture medium of the entomopathogenic fungus *Isaria tenuipes* led to the isolation of a novel polyketide tenuipyrone (**563**) along with two plausible precursors, cephalosporolides B (**564**) and F (**565**) of tenuipyrone (**563**) [151].

The concomitant addition of 5-Aza (50 μM) and sodium butyrate (100 μM) to the culture medium of marine fungus *Leucostoma persoonii* altered the production of cytosporones B (**346**), C (**378**), and E (**379**), as well as the production of the previously undescribed cytosporone R (**380**). Cytosporone E (**379**) displayed inhibitions with an IC_90_ value of 13 μM toward the severe malaria *Plasmodium falciparum* and an MIC value of 72 μM against methicillin-resistant *Staphylococcus aureus* (MRSA) [128].

The combination of 5-Aza (50 μM) with SAHA (50 μM) inhibited mycelial growth rate and pigmentation, and enhanced the production of bioactive VOCs, ergosterol (**104**), and xylaguaianol C (**105**) in the cultures of *Muscodor yucatanensis* Ni30 [69].

Concomitant supplementation of SAHA (500 μM) and 5-Aza (500 μM) to the culture medium of the plant endophytic fungus, *Pestalotiopsis acaciae*, dramatically altered its metabolic profiles. Three novel aromatic compounds, 20-hydroxy-6′-hydroxymethyl-4′-methylphenyl-2,6-dihydroxy-3-(2-isopentenyl)benzoate (**566**), 4,6-dihydroxy-7-hydroxymethyl-3-methylcoumarin (**567**), and 4,6-dihydroxy-3,7-dimethylcoumarin (**568**) were identified [152].

The production of two glycolipids, ustilagic acids B (**569**) and C (**570**), was induced when 5-Aza (500 μM) and SBHA (500 μM) were supplemented into the liquid medium of *Ustilago maydis*. Both glycolipids displayed weak antifungal activities against *Aspergillus terreus* and *Candida albicans* [153].

The addition of 5-Aza (5 μM) and SAHA (10 μM) to the cultures of *Xylaria psidii* increased the production of resveratrol (**132**) [75].

## 7. Conclusions

In summary, chemical-epigenetic modifiers can effectively trigger silent or low-expressed biosynthetic pathways of fungal secondary metabolites. Since the cultures of *Alternaria alternata* and *Penicillium expansum* treated with trichostatin A to activate the production of secondary metabolites were first reported by the group of Nancy P. Keller in 2007 [28], great progress has been achieved. The most impressive advantage of using chemical epigenetic modifiers is that there is no need to know the target genome features. Furthermore, this low-cost technique is relatively easy to apply in high-throughput screening operations. Thus, the chemical-epigenetic method has been considered a powerful approach for new bioactive natural product discovery from fungi [14,16,17,18,19,154,155,156].

In addition to the frequently used chemical epigenetic modifiers mentioned in the review, many natural products have been screened to show chemical epigenetic regulating activities. They are an important source for chemical epigenetic modifiers applied in fungal secondary metabolisms [20,21].

Chemical epigenetic and molecular epigenetic modifications are two strategies used to convert a heterochromatic structure to euchromatin in order to induce the expression of biosynthetic gene clusters for the secondary metabolism [157]. If a certain type of chemical epigenetic modifier, such as histone deacetylase inhibitors, was found to be very effective for secondary metabolism to a certain fungus, it may guide us to either knock out or overexpress histone acetyltransferase genes in order to activate the production of fungal secondary metabolites.

The following aspects should be focused on in future research. (1) More natural products should be screened as soon as possible for their chemical epigenetic regulating function in fungal secondary metabolisms. (2) The number of fungal species treated with epigenetic modifiers needs to be increased. There is a great potential to identify new bioactive natural products from fungi. (3) Some chemical modifiers usually lead to the incremental changes in secondary metabolite contents, while others usually stimulate production of the novel compounds. Some chemical modifies may have other functions on fungal cells besides their epigenetic regulation function in fungal secondary metabolism. For examples, GlcNAc was considered as the DNA methyltransferase modifier [25]. It also regulated the expression of many virulence genes of pathogens to provide a survival advantage to the pathogens in the host [158]. Nicotinamide was an inhibitor of NAD^+^-dependent HDAC of class III in epigenetic regulation of fungal secondary metabolism [47]. Addition of nicotinamide in the medium, the production of fungal secondary metabolites was often promoted. In addition, nicotinamide enhanced the antifungal activities of amphotericin B against *Candida albicans Cryptococcus neoformans*. It also enhanced anti-biofilm activity of amphotericin B [159]. Some chemicals such as metal ions [160,161] and two-phse solvents [162,163] could enhanced production of fungal secondary metabolties. These chemicals might not be acted as the epigenetic modifiers to affect production of fungal secondary metabolites. So the action mechanisms of chemicals on fungal secondary metabolism are very complicatated, which should be studied in detail. (4) With the popularity of fungal genome sequencing technology, we can easily realize the gene clusters of secondary metabolite biosynthesis by coupling with the bioinformatics prediction. Thus, epigenetic regulations to activate cryptic biosynthetic gene clusters of secondary metabolism should be easily revealed. (5) Epigenetic engineering of secondary metabolisms based on epigenetic regulation is emerging as a powerful strategy for the management of either mycotoxin-producing fungi or plant pathogenic fungi that synthesize phytotoxins.

## Data Availability

Not applicable.

## Supplementary Materials

The following are available online at https://www.mdpi.com/article/10.3390/jof9020172/s1, Figure S1: Structures of the compounds **26**–**132** isolated from fungi treated with 5-azacydidine; Figure S2: Structures of the compounds **133**–**138** isolated from fungi treated with other DNA methyltransferase modifiers; Figure S3: Structures of the compounds **139**–**149** isolated from fungi treated with two DNA methyltransferase modifiers; Figure S4: Structures of the compounds **150**–**290** isolated from fungi treated with suberoylanilide hydroxamic acid; Figure S5: Structures of the compounds **291**–**351** isolated from fungi treated with suberoylbishydroxamic acid; Figure S6: Structures of the compounds **352**–**371** isolated from fungi treated with valproic acid or sodium valproate; Figure S7: Structures of the compounds **372**–**410** isolated from fungi treated with sodium butyrate; Figure S8: Structures of the compounds **411**–**439** isolated from fungi treated with nicotinamide; Figure S9: Structures of the compounds **440**–**451** isolated from fungi treated with trichostatin A; Figure S10: Structures of the compounds **452**–**462** isolated from fungi treated with other histone deacetylase modifiers; Figure S11: Structures of the compounds **463** and **464** isolated from fungi treated with two histone deacetylase modifiers; Figure S12: Structures of the compounds **465**–**467** isolated from fungi treated with histone acetyltransferase modifier anacardic acid; Figure S13: Structures of the compounds **468**–**470** isolated from fungi treated with histone methyltransferase modifier BRD4770; Figure S14: Structures of the compounds **471**–**475** isolated from fungi treated with proteasome modifier bortezomib; Figure S15: Structures of the compounds **476**–**486** isolated from fungi treated with NPD938; Figure S16: Structures of the compounds **487**–**570** isolated from fungi treated with two types of chemical epigenetic modifiers; Table S1: The examples of 5-Aza affecting production of fungal secondary metabolites; Table S2: The examples of SAHA affecting production of fungal secondary metabolites; Table S3: The examples of SBHA affecting production of fungal secondary metabolites; Table S4: The examples of VPA or SVP affecting production of fungal secondary metabolites; Table S5: The examples of NaBut affecting production of fungal secondary metabolites; Table S6: The examples of nicotinamide affecting production of fungal secondary metabolites; Table S7: The examples of other histone deacetylase modifiers affecting production of fungal secondary metabolites; Table S8: The examples of two types of chemical epigenetic modifiers affecting production of fungal secondary metabolites. All the references cited in the supplementary tables are listed in the section References of the text.
