# Supplementary material for: Recent Advances in Search of Bioactive Secondary Metabolites from Fungi Triggered by Chemical Epigenetic Modifiers
Source: J Fungi (Basel). 2023 Jan 28;9(2):172. doi: 10.3390/jof9020172 (PMC9961798; doi:10.3390/jof9020172)
Supplement: Supplementary file 1 [file jof-09-00172-s001.zip › jof-2083601-supplementary.pdf]

Supplementary Materials:

Recent Advances in Search of Bioactive Secondary Metabolites from Fungi Triggered by Chemical Epigenetic Modifiers

Mengyao Xue, Xuwen Hou, Jiajin Fu, Jiayin Zhang, Jiacheng Wang, Zhitong Zhao, Dan Xu, Daowan Lai, and Ligang Zhou *

State Key Laboratory of Agrobiotechnology, Department of Plant Pathology, College of Plant Protection, China Agricultural University, Beijing 100193, China
* Correspondence: lgzhou@cau.edu.cn; Tel.: +86-10-6273-1199

Contents

Figure S1. Structures of the compounds **26**–**132** isolated from fungi treated with 5-azacydidine (5-Aza). 7

Figure S2. Structures of the compounds **133**–**138** isolated from fungi treated with other DNA methyltransferase modifiers. 8

Figure S3. Structures of the compounds **139**–**149** isolated from fungi treated with two DNA methyltransferase modifiers. 8

Figure S4. Structures of the compounds **150**–**290** isolated from fungi treated with suberoylanilide hydroxamic acid (SAHA). 14

Figure S5. Structures of the compounds **291**–**351** isolated from fungi treated with suberoylbishydroxamic acid (SBHA)........ 17

Figure S6. Structures of the compounds **352**–**371** isolated from fungi treated with valproic acid (VAP) or sodium valproate (SVP). 18

Figure S7. Structures of the compounds **372**–**410** isolated from fungi treated with sodium butyrate (NaBut). 20

Figure S8. Structures of the compounds **411**–**439** isolated from fungi treated with nicotinamide. 22

Figure S9. Structures of the compounds **440**–**451** isolated from fungi treated with trichostatin A (TSA)........ 23

Figure S10. Structures of the compounds **452**–**462** isolated from fungi treated with other histone deacetylase modifiers. 24

Figure S11. Structures of the compounds **463** and **464** isolated from fungi treated with two histone deacetylase modifiers. 24

Figure S12. Structures of the compounds **465**–**467** isolated from fungi treated with histone acetyltransferase modifier anacardic acid........ 25

Figure S13. Structures of the compounds **468**–**470** isolated from fungi treated with histone methyltransferase modifier BRD4770. 25

Figure S14. Structures of the compounds **471–475** isolated from fungi treated with proteasome modifier bortezomib.. 25

Figure S15. Structures of the compounds **476–486** isolated from fungi treated with NPD938. 26

Figure S16. Structures of the compounds **487–570** isolated from fungi treated with two types of chemical epigenetic modifiers. 30

Table S1. The examples of 5-Aza affecting production of fungal secondary metabolites. 31

Table S2. The examples of SAHA affecting production of fungal secondary metabolites................................ 33

Table S3. The examples of SBHA affecting production of fungal secondary metabolites................. 35

Table S4. The examples of VPA or SVP affecting production of fungal secondary metabolites....................... 36

Table S5. The examples of NaBut affecting production of fungal secondary metabolites. 37

Table S6. The examples of nicotinamide affecting production of fungal secondary metabolites. 38

Table S7. The examples of other histone deacetylase modifiers affecting production of fungal secondary metabolites. 39

Table S8. The examples of two types of chemical epigenetic modifiers affecting production of fungal secondary metabolites.. 40

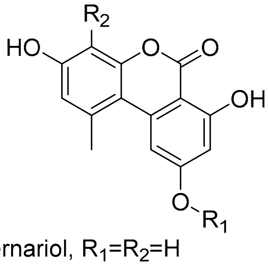

26. Alternariol, R_1=R_2=H
27. Alternariol-5-*O*-methyl ether, R_1=CH_3, R_2=H
28. 3'-Hydroxyalternariol-5-*O*-methyl ether, R_1=CH_3, R_2=OH

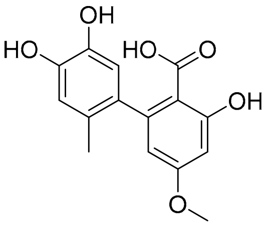

29. Altenusin

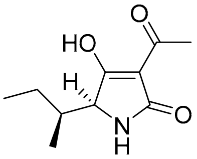

30. Tenuazonic acid

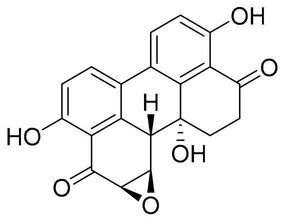

31. Altertoxin II

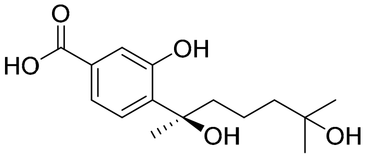

32. (7*R*)-Hydroxysydonic acid

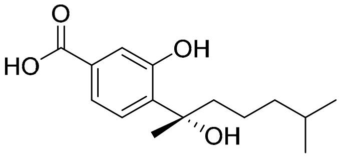

33. (7*S*)-Sydonic acid

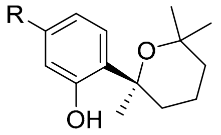

34. (*S*)-5-(Hydroxymethyl)-2-(2',6',6'-trimethyltetrahydro-2*H*-pyran-2-yl) pheol, R=CH_2OH
37. (*S*)-Sydowic acid, R=COOH

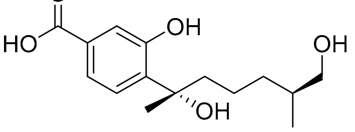

35. (7*S*,11*S*)-Hydroxysydonic acid

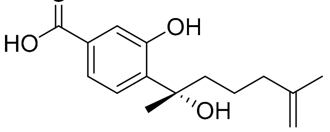

36. (7*S*)-11-Dehydrosydonic acid

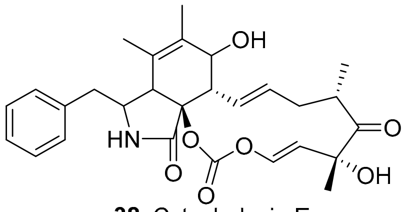

38. Cytochalasin E

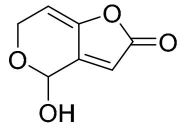

39. Patulin

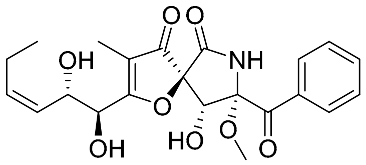

40. Pseurotin A

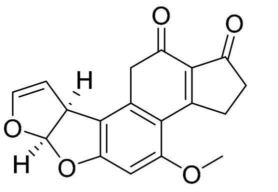

41. Aflatoxin B1

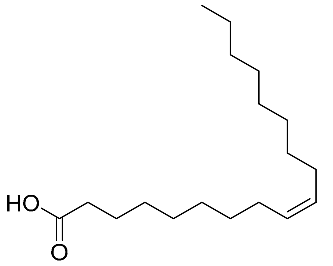

42. (*Z*)-9-Octadecenoic acid

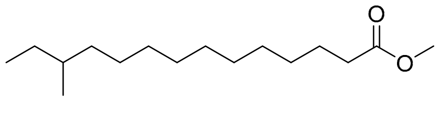

43. 12-Methyl-tetradecanoic acid methylester

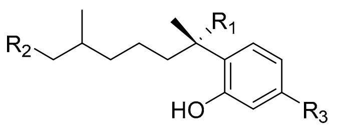

44. (7*S*)-7-*O*-Methylsydonol, R_1=OCH_3, R_2=H, R_3=CH_2OH
45. (7*S*,11*S*)-12-Hydroxysydonic acid, R_1=OH, R_2=OH, R_3=COOH
47. (*S*)-Sydonol, R_1=OH, R_2=H, R_3=CH_2OH

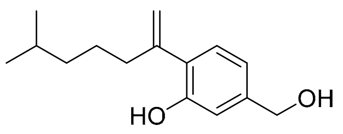

46. 7-Deoxy-7,14-didehydrosydonol

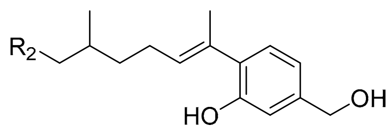

48. Anhydrowaraterpol B, R=OH
49. (*E*)-5-Hydroxymethyl-2-(6'-methyl hept-2'-en-2'-yl)phenol, R=H

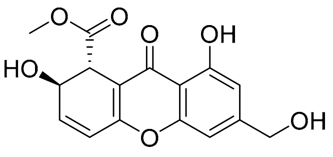

50. AGI-B4

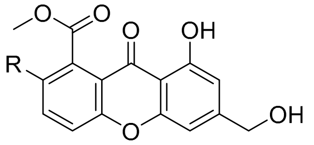

51. Sydowinin A, R=H
52. Sydowinin B, R=OH

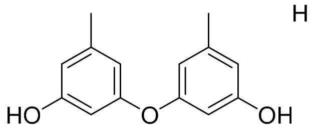

53. Diorcinol

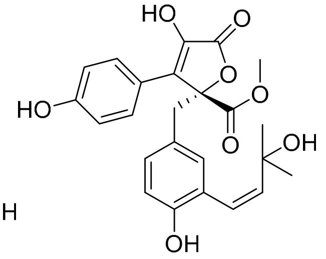

54. Asperbutyrolactone A

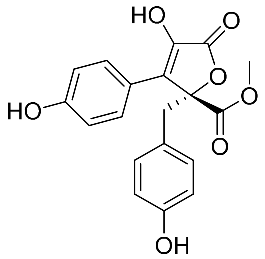

55. Asperbutyrolactone B

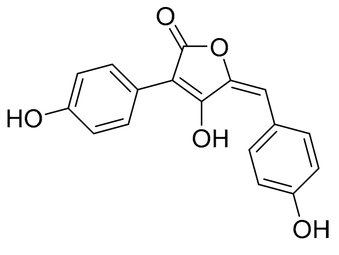

56. Aspulvinone E

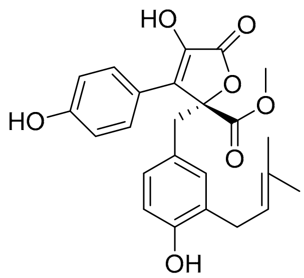

57. Butyrolactone I

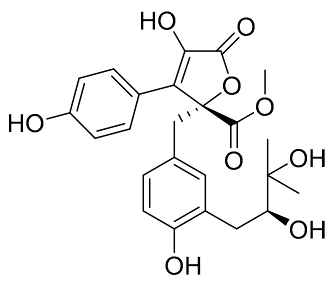

58. Butyrolactone VI

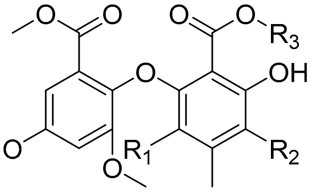

59. Asterric acid, R_1=H, R_2=H, R_3=H
60. Penicillither, R_1=Cl, R_2=H, R_3=CH_3
61. Methyl asterrate, R_1=H, R_2=H, R_3=CH_3
62. 4',6',-Dichloroasterric acid, R_1=Cl, R_2=Cl, R_3=CH_3

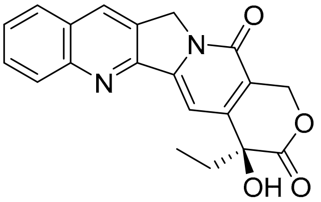

63. Camptothecine

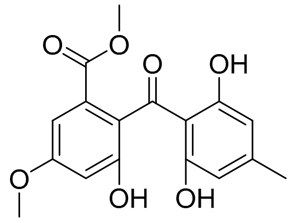

64. Isosulochrin

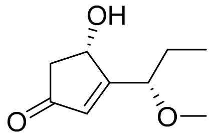

65. Globosporin A

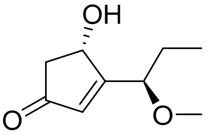

66. Globosporin B

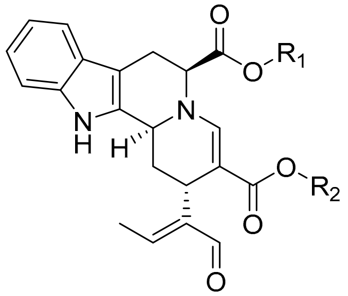

67. Globosporine C, R_1=CH_3, R_2=H
69. Mappianine E, R_1=H, R_2=CH_3

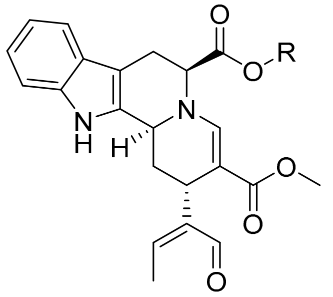

68. Globosporine D, R=H
70. 19(20)*Z*-5-Carboxymethylvallesiachotamine, R=CH_3

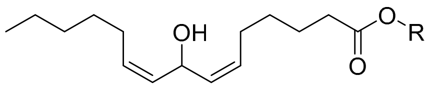

71. (9*Z*,12*Z*)-11-Hydroxyoctadeca-9,12-dienoic acid, R=H
72. (9*Z*,12*Z*)-11-Hydroxyoctadeca-9,12-dienoic acid, methyl ester, R=CH_3
73. (9*Z*,12*Z*)-11-Hydroxyoctadeca-9,12-dienoic acid glycerol conjugate, R=CH_2-CHOH-CH_2OH

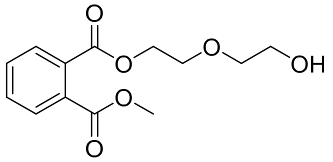

74. Cochphthester A

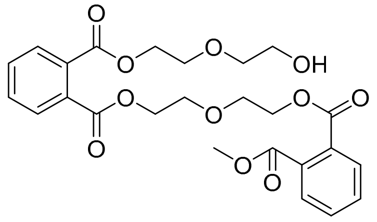

75. Cochphthester B

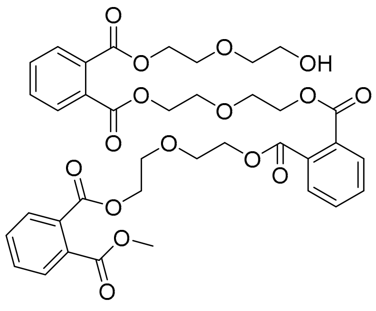

76. Cochphthester C

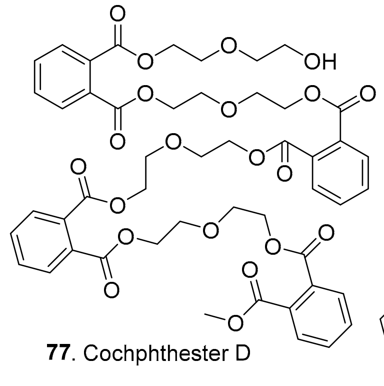

77. Cochphthester D

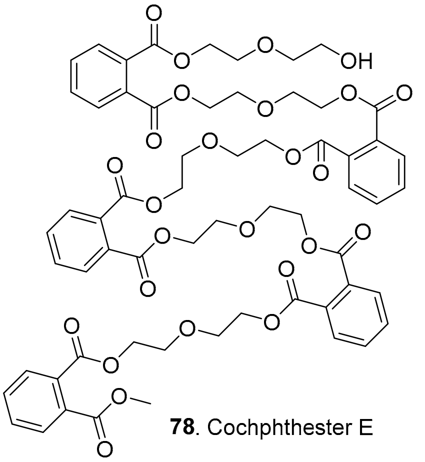

78. Cochphthester E

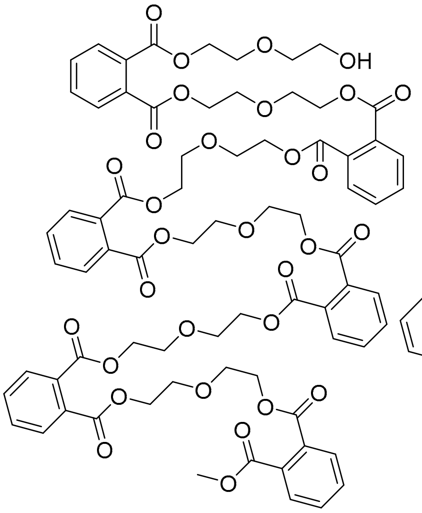

79. Cochphthester F

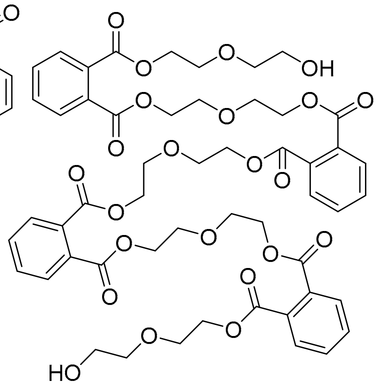

80. Cochphthester G

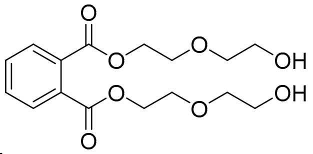

81. 1,2-Benzenedicarboxylic acid,1,2-bis [2-(2-hydroxyethoxy) ethyl] ester

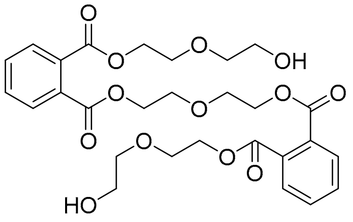

82 1,2-Benzenedicarboxylic acid,1,1-(oxydi-2,1-ethanediyl) 2,2 -bis [2-(2-hydroxyethoxy) ethyl] ester

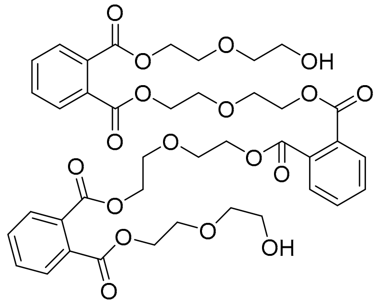

83. 1,2-Benzenedicarboxylic acid, 1,2-bis[2-[[2-[[2-(2-hydroxyethoxy) ethoxy] carbonyl] oxy] ethoxy] ethyl] ester

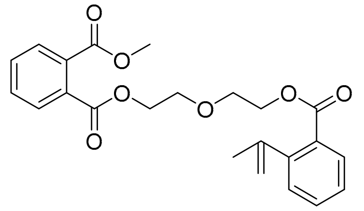

84. 1,2-Benzenedicarboxylic acid, oxy-di-2,1-ethanediyl dimethylester

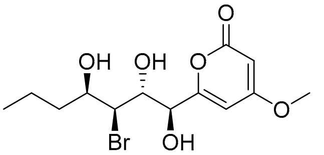

85. Cochliobopyrone A

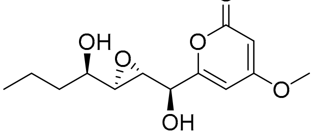

86. Cochliobopyrone B

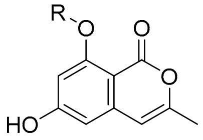

87. 3-Methyl-6,8-dihydroxyisocoumarin, R=CH_3

88. 6-Hydroxy-8-methoxy-3-methyl-isocoumarin, R=H

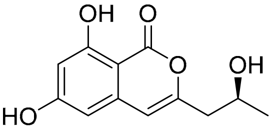

89. (*S*)-Orthosporin

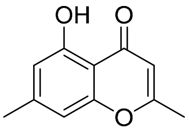

90. Altechromone A

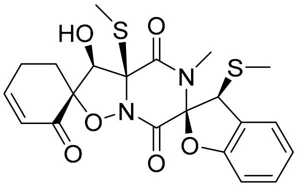

91. Botryosulfuranol A

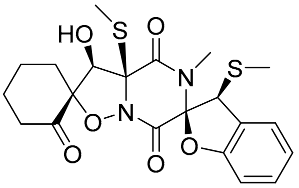

92. Botryosulfuranol B

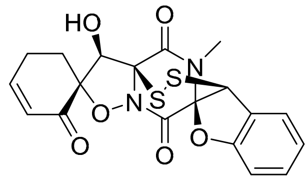

93. Botryosulfuranol C

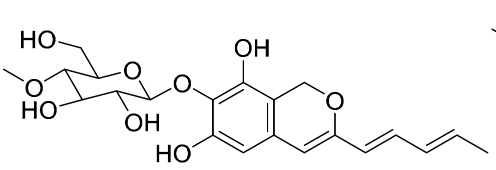

94. Indigotide A

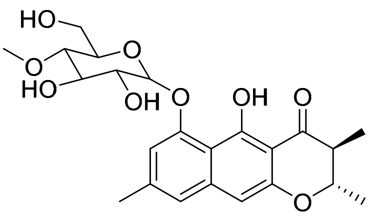

95. Indigotide B

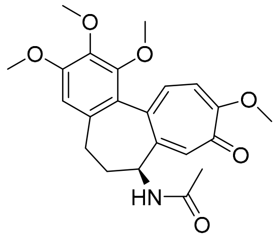

96. Colchicine

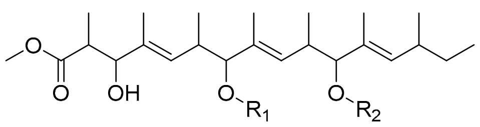

97. Lunalide A, R_1=H, R_2=β-D-Mannopyranoside
98. Lunalide B, R_1=R_2=β-D-Mannopyranoside

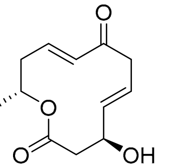

99. Dendrodolide E

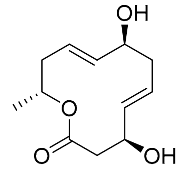

100. Dendrodolide G

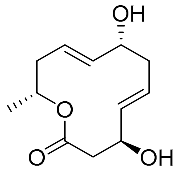

101. Dendrodolide I

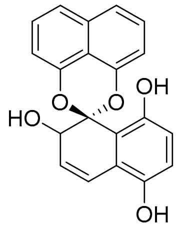

102. Palmarumycin C30

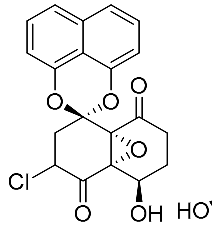

103. Palmarumycin C8

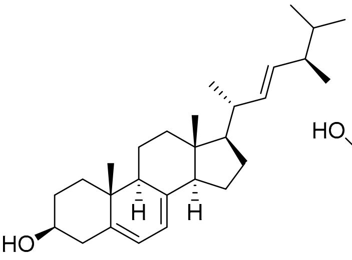

104. Ergosterol

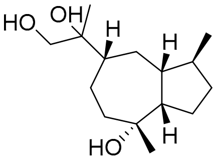

105. Xylaguaianol C

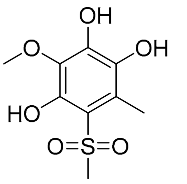

106. **3**-Methoxy-6-methyl-5-(methylsulfonyl) benzene-1,2,4-triol

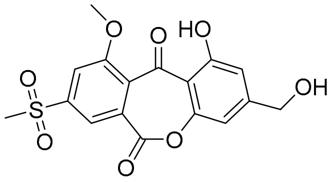

107. Neosartoryone A

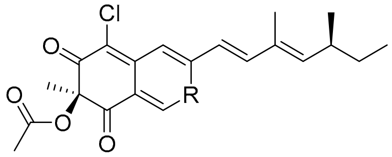

108. Sclerotiorin, R=O
113. Sclerotioramine, R=NH

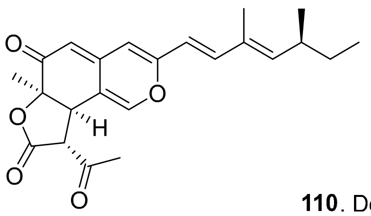

109. Ochrephilone

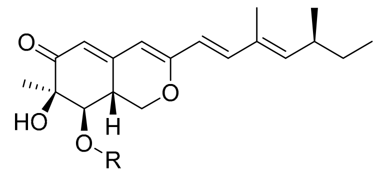

110. Dechloroisochromophilone III, R=H
111. Dechloroisochromophilone IV, R=Ac

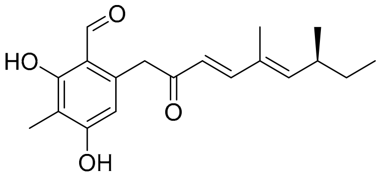

112. 6-((3E,5E)-5,7-Dimethyl-2-methylenenona-3,5-dienyl)-2,4-dihydroxy-3-methylbenzaldehyde

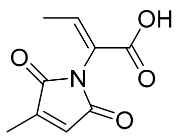

114. Pencolide

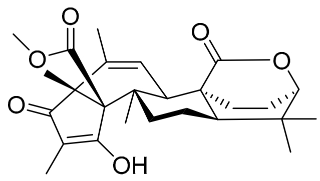

115. Atlantinone A

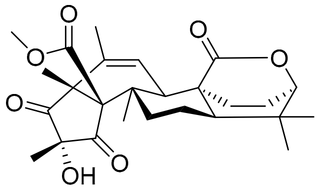

116. Atlantinone B

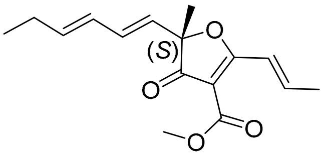

120. Aspertetronin A

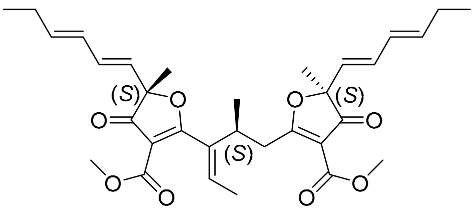

117. Miniolin A

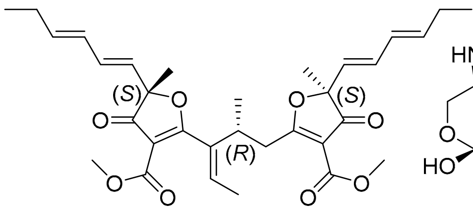

118. Miniolin B

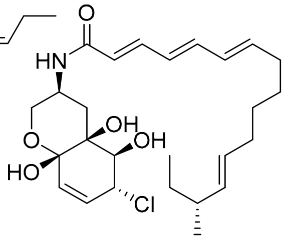

121. Varitatin A

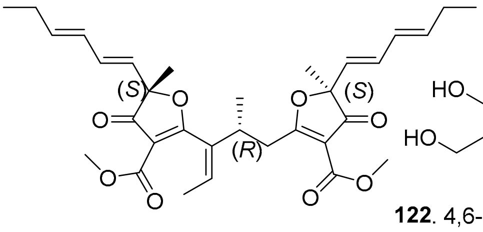

119. Miniolin C

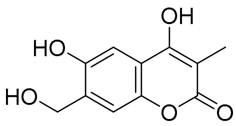

122. 4,6-Dihydroxy-3,7-dimethylcoumarin

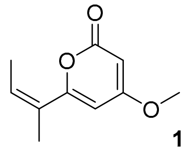

123. Pestalotio-pyrone G

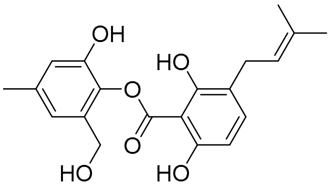

124. 2'-Hydroxy 6'-hydroxymethyl-4'-methylphenyl-2,6-dihydroxy-3-(2-isopentenyl)benzoate

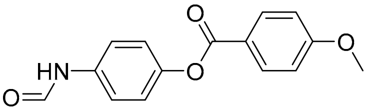

125. 4'-Formamidophenyl-5-methoxybenzoate

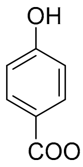

126. 4-Hydroxybenzoic acid

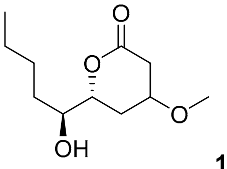

127. LL-P880α

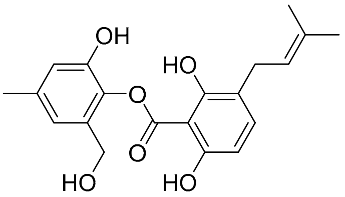

128. 2'-Hydroxy 6'-hydroxymethyl-4'-methylphenyl-2,6-dihydroxy-3-(2-isopentenyl)benzoate

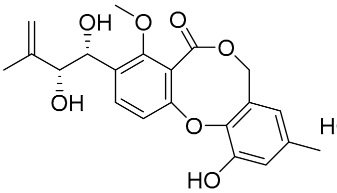

129. Pestalotiollide B

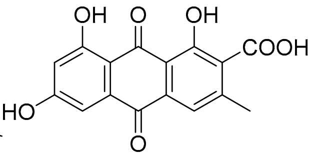

130. Endocrocin

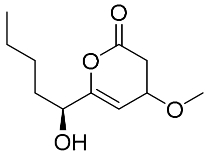

131 1'-Hydroxy-4-methoxy-6-pentyl-2*H*-pyran-2-one

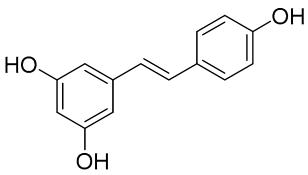

132. Resveratrol

Figure S1. Structures of the compounds **26–132** isolated from fungi treated with 5-azacydidine (5-Aza).

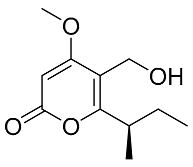

133. (*S*)-6-(Sec-butyl)-5-(hydroxymethyl)-4-methoxy-2*H*-pyran-2-one

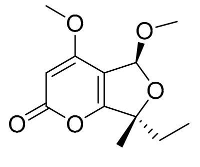

134. (5*S*,7*R*)-7-Ethyl-4,5-dimethoxy-7-methyl-5,7-dihydro-2*H*-furo[3,4-b]pyran-2-one

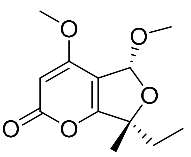

135. (5*R*,7*R*)-7-Ethyl-4,5-dimethoxy-7-methyl-5,7-dihydro-2*H*-furo[3,4-b]pyran-2-one

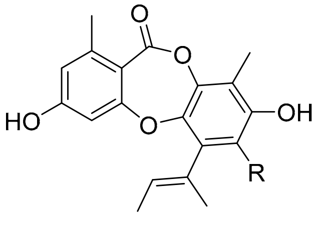

136. Aspergillusidone F, R=Br
137. Unguinol, R=H

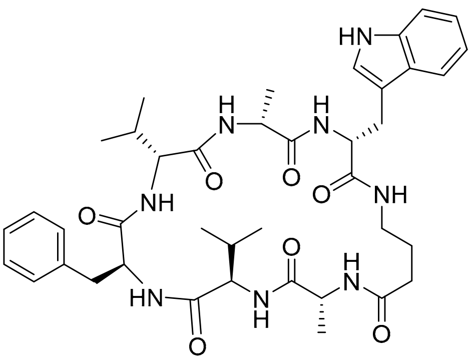

138. Unguisin A

Figure S2. Structures of the compounds **133–138** isolated from fungi treated with other DNA methyltransferase modifiers.

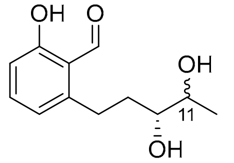

139. Pestalotiopol A, 11*S*
144. Heterocornol A, 11*R*

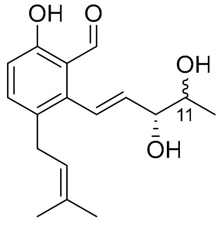

140. Pestalotiopol B, 11*S*
145. Heterocornol E, 11*R*

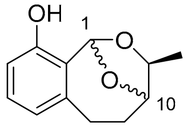

141. Pestalotiopol C, 1*R*, 10*S*
146. Heterocornol F, 1*S*, 10*R*

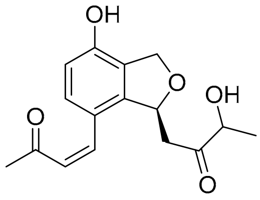

142. Pestalotiopol D

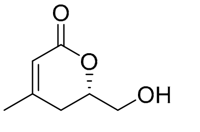

143. (*S*)-6-(Hydroxymethyl)-4-methyl-5,6-dihydro-2*H*-pyran-2-one

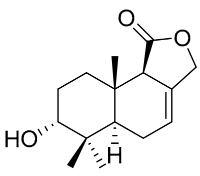

147. Pestalotiopol B

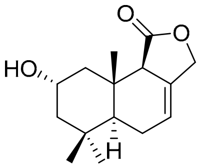

148. Dendocarbin B

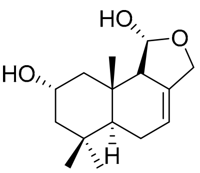

149. 2α-Hydrxoysisodrimeninol

Figure S3. Structures of the compounds **139–149** isolated from fungi treated with two DNA methyltransferase modifiers.

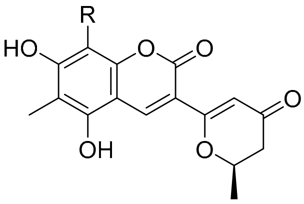

150. Aspyranochromenone A, R=OH
151. Aspyranochromenone B, R=H

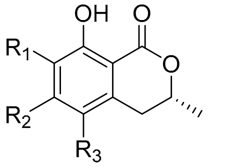

152. 6,7-Dihydroxymellin, R_1=OH, R_2=OH, R_3=H
154. 6-Hydroxymellein, R_1=H, R_2=OH, R_3=H
156. 6,7-Dimethoxymellein, R_1=OCH_3, R_2=OCH_3, R_3=H
157. 6-Methoxymellein, R_1=H, R_2=OCH_3, R_3=H
159. 5,6-Dihydroxymellein, R_1=H, R_2=OH, R_3=OH

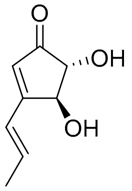

153. Terrein

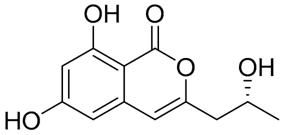

155. (*R*)-Orthosporin

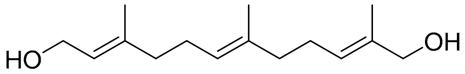

158. (2*E*,6*E*,10*E*)-12-Hydroxyfarnesol

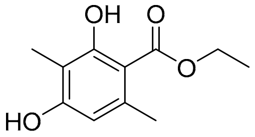

160. Ethyl 3-methylorsellinate

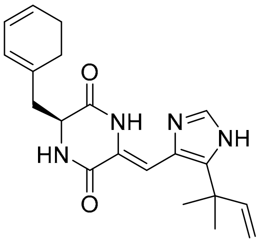

161. Phenylahistin

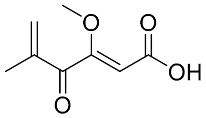

162. Penicillic acid

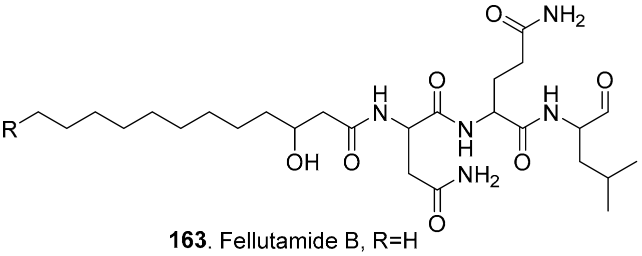

163. Fellutamide B, R=H
164. Antibiotic 15656G, R=CH_2CH_3

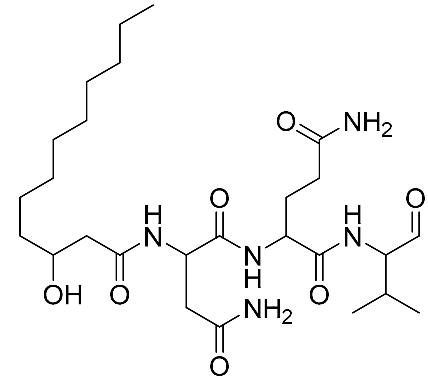

165. Antibiotic 3127

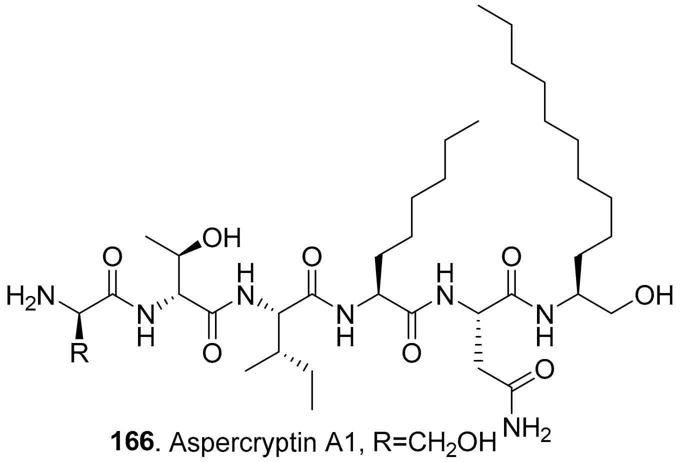

166. Aspercryptin A1, R=CH_2OH
167. Aspercryptin A2, R=CH_3

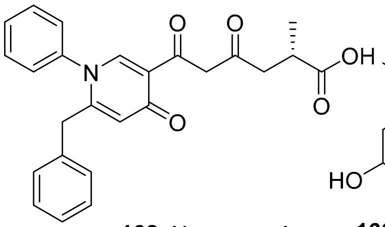

168. Nygerone A

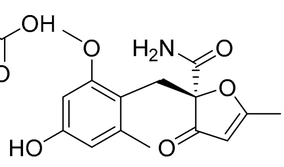

169. (+)-Asperfuranone

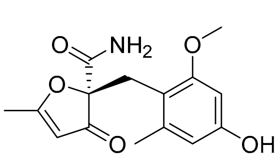

170. (-)-Asperfuranone

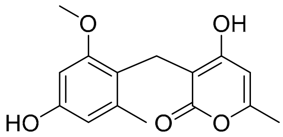

171. Asperpyranone A

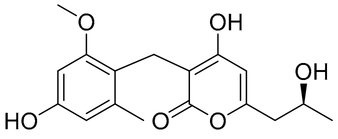

172. Asperpyranone B

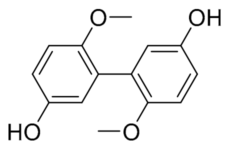

173. Versiperol A

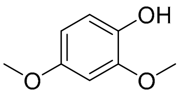

174. 2,4-Dimethoxyphenol

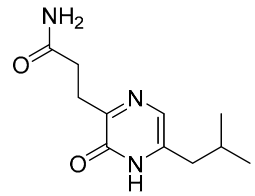

175. 3-[6-(2-Methylpropyl)-2-oxo-1*H*-pyrazin-3-yl] propanamide

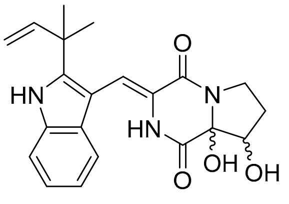

176. Brevianamide X

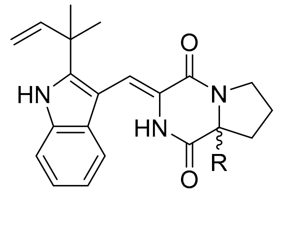

177. Brevianamide R, R=OCH_3
178. Brevianamide Q, R=OH

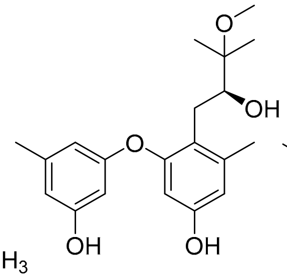

179. Diorcinol C

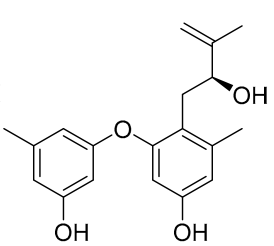

180. Diorcinol E

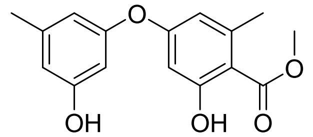

181. Diorcinol-4-carboxylate

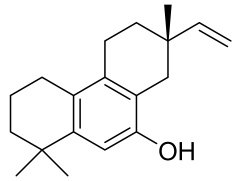

182. Aspewentin A

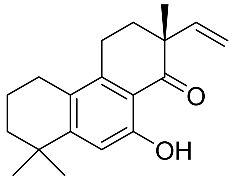

183. Aspewentin B

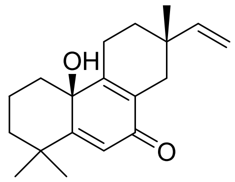

184. Aspewentin C

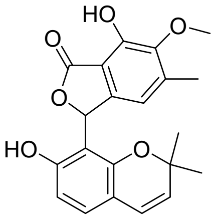

185. Primarolide A

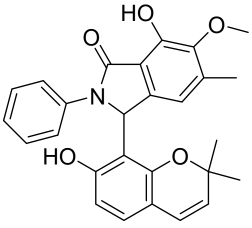

186. Primarolide B

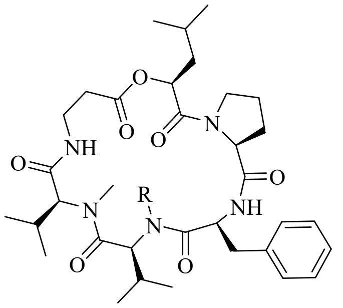

187. Desmethylisaridin E, R=H
188. Isaridin E, R=CH_3

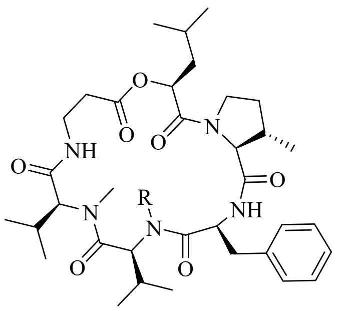

189. Desmethylisaridin C2, R=H
190. Isaridin C2, R=CH_3

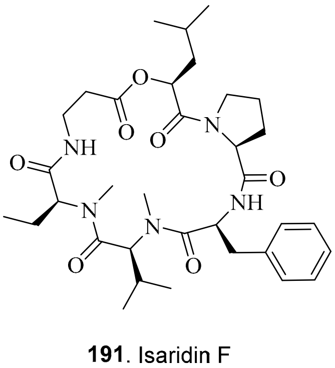

191. Isaridin F

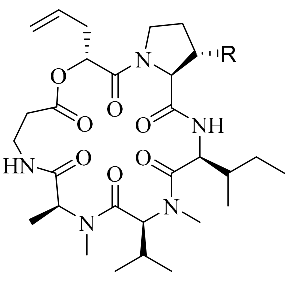

192. Destruxin A, R=H
193. Roseotoxin B, R=CH_3

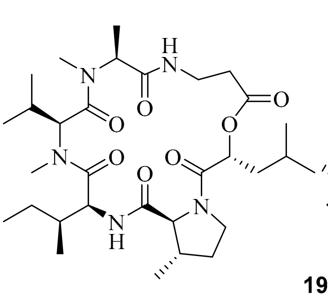

194. Roseocardin

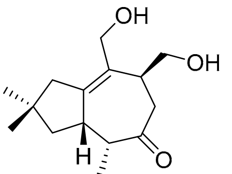

195. 11,12-Dihydroxy-1-tremulen-5-one

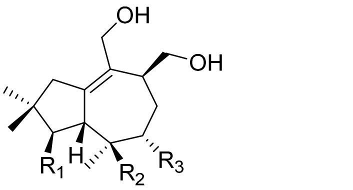

196. (3*S*,6*R*,7*R*)-Tremul-1-ene-6,11,12-triol, R_1=H, R_2=OH, R_3=H
197. Ceriponol A, R_1=OH, R_2=H, R_3=H
198. Conocenol B, R_1=H, R_2=H, R_3=OH

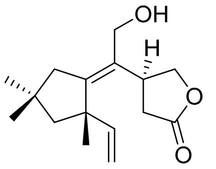

199. Conocenolide A

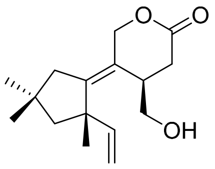

200. Conocenolide B

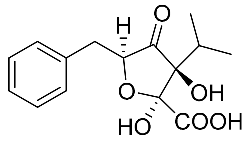

201. Botrycinereic acid

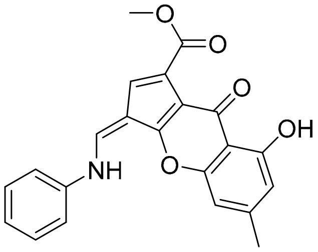

202. Chalaniline A

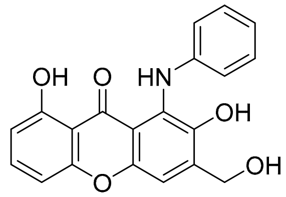

203. Chalaniline B

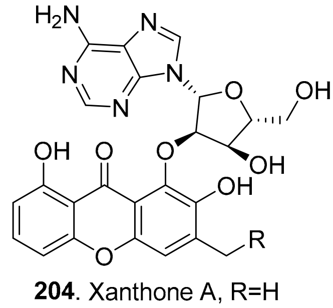

204. Xanthone A, R=H
205. Xanthone B, R=OH

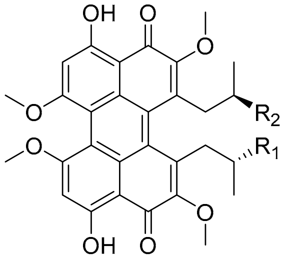

206. Cladochrome F, R_1=OH, R_2=ρ-Hydroxybenzoate
207. Cladochrome G, R_1=OH, R_2=ρ-Hydroxycarbonate
208. Cladochrome A, R_1=R_2=β-Hydroxycarbonate
209. Cladochrome B, R_1=β-Hydroxybutyrate, R_2=Benzoate
210. Cladochrome D, R_1=ρ-Hydroxybutyrate, R_2=ρ-Hydroxybenzoate
211. Cladochrome E, R_1=ρ-Hydroxycarbonae, R_2=Benzoate
212. Calphostin B, R_1=OH, R_2=Benzoate

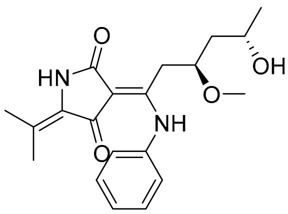

213. Cladosin H

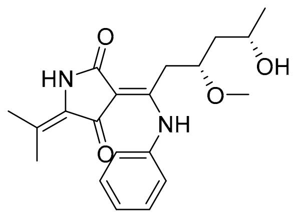

214. Cladosin I

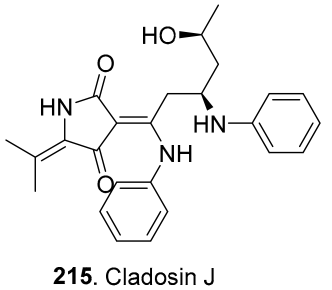

215. Cladosin J

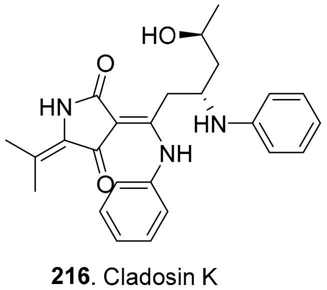

216. Cladosin K

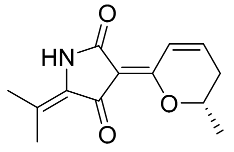

217. Cladodionen

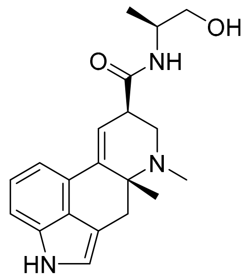

218. Ergometrine

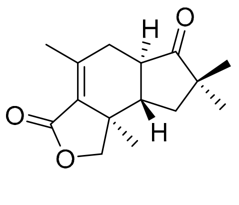

219. Cystercorolide

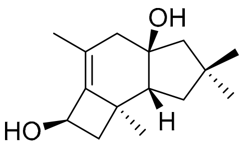

220. Cystercorodiol A

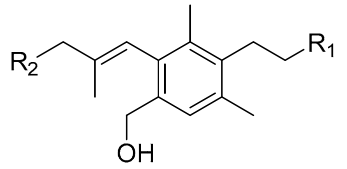

221. 4-*O*-Acetylcybrodol, R_1=OAc, R_2=OH
222. 14-Dehydroxybrodol, R_1=OH, R_2=H
231. Cybrodol, R_1=OH, R_2=OH

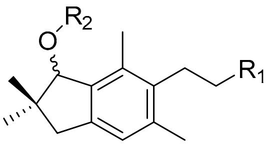

223. Cystercorodiol B, R_1=OH, R_2=H
224. 4-*O*-Acetylcystercorodiol B, R=OAc, R_2=H
225. 1-*O*-Methoxycystercorodiol B, R_1=OH, R_2=CH_3

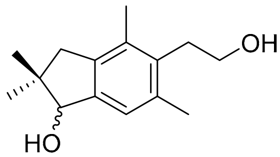

226. Cystercorodiol C

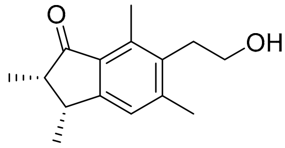

227. Cystercorotone

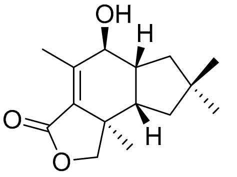

228. Epicoterpene D

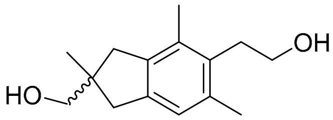

229. Russujaponol F

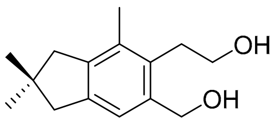

230. Riparol B

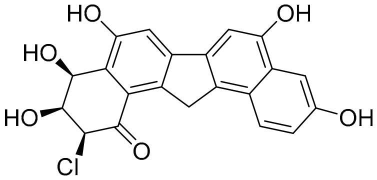

232. Daldinone E

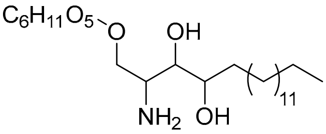

233. Hexosylphytosphyngosine

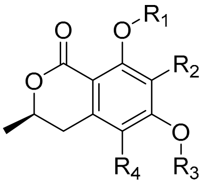

234. Palmaerone A, R_1=CH_3, R_2=H, R_3=H, R_4=H
235. Palmaerone B, R_1=CH_3, R_2=Br, R_3=H, R_4=H
236. Palmaerone C, R_1=CH_3, R_2=Br, R_3=CH_3, R_4=H
237. Palmaerone D, R_1=H, R_2=Br, R_3=H, R_4=H
238. Palmaerone E, R_1=CH_3, R_2=OH, R_3=H, R_4=Br
239. Palmaerone F, R_1=CH_3, R_2=H, R_3=H, R_4=Cl
240. Palmaerone G, R_1=CH_3, R_2=Cl, R_3=H, R_4=H
241. (*R*)-5-Cholro-6-hydroxymellein, R_1=H, R_2=H, R_3=H, R_4=Cl
243. Palmaerin A, R_1=CH_3, R_2=Cl, R_3=H, R_4=Cl
244. Palmaerin B, R_1=H, R_2=Br, R_3=H, R_4=Br
245. Palmaerin D, R_1=H, R_2=H, R_3=H, R_4=Br

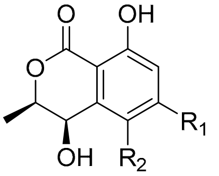

242. (3*R*,4*R*)-5-Cholro-4,6-dihydroxymellein, R_1=OH, R_2=Cl
247. *cis*-4-Hydroxymellein, R_1=H, R_2=H

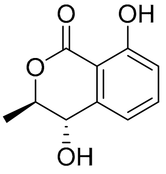

246. *trans*-4-Hydroxymellein

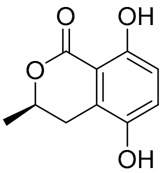

248. (*R*)-5-hydroxymellein

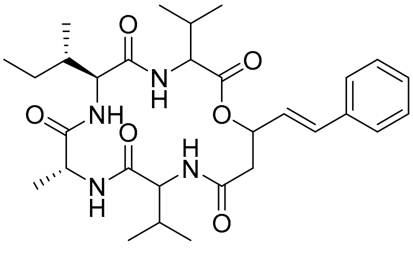

249. EGM-556

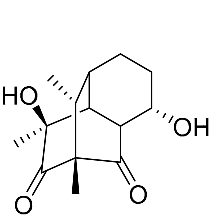

250. 4-Epipenicillone B

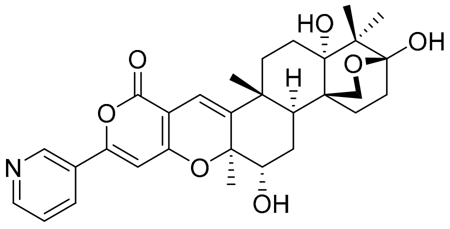

252. Pyrandecarurin A

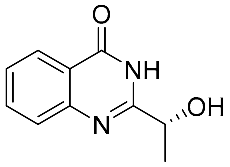

251. (*R*)--Chrysogine

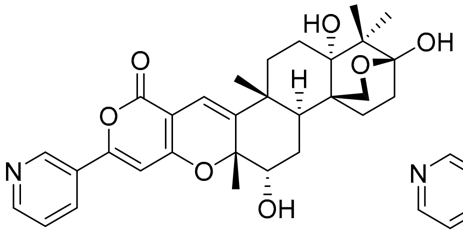

253. Pyrandecarurin B

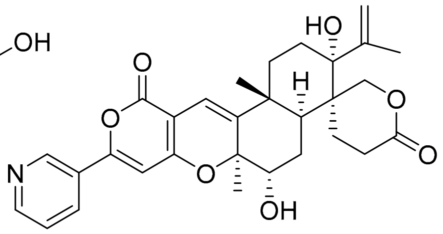

254. Pileotin A

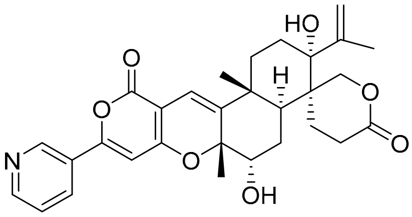

255. Pileotin B

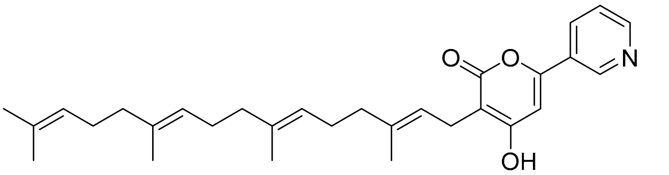

256. Decaturenoid

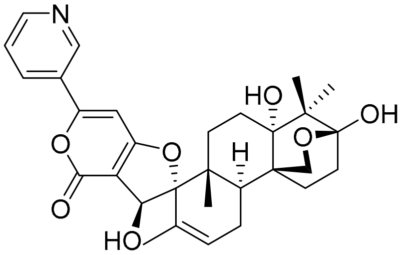

257. 15-Hydroxydecaturin A

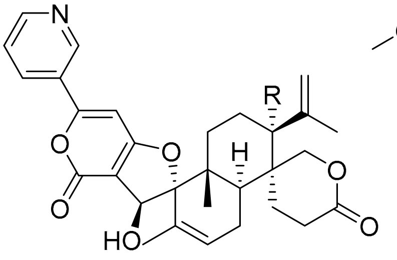

258. Oxalicine A, R=H
259. Oxalicine B, R=OH

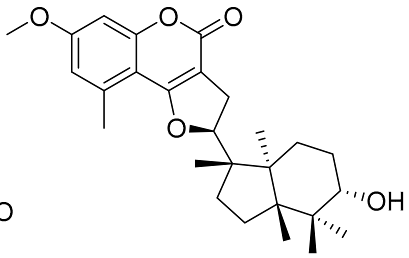

260. Penisarin B

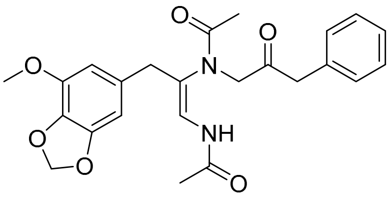

261. Brasiliamide A

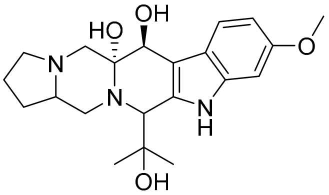

262. Verruculogen TR2

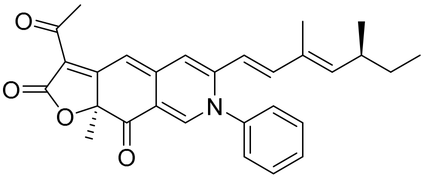

263. Isochromophilone XIV

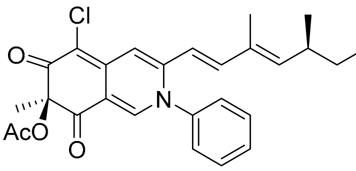

264. Isochromophilone XV

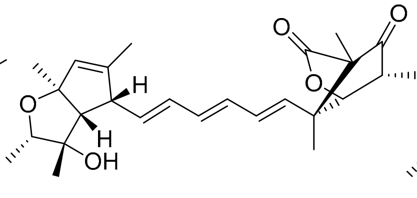

265. Varilactone A

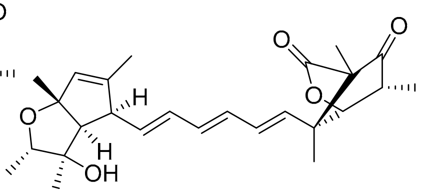

266. Varilactone B

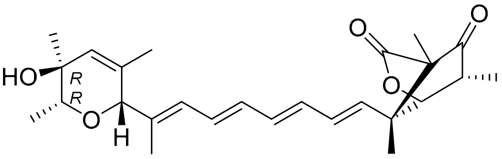

267. Wortmannilactone M

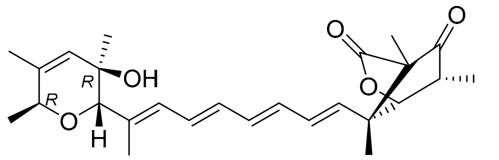

268. Wortmannilactone N

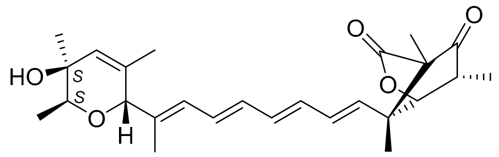

269. Wortmannilactone E

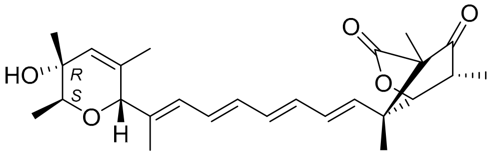

270. Wortmannilactone F

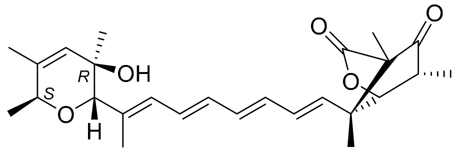

271. Wortmannilactone H

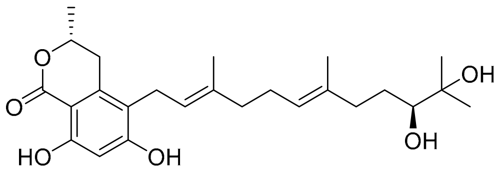

272. (10'*S*)-Verruculide B

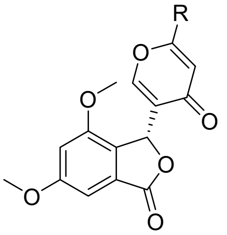

273. Vermistatin, R=CH=CHCH$_3$
274. Dihydrovermistatin, R=CH$_2$CH$_2$CH$_3$

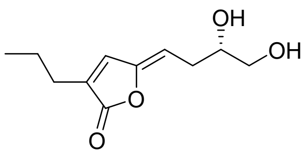

275. (*S*,*Z*)-5-(3',4'-Dihydroxy-butylidene)-3-propylfuran-2(5*H*)-one

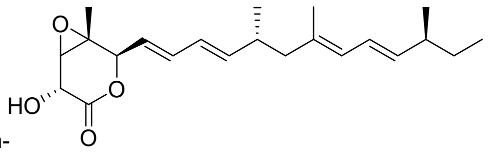

276. Nafuredin

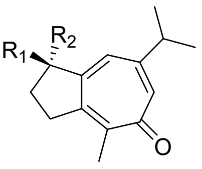

277. Spiromaterpene A, R$_1$=H, R$_2$=CH$_3$
278. Spiromaterpene B, R=CH$_3$, R$_2$=OH

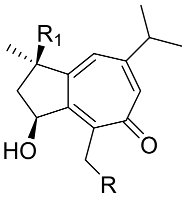

279. Spiromaterpene A, R$_1$=H, R$_2$=H
282. Spiromaterpene F, R$_1$=H, R$_2$=OH
285. Spiromaterpene I, R$_1$=OH, R$_2$=H

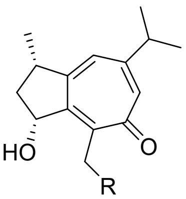

280. Spiromaterpene D, R=H
281. Spiromaterpene E, R=OH

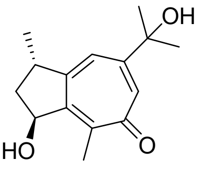

283. Spiromaterpene G

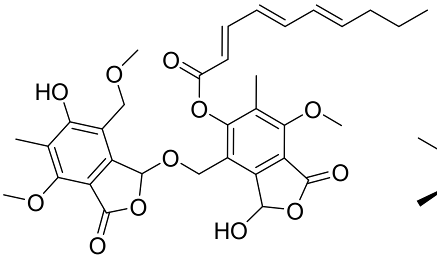

286. Funitatin A

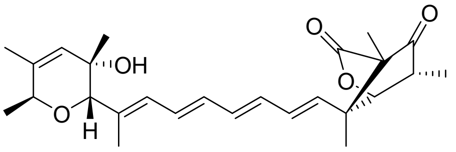

287. Wortmannilactone I

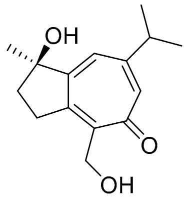

284. Spiromaterpene H

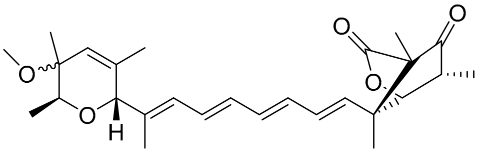

288. Wortmannilactone J, 19*S*
289. Wortmannilactone K, 19*R*

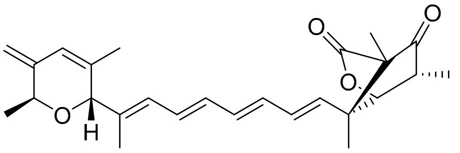

290. Wortmannilactone L

Figure S4. Structures of the compounds **150–290** isolated from fungi treated with suberoylanilide hydroxamic acid (SAHA).

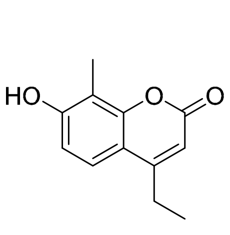

291. 4-Ethyl-7-hydroxy-8-methyl-2*H*-1-benzopyran-2-one

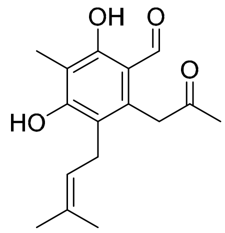

292. Chaetophenol A

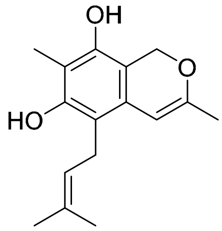

293. Chaetophenol B

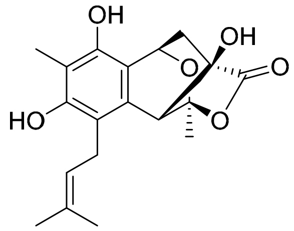

294. Chaetophenol C

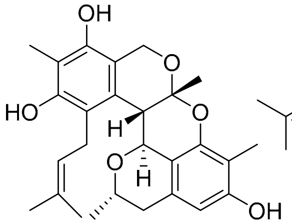

295. Chaetophenol D

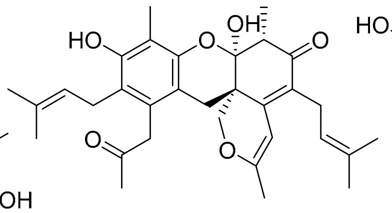

296. Chaetophenol E

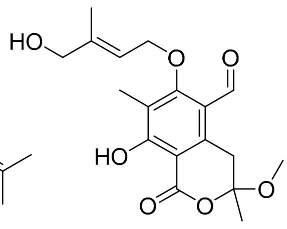

297. Chaetophenol F

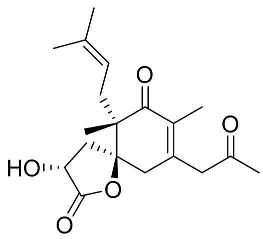

298. Spiroindicumide A

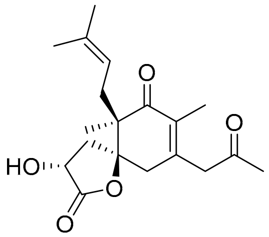

299. Spiroindicumide B

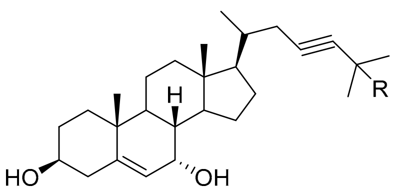

300. (3β,7α)-Cholest-5-en-23-yne-3,7-diol, R=H
301. (3β,7α)-Cholest-5-en-23-yne-3,7,25-triol, R=OH

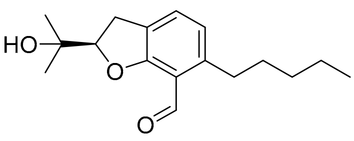

302. Annullatin A

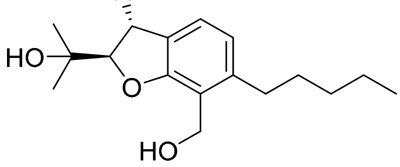

303. Annullatin B, R=H
304. Annullatin C, R=OH

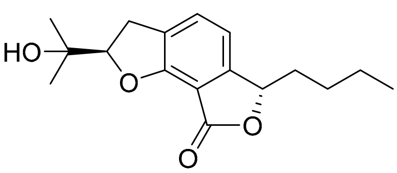

305. Annullatin D

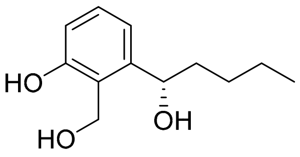

306. Annullatin E

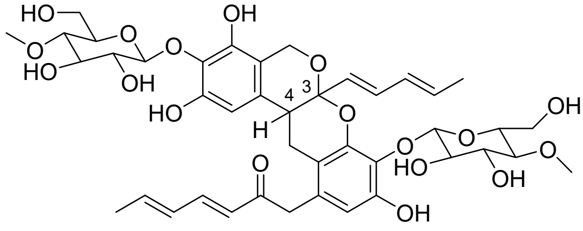

307. Indigotide C, 3*R*, 4*R*
308. Indigotide D, 3*S*, 4*S*

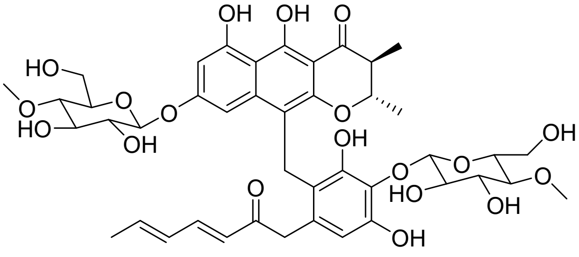

309. Indigolide E

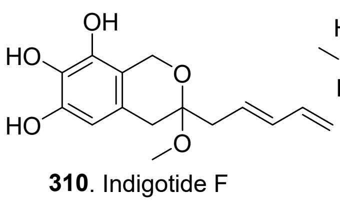

310. Indigotide F

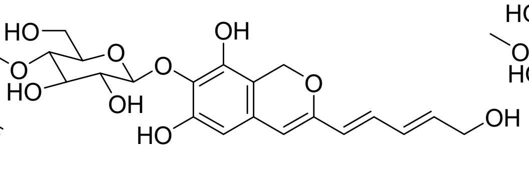

311. 13-Hydroxyindigotide A

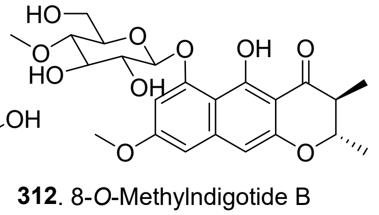

312. 8-*O*-Methylndigotide B

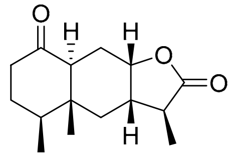

313. Eutyperemophilane A

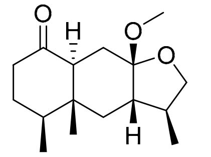

314. Eutyperemophilane B

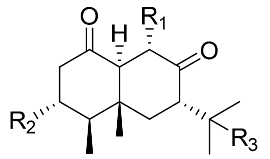

315. Eutyperemophilane C, R_1=R_2=R_3=H
316. Eutyperemophilane D, R_1=R_2=H, R_3=OH
317. Eutyperemophilane E, R_1=R_3=H, R_2=OH
318. Eutyperemophilane F, R_1=OH, R_2=R_3=H

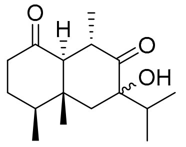

319. Eutyperemophilane G,·····OH
320. Eutyperemophilane H, ━OH

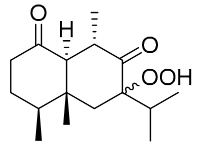

321. Eutyperemophilane I,····· OOH
322. Eutyperemophilane J,━ OOH

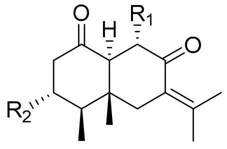

323. Eutyperemophilane K, R_1=R_2=H
324. Eutyperemophilane L, R_1=OH, R_2=H
325. Eutyperemophilane M, R_1=H, R_2=OH

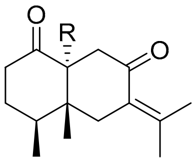

326. Eutyperemophilane N, R=H
327. Eutyperemophilane O, R=OH

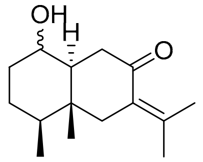

328. Eutyperemophilane P,━ OH
329. Eutyperemophilane Q,·····OH

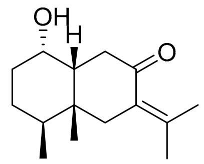

330. Eutyperemophilane R

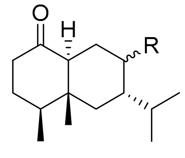

331. Eutyperemophilane S,━ OH
332. Eutyperemophilane T,·····OH

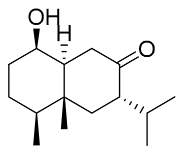

333. Eutyperemophilane U

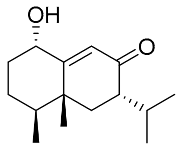

334. Eutyperemophilane V

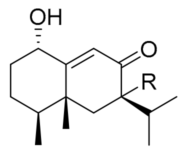

335. Eutyperemophilane W, R=H
336. Eutyperemophilane X, R=OH

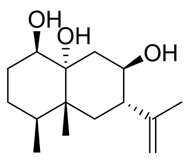

337. Eutyperemophilane Y

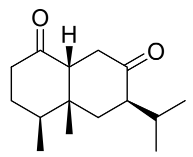

338. Eutyperemophilane Z

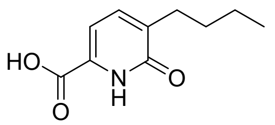

339. 5-Butyl-6-oxo-1,6-dihydropyridine-2-carboxylic acid

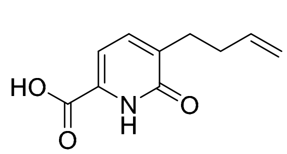

340. 5-(But-9-enyl)-6-oxo-1,6-dihydropyridine-2-carboxylic acid.

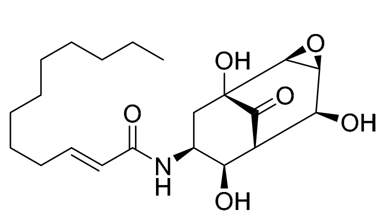

341. Isariotin A

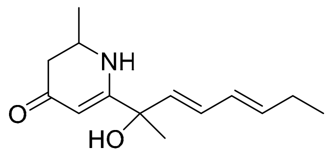

342. JBIR-54

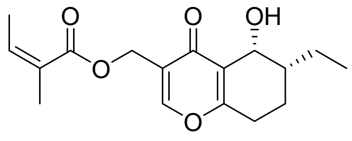

343. 13-Angeloyloxy-diplosporin

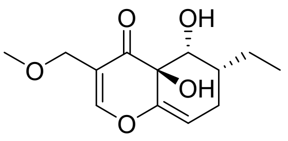

344. 3-Methoxymethyl agistatine

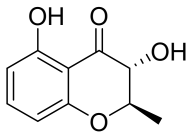

345. Gynuraone

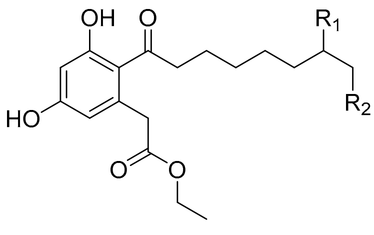

346. Cytosporone B, R_1=H, R_2=H
347. Dothiorelone A, R_1=OH, R_2=H
348. Dothiorelone C, R_1=H, R_2=OH

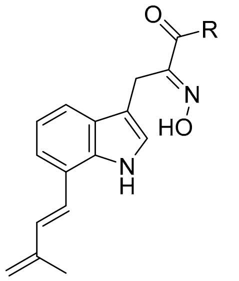

349. Luteoride A, R=OCH_3
350. Luteoride B, R=NH_2

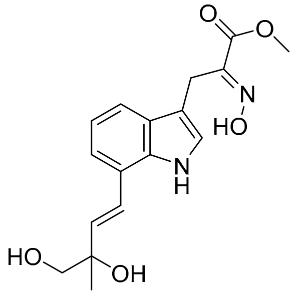

351. Luteoride C

Figure S5. Structures of the compounds **291–351** isolated from fungi treated with suberoylbishydroxamic acid (SBHA).

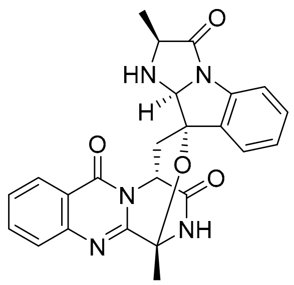

352. Fumiquinazoline C

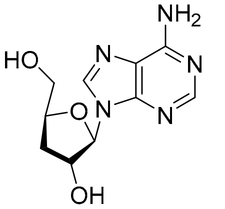

353. Cordycepin

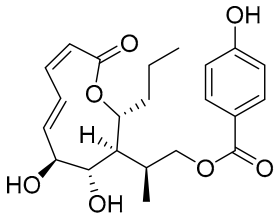

354. Xylarolide A

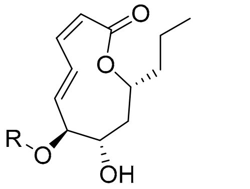

355. Xylarolide B, R=CH_3
357. Xylarolide, R=H

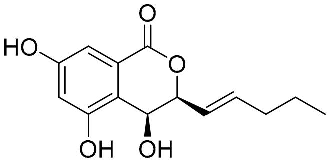

356. Diportharine A

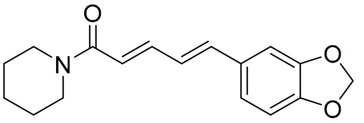

358. Piperine

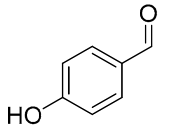

359. *p*-Hydroxy benzaldehyde

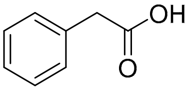

360. Phenyl acetic acid

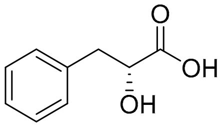

361. Phenyllactic acid

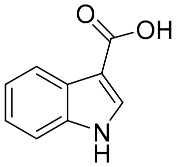

362. Indole-3-carboxylic acid

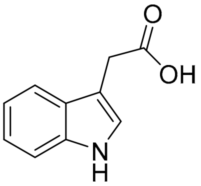

363. Indole-3-acetic acid

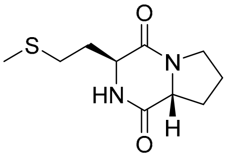

364. Cyclo-(proline-methionine)

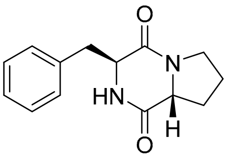

365. Cyclo-(phenylalanine-proline)

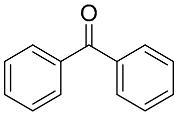

366 Benzophenone

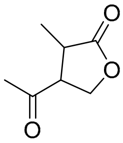

367. 3-Acetyl-2-methyl dihydrofuran-2(3H)-one

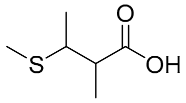

368. 2-Methyl-3-methylthio-butanoic acid

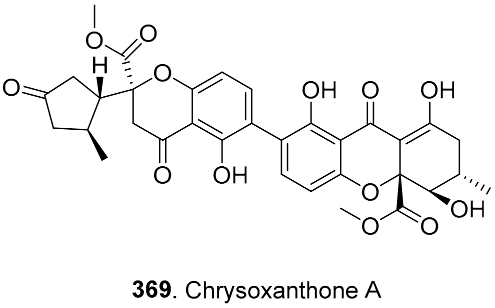

369. Chrysoxanthone A

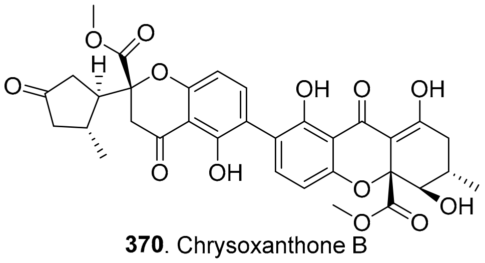

370. Chrysoxanthone B

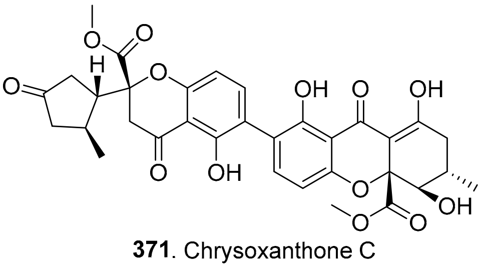

371. Chrysoxanthone C

Figure S6. Structures of the compounds **352–371** isolated from fungi treated with valproic acid (VAP) or sodium valproate (SVP).

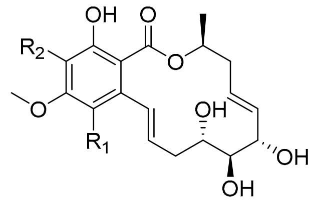

372. 5-Brommozeaenol, R_1=Br, R_2=H
373. 3,5-Dibromozeaenol, R_1=Br, R_2=Br
375. Zeaenol, R_1=H, R_2=H

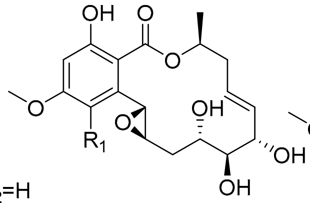

374. Aigialomycin B

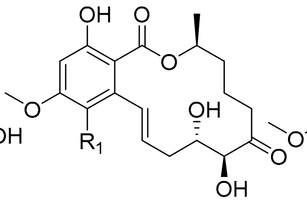

376. LL-Z1640-1

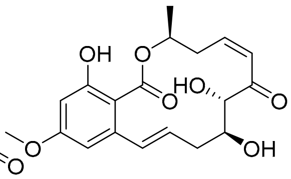

377. LL-Z1640-2

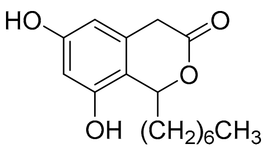

378. Cytosporone C

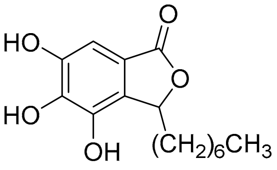

379. Cytosporone E

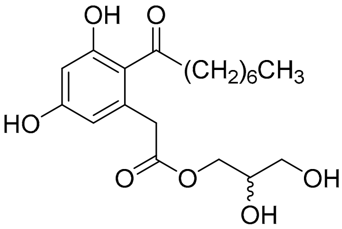

380. Cytosporone R

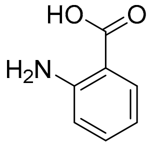

381. Anthranilic acid

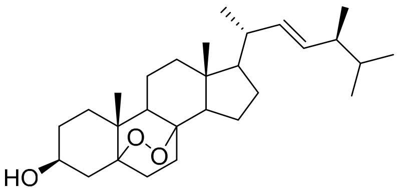

382. Ergosterol peroxide

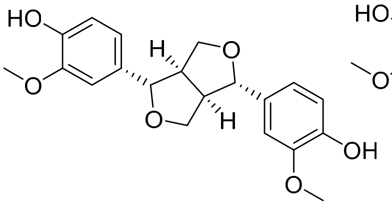

383. Pinoresinol

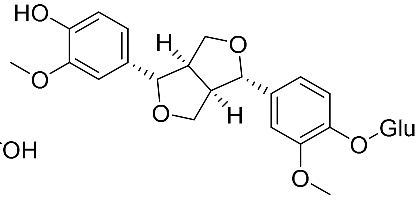

384. Pinoresinol monoglucoside

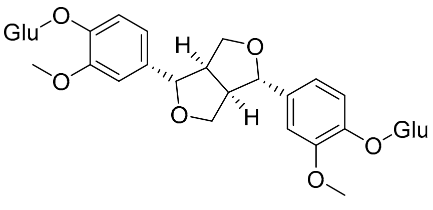

385. Pinoresinol diglucoside

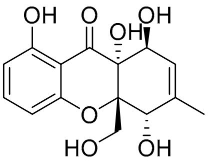

386. Phaseolorin J

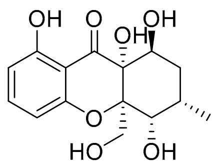

387. Phaseolorin D

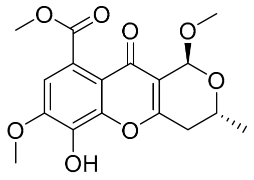

388. Chaetochromone B

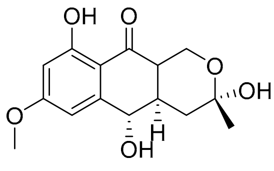

389. Pleosporalin D

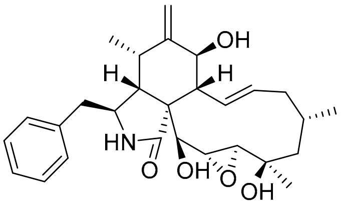

390. Phomoparagin D

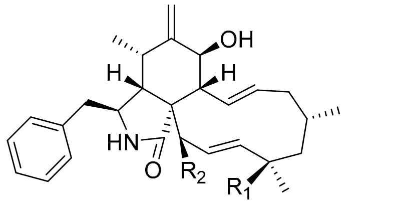

391. Cytochalasin J, R_1=OH, R_2=OH
392. Cytochalasin J1, R_1=OCH_3, R_2=OH
393. Cytochalasin J2, R_1=OH, R_2=OAc

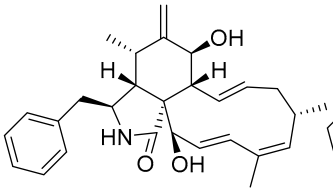

394. Cytochalasin J3

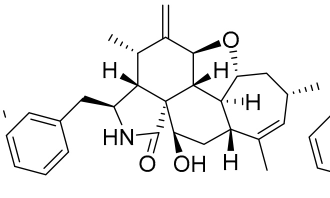

395. Cytochalasin H

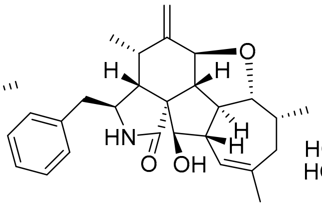

396. Phomopchalasin D

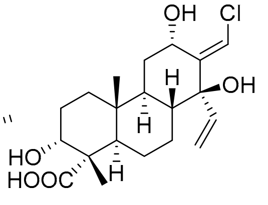

397. Harzianolic acid

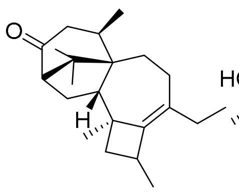

398. Harzianone E

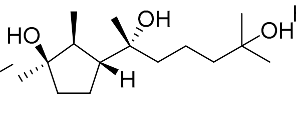

399. 3,7,11-Trihydroxy-cycloneran

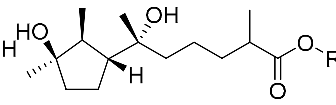

400. Methyl 3,7-dihydroxy-15-cycloneranate, R=CH_3
401. Catenioblin C, R=H

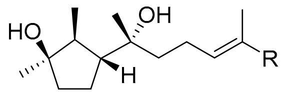

402. Ascotrichic acid, R=COOH
403. Cyclonerotriol R=CH_2OH
404. (10*E*)-12-Acetoxy-10-cyclone-ren-3,7-diol, R=CH_2OCOCH_3
405. Cyclonerodiol R=CH_3

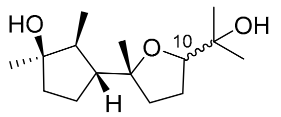

406. Cyclonerodiol oxide, 10*S*
407. Epicyclonerodiol oxide, 10*R*

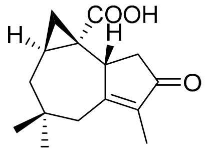

408. Ophioceric acid

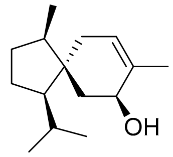

409. *ent*-Trichoacorenol

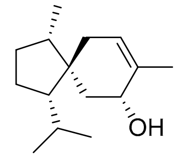

410. Trichoacorenol

Figure S7. Structures of the compounds **372–410** isolated from fungi treated with sodium butyrate (NaBut).

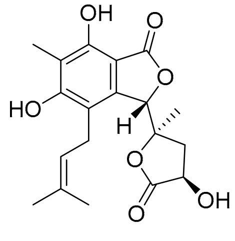

411. Chaetophenol G

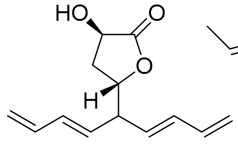

412. Cancrolide A

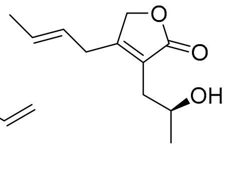

413. Cancrolide B

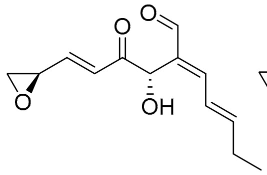

414. Mollipilin A

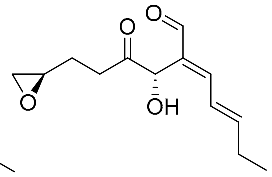

415. Mollipilin B

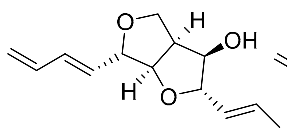

416. Mollipilin C

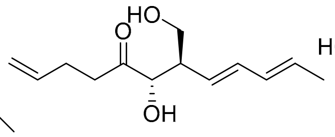

417. Mollipilin D

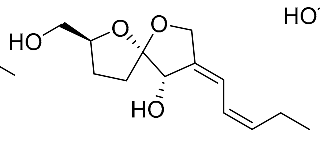

418. Mollipilin E

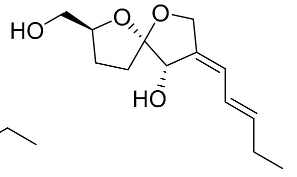

419. Mollipilin F

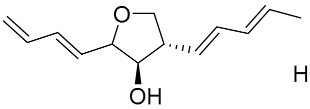

420. Aureonitol

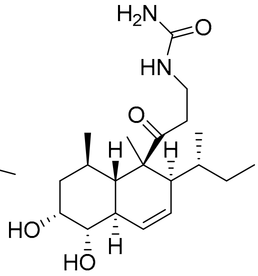

421. Eupenicinicol C

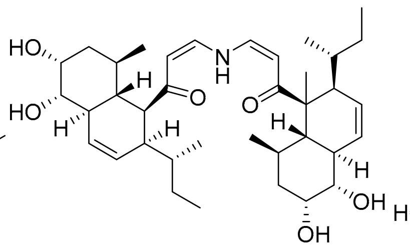

422. Eupenicinicol D

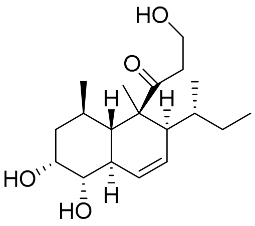

423. Eujavanicol A

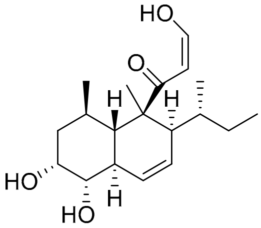

424. Eupenicinicol A

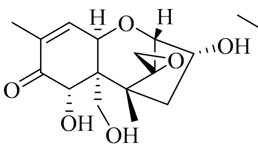

425. Deoxynivalenol

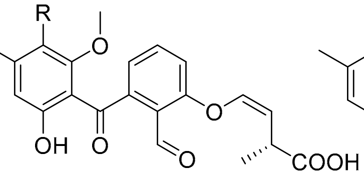

426. Cephalanone A, R=Cl
428. Cephalanone C, R=H

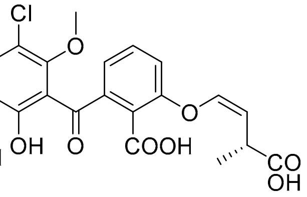

427. Cephalanone B

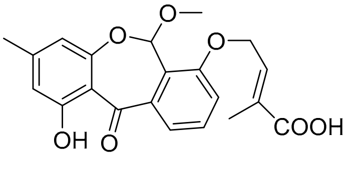

429. Cephalanone D

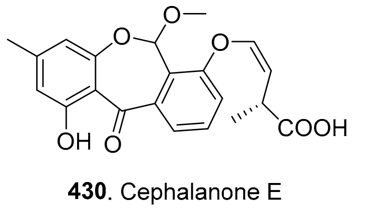

430. Cephalanone E

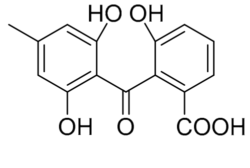

431. Cephalanone F

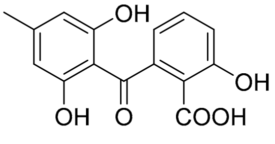

432. 2-(2,6-Dihydroxy-4-methylbenzoyl)-6-hydroxybenzoic acid

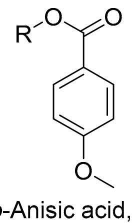

433. *p*-Anisic acid, R=H
434. *p*-Anisic acid methyl ester, R=CH_3

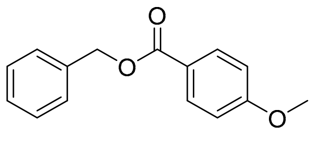

435. Benzyl anisate

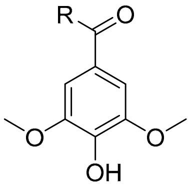

436. Syringic acid, R=OH
438. Acetysyringone, R=CH_3

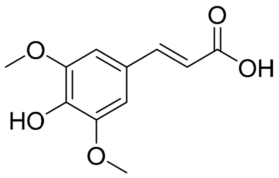

437. Sinapic acid

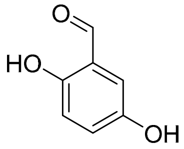

439. Gentisaldehyde

Figure S8. Structures of the compounds **411–439** isolated from fungi treated with nicotinamide.

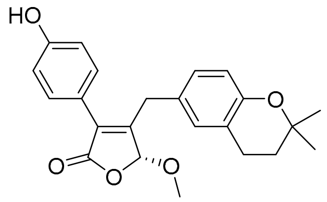

435. (*R*)-4-((2,2-Dimethylchroman-6-yl) methyl)-3-(4-hydroxyphenyl)-5-methoxyfuran-2(5*H*)-one

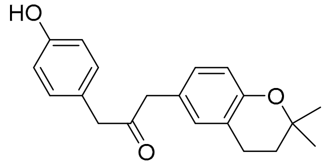

436. 1-(2,2-Dimethylchroman-6-yl)-3-(4-hydroxyphenyl)propan-2-one

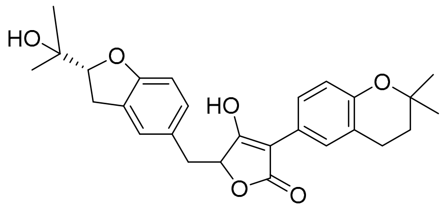

437. (*R,E*)-3-(2,2-Dimethylchroman-6-yl)-4-hydroxy-5-((2-(2-hydroxypropan-2-yl)-2,3-dihydrobenzofuran-5-yl)methylene) furan-2(5*H*)-one

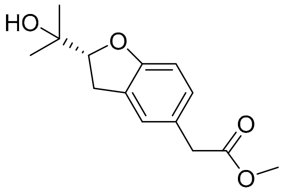

438. Methyl (*R*)-2-(2-(2-hydroxypropan-2-yl)-2,3-dihydrobenzofuran-5-yl) acetate

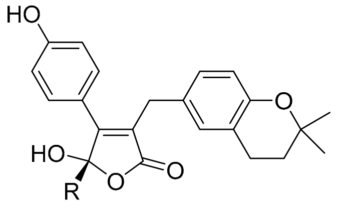

439. Flavipesolide A, R=$COOCH_3$
440. Flavipesolide B, R=$COOCH_2CH_3$
441. Flavipesolide C, R=COOH

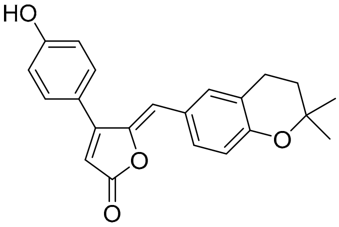

442. Rubrolide S

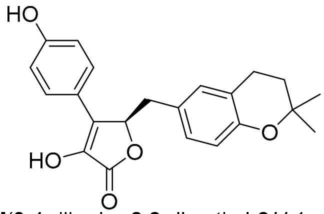

443. 5-[(3,4-dihydro-2,2-dimethyl-2*H*-1-benzopyran-6-yl)- me-thyl]-3-hydroxy-4(4-hydroxyphenyl)-2(5*H*)-furanone

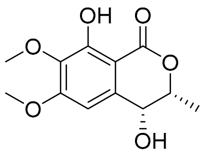

444. (3*R*,4*R*)-3,4-Dihydro-4,8-dihydroxy-6,7-dimethoxy-3-methylisocoumarin

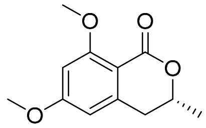

445. (3*R*)-3,4-dihydro-6,8-dimethoxy-3-methylisocoumarin

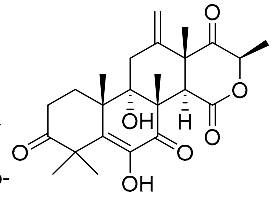

446, Terretonin C

Figure S9. Structures of the compounds **440–451** isolated from fungi treated with trichostatin A (TSA).

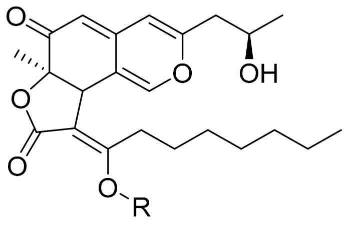

452. Monasfluol B, R=H
453. Acetyl monasfluol B, R=Ac

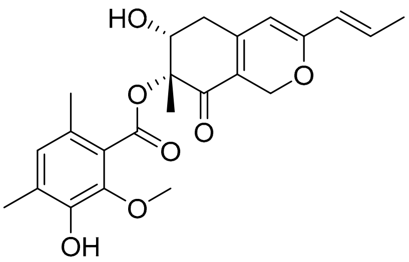

454. Azaphilone C

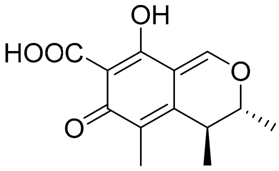

455. Citrinin

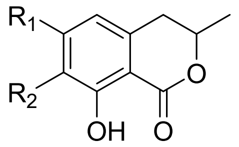

456. 6-Demethylkigelin R_1=OH, R_2=OCH_3
457. (3R)-6,7-Dimethoxymellein, R_1=R_2=OCH_3

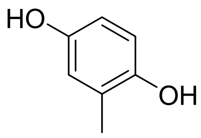

458. Pyrolin

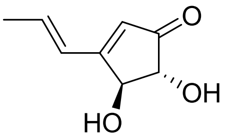

459. Terrain

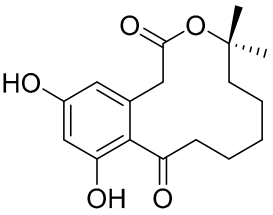

460. Curvularin

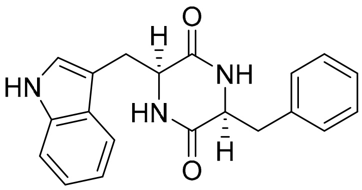

461. Cyclo-(L-Trp-L-Phe)

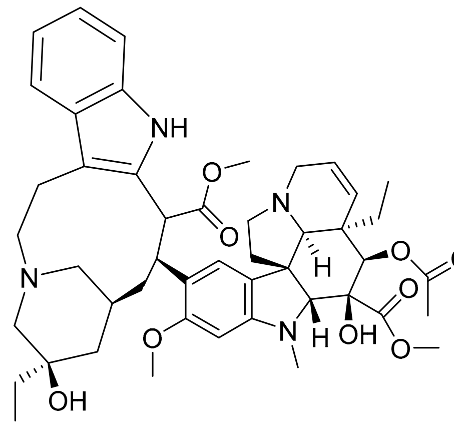

462. Vinblastine

Figure S10. Structures of the compounds **452–462** isolated from fungi treated with other histone deacetylase modifiers.

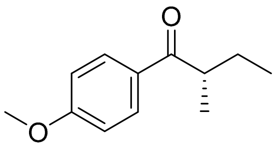

463. 4'-Methoxy-(2*S*)-methylbutyrophenone

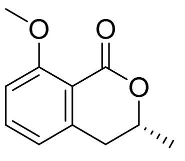

464. (3*R*)-Mellein methyl ether

Figure S11. Structures of the compounds **463** and **464** isolated from fungi treated with two histone deacetylase modifiers.

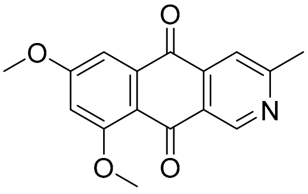

465. Scorpinone

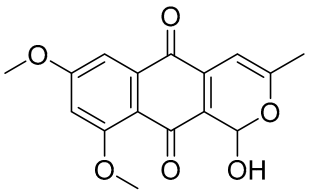

466. 1-Hydroxydehydroherbarin

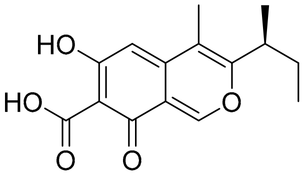

467. Ascochitine

Figure S12. Structures of the compounds **465–467** isolated from fungi treated with histone acetyltransferase modifier anacardic acid.

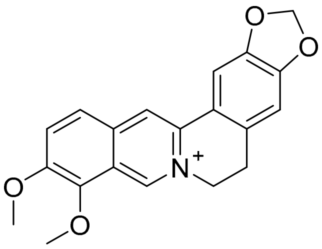

468. Berberine

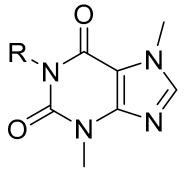

469. Caffeine, R=CH3
470. Theobromine, R=H

Figure S13. Structures of the compounds **468–470** isolated from fungi treated with histone methyltransferase modifier BRD4770.

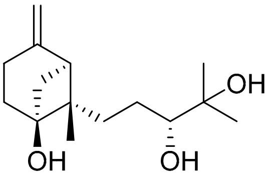

471. Xylariterpenoid H

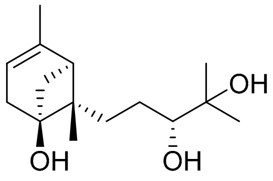

472. Xylariterpenoid I

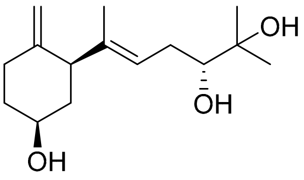

473. Xylariterpenoid J

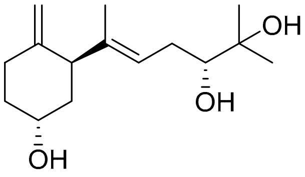

474. Xylariterpenoid K

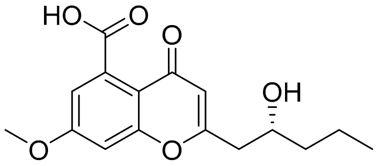

475. (*R*)-2-(2-Hydroxypentyl)-5-carboxy-7-methoxychromone

Figure S14. Structures of the compounds **471–475** isolated from fungi treated with proteasome modifier bortezomib.

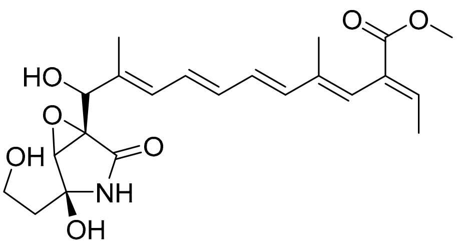

476. DihydroNG391

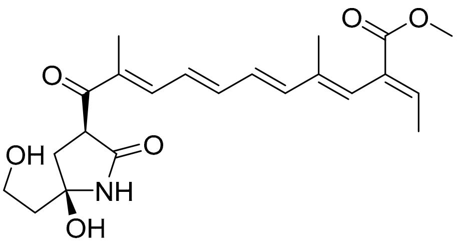

477. Dihydrolucilactaene

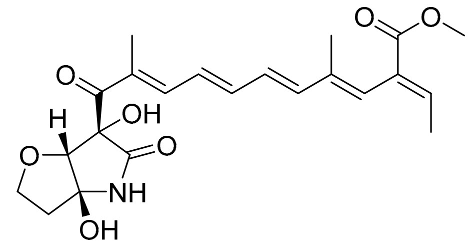

478. 13α-Hydroxylucilactaene

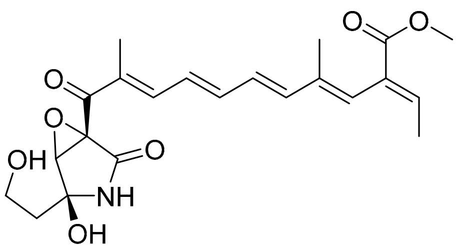

479. NG391

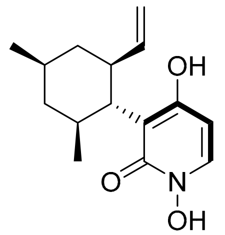

480. *P*-Pyridoxatin

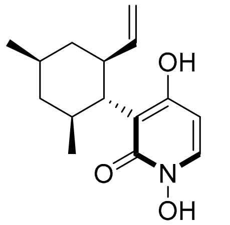

481. *M*-Pyridoxatin

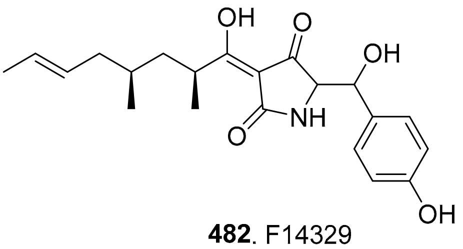

482. F14329

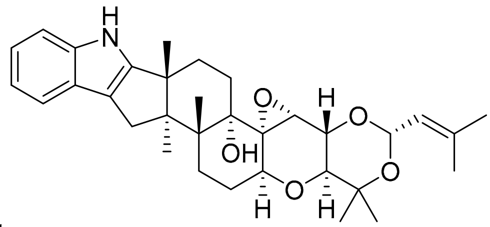

483. Terpendole C

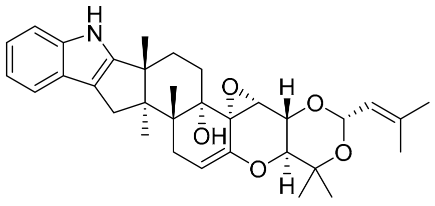

484. Terpendole K

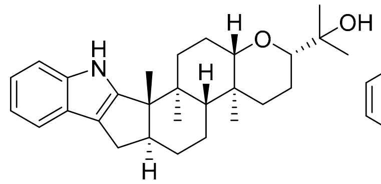

485. Paspaline

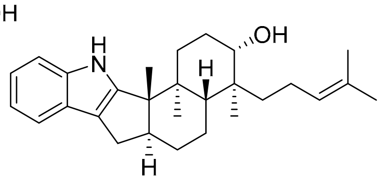

486. Emindole SB

Figure S15. Structures of the compounds **476–486** isolated from fungi treated with NPD938.

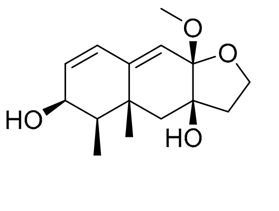

487. Dihydrobipolaroxin B

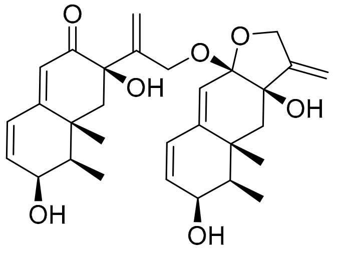

488. Dihydrobipolaroxin C

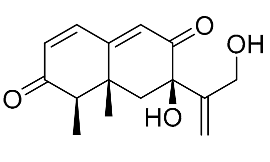

489. Dihydrobipolaroxin D

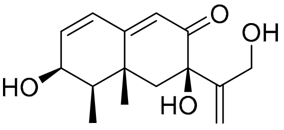

490. Dihydrobipolaroxin

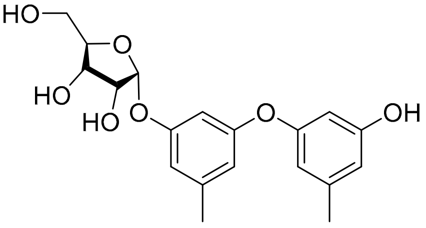

491. Diorcinol-3-*O*-α-D-ribofuranoside

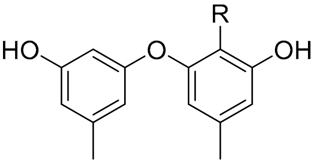

492. Diorcinal, R=H
494. Cordyol, R=OH

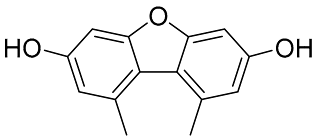

493. 3,3'-Dihydroxy-5,5'-dimethyldibenzofuran

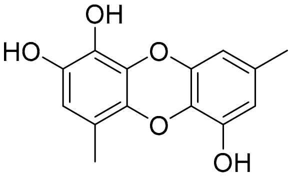

495. Gibellulin B

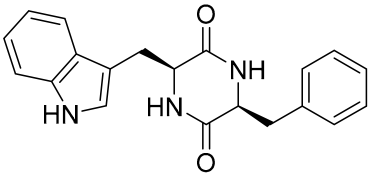

496. Cyclo-(L-Trp-L-Phe)

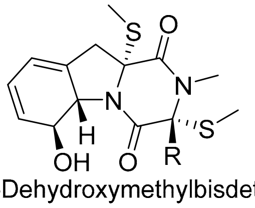

497. 3-Dehydroxymethylbisdethio-3,10a-bis(methylthio) gliotoxin, R=H
498. Bisdethiobis (methylthio) gliotoxin, R=CH_2OH

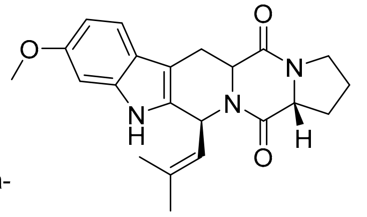

499. Fumitremorgin C

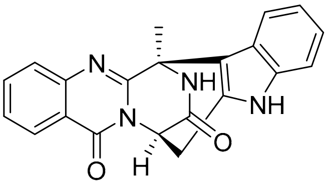

500. Fumiquinazoline J

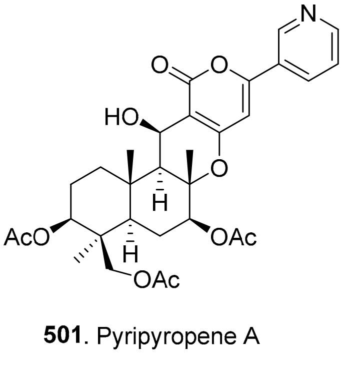

501. Pyripyropene A

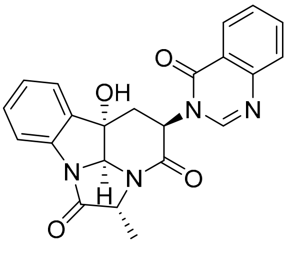

502. Chaetominine

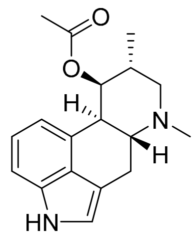

503. Fumigaclavine A

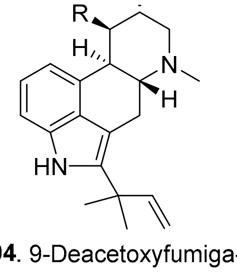

504. 9-Deacetoxyfumigaclavine C, R=H
505. Fumigaclavine C, R=$OCOCH_3$

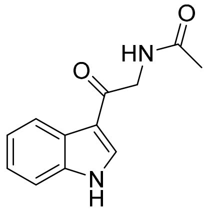

506. 3-Hydroxyacetyl indole

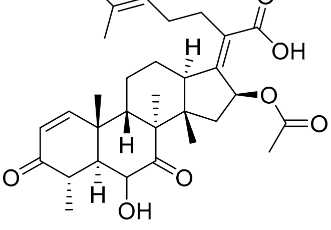

507. Helvolinic acid

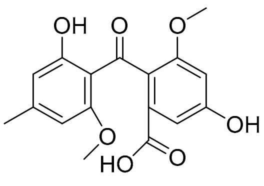

508. Rhizoctonic acid,

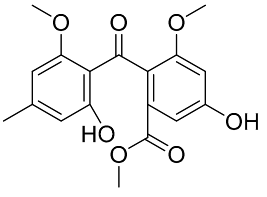

509. Monomethylsulochrin

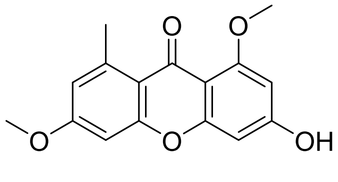

510. 3-Hydroxy-1,6-dimethoxy-8-methyl 9*H*-xanthen-9-one

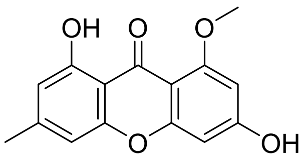

511. Questin

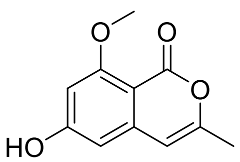

512. 6-Hydroxy-8-methoxy-3-methylisocoumarin

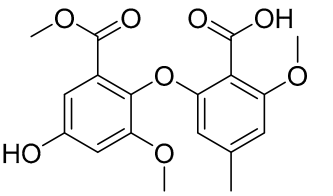

513. Circinoporic acid

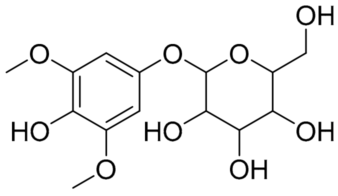

514. Koaburaside

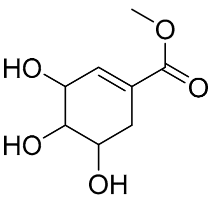

515. Methyl shikimate

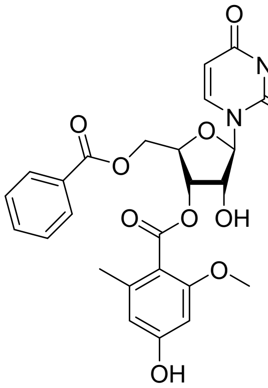

516. Kipukasin K

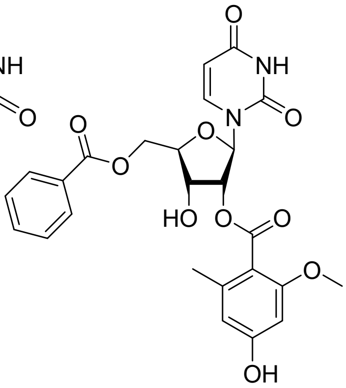

517. Kipukasin L

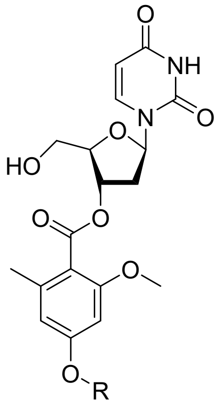

519. Kipukasin I, R=H
521. Kipukasin D, R=CH_3

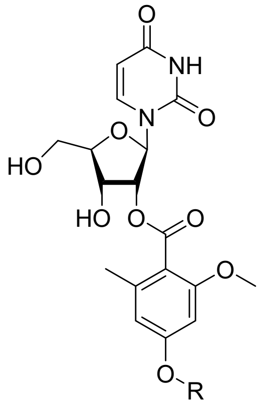

520. Kipukasin H, R=H
522. Kipukasin E, R=CH_3

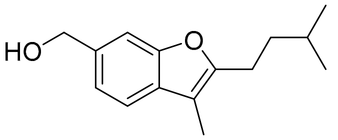

518. Aspergillusene E

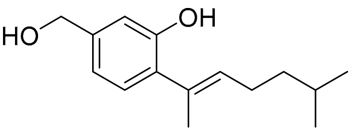

523. (*E*)-5-(Hydroxymethyl)-2-(6'-methylhept-2'-en-2'-yl) phenol

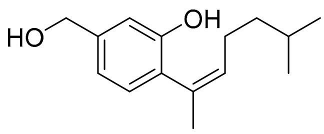

524. (*Z*)-5-(Hydroxymethyl)-2-(6'-methylhept-2'-en-2'-yl) phenol

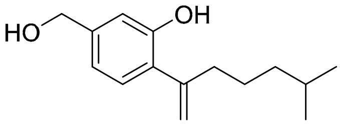

525. 7-Deoxy-7,14-didehydrosydonol

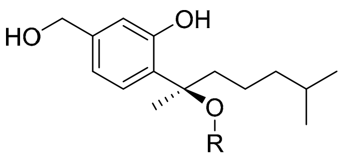

526. (7*R*)-Sydonol, R=H
527. (7*R*)-Methoxysydonol, R=CH_3

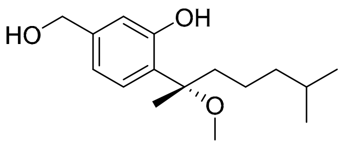

528. (7*S*)-Methoxysydonol

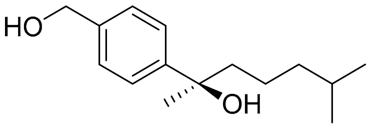

529. Aspergiterpenoid A

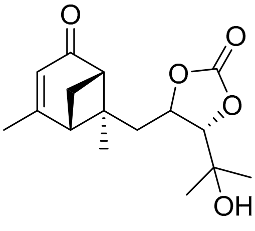

530. Eutypeterpene A

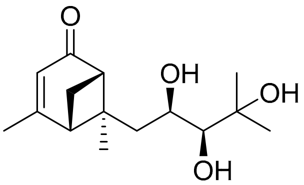

531. Eutypeterpene B

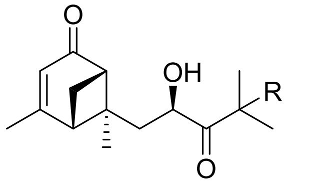

532. Eutypeterpene C, R=OH
533. Eutypeterpene D, R=H

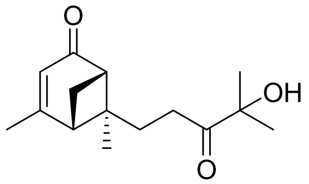

534. Eutypeterpene E

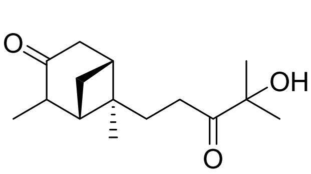

535. Eutypeterpene E

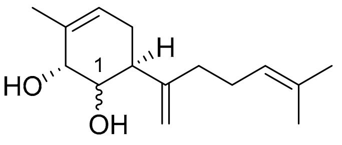

536. Eutypeterpene G, 1*S*
537. Eutypeterpene H, 1*R*

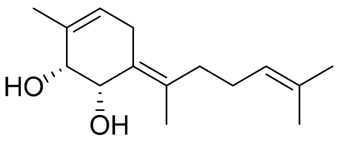

538. Eutypeterpene I

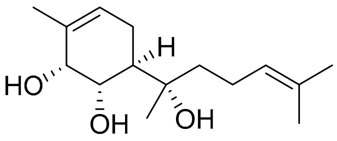

539. Eutypeterpene J

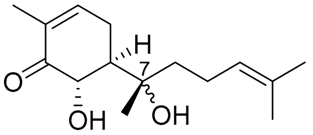

540. Eutypeterpene K, 7*S*
541. Eutypeterpene L, 7*R*

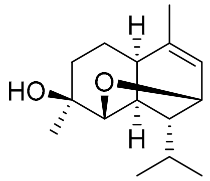

542. Eutypeterpene M

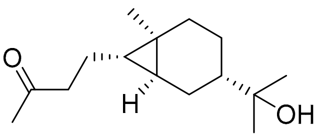

543. Eutypeterpene N

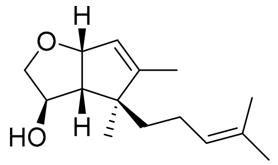

544. Eutypeterpene O

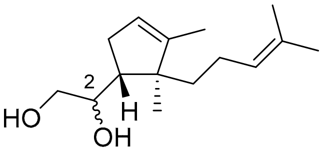

545. Eutypeterpene P, 2*S*
546. Eutypeterpene Q, 2*R*

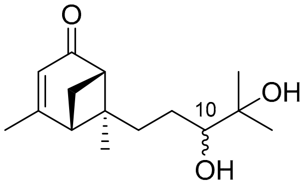

547. Xylariterpene A, 10*S*
548. Xylariterpene B, 10*R*

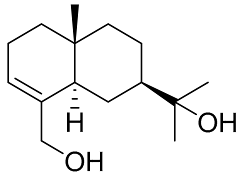

549. Eudesma-3-en-11,15-diol

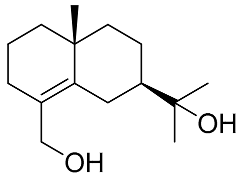

550. Eudesma-4-en-11,15-diol

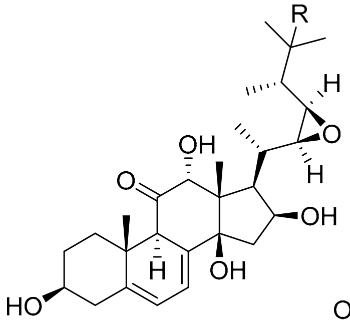

551. Formosterol A, R=OH
558. Formosterol C, R=H

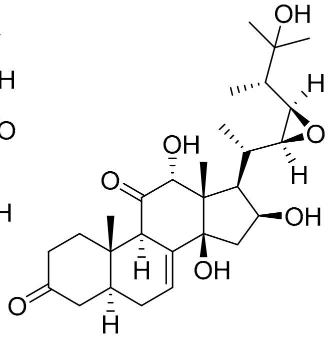

552. Formosterol B

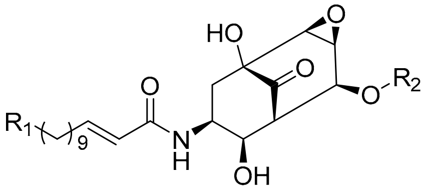

553. 12'-*O*-Acetylisariotin A, R_1=OAc, R_2=H
561.TK-57-164A, R_1=H, R_2=CH_3

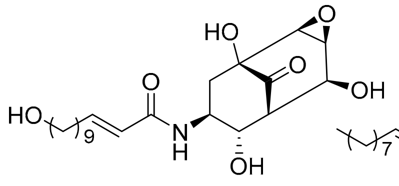

554. 1-*epi*-Isariotin A

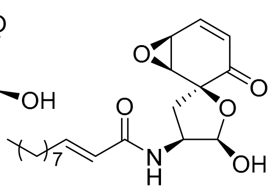

555. Isariotin K, R=H
560. Isariotin E, R=CH_2CH_3

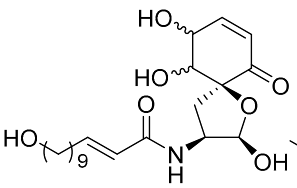

556. Isariotin L

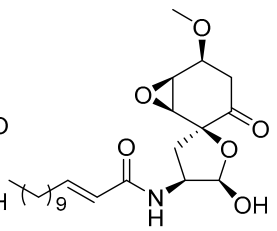

557. Isariotin M

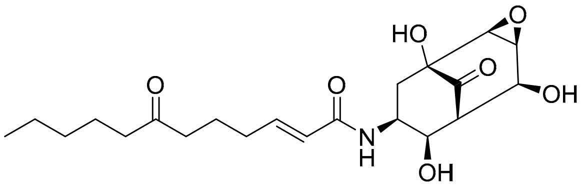

559. Isariotin C

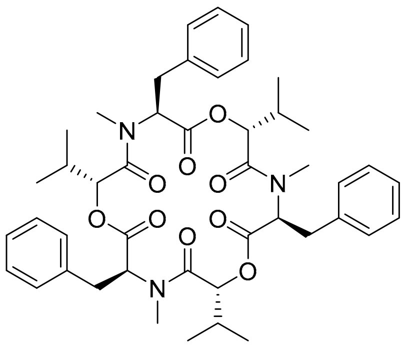

562. Beauvericin

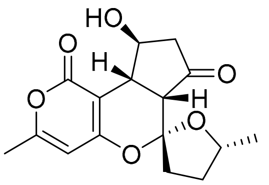

563. Tenuipyrone

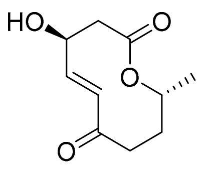

564. Cephalosporolinde B

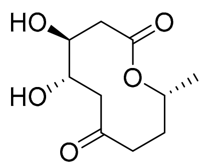

565. Cephalosporolinde F

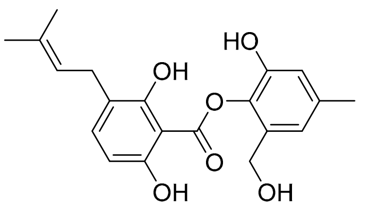

566. 20-Hydroxy-6'-hydroxymethyl-4'-methylphenyl-2,6-dihydroxy-3-(2-isopentenyl)benzoate

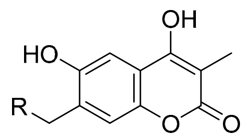

567. 4,6-Dihydroxy-7-hydroxymethyl-3-methylcoumarin, R=OH
568. 4,6-Dihydroxy-3,7-dimethylcoumarin, R=H

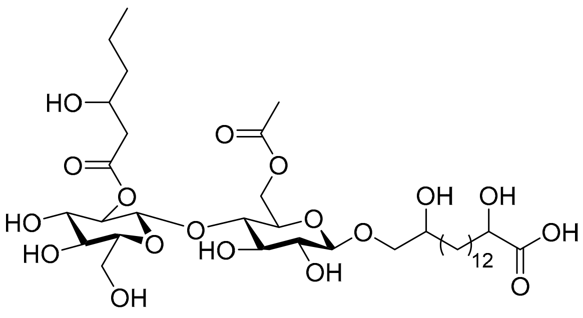

569. Ustilagic acid B

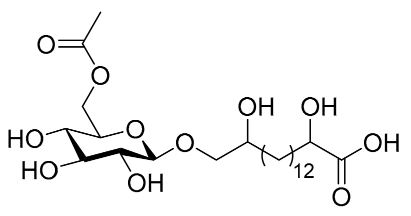

570. Ustilagic acid C

Figure S16. Structures of the compounds **487–570** isolated from fungi treated with two types of chemical epigenetic modifiers.

Table S1. The examples of 5-Aza affecting production of fungal secondary metabolites.

Fungal Species	Concentration of 5-Aza	Compounds Induced/Enhanced/Decreased	Ref.
Alternaria sp.	250 µM	Induced production of alternariol (**26**), alternariol-5-*O*-methyl ether (**27**), 3′-hydroxyalternariol-5-*O*-methyl ether (**28**), altenusin (**29**), tenuazonic acid (**30**), and altertoxin II (**31**).	[51]
Aspergillus sp. XS-20090066	2 µM	Induced production of (7*R*)-hydroxysydonic acid (**32**), (7*S*)-sydonic acid (**33**), (*S*)-5-(hydroxymethyl)-2-(2′,6′,6′-trimethyltetrahydro-2*H*-pyran-2-yl)phenol (**34**), (7*S*,11*S*)-12-hydroxysydonic acid (**35**), (7*S*)-11-dehydrosydonic acid (**36**), and (*S*)-sydowic acid (**37**).	[52]
Aspergillus clavatus	2 µM	Enhanced production of cytochalasin E (**38**), patulin (**39**) and pseurotin A (**40**).	[25]
A. flavus	1 mM	Inhibited production of aflatoxin B1 (**41**).	[53-55]
A. parasiticus	1 mM	Inhibited production of aflatoxin B1 (**41**).	[56]
A. niger	1 µM	Enhanced production of (*Z*)-9-octadecenoic acid (**42**) and 12-methyl-tetradecanoic acid methylester (**43**).	[15]
A. sydowii	100 µM	Induced production of three new bisabolane-type sesquiterpenoids namely (7*S*)-sydonic acid (**33**), (7*S*)-7-*O*-methylsydonol (**44**), (7*S*,11*S*)-12-hydroxysydonic acid (**45**) and 7-deoxy-7,14-didehydrosydonol (**46**), along with eight known compounds including (*S*)-sydonol (**47**), anhydrowaraterpol B (**48**), (*E*)-5-(hydroxymethyl)-2-(6′-methylhept-2′-en-2′-yl)phenol (**49**), AGI-B4 (**50**), sydowinin A (**51**), sydowinin B (**52**), and diorcinol (**53**).	[57]
A. terreus GZU-31-1	50 µM	Induced production of five butanolide derivatives namely asperbutyrolactones A (**54**) and B (**55**), aspulvinone E (**56**), butyrolactone I (**57**), and butyrolactone VI (**58**), and four diphenyl ether derivatives namely, asterric acid (**59**), penicillither (**60**), methyl asterrate (**61**) and 4′,6′-dichloroasterric acid (**62**).	[58]
Botryosphaeria rhodina	10 mg/L	Enhanced production of camptothecine (**63**).	[59]
Chaetomium sp.	6 mM	Enhanced production of isosulochrin (**64**).	[60]
Chaetomium globosporum	120 mg/L	Induced production of two cyclopentenones globosporins A (**65**) and B (**66**) and two monoterpenoid indole alkaloids globosporines C (**67**) and D (**68**), as well as three known compounds pseurotin A (**40**), mappianine E (**69**), and 19(20)*Z*-5-carboxymethylvallesiachotamine (**70**).	[61]
Cladosporium cladosporioides	0.1 µM–10 mM	Induced production of oxylipins: (9*Z*,12*Z*)-11-hydroxyoctadeca-9,12-dienoic acid (**71**), its methyl ester (**72**), and glycerol conjugate (**73**)	[26]
Cochliobolus lunatus TA26–46	10 µM	Induced production of new diethylene glycol phthalate esters, cochphthesters A–G (**74**–**80**), along with four known analogues: 1,2-benzenedicarboxylic acid,1,2-bis [2-(2-hydroxyethoxy) ethyl] ester (**81**), 1,2-benzenedicarboxylic acid, 1,1 -(oxydi-2,1-ethanediyl) 2,2 -bis [2-(2-hydroxyethoxy) ethyl] ester (**82**), 1,2-benzenedicarboxylic acid, 1,2-bis[2-[[2-[[2-(2-hydroxyethoxy) ethoxy] carbonyl] oxy] ethoxy] ethyl] ester (**83**), and 1,2-benzenedicarboxylic acid, oxydi-2,1-ethanediyl dimethyl ester (**84**).	[62]

Cochliobolus lunatus TA26-46	10 μM	Induced production of two α-pyrones namely cochliobopyrones A (**85**) and B (**86**), three isocoumarins namely 3-methyl-6,8-dihydroxyisocoumarin (**87**), 6-hydroxy-8-methoxy-3-methylisocoumarin (**88**), (*S*)-orthosporin (**89**), and one chromone altechromone A (**90**).	[63]
Cophinforma mamane	1 μM	Induced production of three thiodiketopiperazines: botryosulfuranols A (**91**), B (**92**) and C (**93**).	[64]
Cordyceps indigotica	100 μM	Induced production of aromatic polyketide glycosides: indigotides A (**94**) and B (**95**).	[65]
Diaporthe perseae	1 μM	Increased production of colchicine (**96**).	[66]
Diatrype sp.	0.1–10 μM	Induced production of new glycosylated polyketides namely lunalides A (**97**) and B (**98**).	[26]
Dimorphosporicola tragani CF-090383	100 μM	Induced production of dendrodolides E (**99**), G (**100**), and I (**101**).	[67]
Lophiotrema sp. F6932	50 μM	Induced production of spirobisnaphthalenes: palmarumycins CP30 (**102**) and C8 (**103**).	[68]
Muscodor yucatanensis Ni30	50 μM	Enhanced production of ergosterol (**104**) and xylaguaianol C (**105**).	[69]
Neosartorya udagawae HDN13-313	73 mg/L	Induced production of 3-methoxy-6-methyl-5-(methylsulfonyl)benzene-1,2,4-triol (**106**) and neosartoryone A (**107**).	[70]
Penicillium citreonigrum	50 μM	Enhanced production of sclerotiorin (**108**), ochrephilone (**109**), dechloroisochromophilone III (**110**), dechloroisochromophilone IV (**111**), 6-((3*E*,5*E*)-5,7-dimethyl-2-methylenenona-3,5-dienyl)-2,4-dihydroxy-3-methylbenzaldehyde (**112**), sclerotioramine (**113**), pencolide (**114**), atlantinones A (**115**) and B (**116**).	[35]
Penicillium minioluteum	500 μM	Induced production of new aspertetronin dimers: miniolins A–C (**117–119**), along with their precursor aspertetronin A (**120**).	[71]
Penicillium variabile HXQ-H-1A	0.2 mM	Induced production of a highly modified fatty acid amide, varitatin A (**121**).	[72]
Pestalotiopsis crassiuscula	500 mM	Induced production of 4,6-dihydroxy-3,7-dimethylcoumarin (**122**), pestalotiopyrone G (**123**) and 2′-hydroxy-6′-hydroxy-methyl-4′-methylphenyl 2,6-dihydroxy-3-(2-isopentenyl)benzoate (**124**).	[73]
Pestalotiopsis microspora	500 μM	Induced production of 4′-formamidophenyl-5-methoxybenzoate (**125**), pestalotiopyrone G (**123**), 4-hydroxybenzoic acid (**126**), LL-P880*α* (**127**), 2′-hydroxy 6′-hydroxymethyl-4′-methylphenyl-2,6-dihydroxy-3-(2-isopentenyl)benzoate (**128**), pestalotiollide B (**129**), endocrocin (**130**) and 1′-hydroxy-4-methoxy-6-pentyl-2*H*-pyran-2-one (**131**).	[74]
Phomopsis sp. MD 86	10 mg/L	Enhanced production of camptothecine (**63**).	[59]
Xylaria psidii	10 μM	Enhanced production of resveratrol (**132**).	[75]

Table S2. The examples of SAHA affecting production of fungal secondary metabolites.

Fungal Species	Concentration of SAHA	Compounds Induced/Enhanced/Decreased	Ref.
Aspergillus sp. AST0006	250 µM	Induced production of two new 3-(4-oxopyrano)-chromen-2-ones namely aspyranochromenones A (**150**) and B (**151**), along with nine known metabolites namely 6,7-dihydroxymellein (**152**), terrein (**153**), (3*R*)-6-hydroxymellein (**154**), (*R*)-orthosporin (**155**), 6,7-dimethoxymellein (**156**), 6-methoxymellein (**157**), (2*E*,6*E*,10*E*)-12-hydroxyfarnesol (**158**), 5,6-dihydroxymellein (**159**), and ethyl 3-methylorsellinate (**160**).	[81]
A. calidoustus	100 µM	Increased production of phenylahistin (**161**).	[82]
A. nidulans	100 µM	Induced production of fellutamides fellutamide B (**163**), antibiotic 1656G (**164**) and antibiotic 3127 (**165**).	[83]
A. nidulans	100 µM	Induced production of aspercryptins A1 (**166**) and A2 (**167**).	[84]
A. niger ATCC1015	10 µM	Induced production of nygerone A (**168**).	[41]
A. terreus PF26	500 µM	Enhanced production of terrein (**153**) and (3*R*)-6-hydroxymellein (**154**).	[85]
A. terreus RA2905	100 µM	Induced production of (+)- and (-)-asperfuranones (**169** and **170**) together with two benzyl pyrones, asperpyranones A (**171**) and B (**172**).	[86]
A. versicolor MCCC 3A00080	20 mg/L	Induced production of versiperol A (**173**), diorcinol (**53**) and 2,4-dimethoxyphenol (**174**).	[87]
A. versicolor OUCMDZ-2738	10 µM	Induced production of diorcinol (**53**), 3-[6-(2-methylpropyl)-2-oxo-1*H* pyrazin-3-yl] propanamide (**175**), brevianamide X (**176**), brevianamide R (**177**), brevianamide Q (**178**), diorcinol C (**179**), diorcinol E (**180**), and methyl diorcinol-4-carboxylate (**181**).	[88]
A. wentii na-3	20 µM	Induced production of aspewentins A (**182**) and B (**183**), along with an oxygenated derivative, aspewentin C (**184**).	[89]
A. westerdijkiae	100 µM	Increased production of penicillic acid (**162**).	[82]
Asteromyces cruciatus	100 µM	Induced production of primarolides A (**185**) and B (**186**).	[91]
Beauveria felina	500 µM	Induced production of eight cyclodepsipeptides: desmethylisaridin E (**187**), isaridin E (**188**), desmethylisaridin C2 (**189**), isaridin C2 (**190**), isaridin F (**191**), destruxin A (**192**), reseotoxin B (**193**), and roseocardin (**194**).	[92]
Bjerkandera adusta	300 µM	Induced production of six tremulane sesqiterpenoids: 11,12-dihydroxy-1-tremulen-5-one (**195**), (3*S*,6*R*,7*R*)-tremul-1-ene-6,11,12-triol (**196**), ceriponol A (**197**), conocenol B (**198**), and conocenolides A (**199**) and B (**200**)	[93]
Botrytis cinerea B05.10	50–200 µM	Induced production of botrycinereic acid (**201**).	[10]
Chaetomium sp.	6 mM	Enhanced production of isosulochrin (**64**).	[60]
Chalara sp. 6661	1 mM	Induced production of chalanilines A (**202**) and B (**203**), and adenosine-coupled xanthones A (**204**) and B (**205**).	[94]
Cladosporium cladosporioides	10 mM	Induced production of cladochromes F (**206**) and G (**207**), cladochromes A (**208**), B (**209**), D (**210**), and E (**211**) and calphostin B (**212**).	[26]
Cladosporium sphaerospermum L3P3	300 µM	Induced production of cladosins H–K (**213**–**216**) and cladodionen (**217**).	[95]

Claviceps purpurea Cp-1	500 µM	Enhanced production of ergometrine (**218**).	[96]
Cyathus stercoreus	200 µM	Induced production of nine novel sesquiterpenoids: cystercorolide (**219**), cystercorodiol A (**220**), 4-*O*-acetylcybrodol (**221**), 14-dehydroxycybrodol (**222**), cystercorodiol B (**223**), 4-*O*-acetylcystercorodiol B (**224**), 1-*O*-methoxycystercorodiol B (**225**), cystercorodiol C (**226**), and cystercorotone (**227**) along with four known sesquiterpenes, epicoterpene D (**228**), russujaponol F (**229**), riparol B (**230**), and cybrodol (**231**).	[97]
Daldinia sp.	800 µM	Induced production of daldinone E (**232**).	[98]
Drechslera sp.	500 µM	Induced production of hexosylphytosphyngosine (**233**).	[40]
Lachnum palmae	500 µM	Induced production of eighteen dihydroisocoumarins: palmaerones A–G (**234–240**), mellein (**18**), (3*R*)-6-hydroxymellein (**154**), (*R*)-6-methoxymellein (**157**) (*R*)-5-cholro-6-hydroxymellein (**241**), (3*R*,4*R*)-5-cholro-4,6-dihydroxymellein (**242**), palmaerin A (**243**), palmaerin B (**244**), palmaerin D (**245**), *trans*-4-hydroxymellein (**246**), *cis*-4-hydroxymellein (**247**), and (*R*)-5-hydroxymellein (**248**).	[99]
Microascus sp.	100 µM	Induced production of a cyclic pentadepsipeptide namely EGM-556 (**249**).	[42]
Muscodor yucatanensis Ni30	50 µM	Induced production of ergosterol (**104**) and xylaguaianol C (**105**).	[69]
Penicillium sp. HS-11	80 mg/L	Induced production of 4-epipenicillone B (**250**) and (*R*)-chrysogine (**251**).	[100]
Penicillium sp. KMU18029	100 µM	Induced production of meroditerpenoids: pyrandecarurins A (**252**) and B (**253**), pileotins A (**254**) and B (**255**), along with their potential precursor decaturenoid (**256**), as well as the known meroterpenoids 15-hydroxydecaturin A (**257**), oxalicines A (**258**) and B (**259**), and penisarin B (**260**).	[101]
Penicillium brasilianum	200 µM	Induced production of brasiliamide A (**261**), verruculogen TR2 (**262**), and penicillic acid (**162**).	[27]
Pencillium mallochii CCH01	1 mM	Induced production of sclerotiorin (**108**), sclerotioramine (**113**), and isochromophilones XIV (**263**) and XV (**264**).	[102]
Penicillium variabile HXQ-H-1	300 µM	Induced production of seven polyketides, including four new ones namely varilactones A (**265**) and B (**266**) and wortmannilactones M (**267**) and N (**268**), as well as three biogenetically related known wortmannilactones E (**269**), F (**270**) and H (**271**).	[103]
Phoma sp. LG0217	500 µM	Induced production of (10′*S*)-verruculide B (**272**), vermistatin (**273**) and dihydrovermistatin (**274**).	[104]
Spiromastix sp.	500 µM	Induced production of nine new guaine-type sesquiterpenes named spiromaterpenes A (**277**), B (**278**), C (**279**), D (**280**), E (**281**), F (**282**), G (**283**), H (**284**), and I (**285**).	[105]
Talaromyces funiculosus HPU-Y01	300 µM	Induced production of funitatin A (**286**).	[106]
Talaromyces wortmannii	100 µM	Induced production of wortmannilactones I–L (**287–290**).	[107]
Xylaria psidii	5 µM	Increased production of resveratrol (**132**).	[75]

Table S3. The examples of SBHA affecting production of fungal secondary metabolites.

Fungal Species	Concentration of SBHA	Compounds Induced/Enhanced/Decreased	Ref.
Alternaria sp.	250 µM	Induced production of alternariol (**26**), alternariol-5-*O*-methyl ether (**27**), 3′-hydroxyalternariol-5-*O*-methyl ether (**28**), altenusin (**29**), tenuazonic acid (**30**), and altertoxin II (**31**).	[51]
Arthrobotrys foliicola	500 µM	Induced production of 4-ethyl-7-hydroxy-8-methyl-2*H*-chromen-2-one (**291**).	[109]
Chaetomium indicum	500 µM	Induced production of chaetophenols A–F (**292–297**).	[110]
C. indicum	500 µM	Induced production of spiroindicumides A (**298**) and B (**299**).	[111]
Cladosporium cladosporioides	0.1–10 mM	Induced production of cladochromes A (**208**), B (**209**), D (**210**), E (**211**), F (**206**), and G (**207**), and calphostin B (**212**).	[26]
Cladosporium colocasiae	500 µM	Induced production of (3β,7α)-cholest-5-en-23-yne-3,7-diol (**300**) and (3β,7α)-cholest-5-en-23-yne-3,7,25-triol (**301**).	[112]
Cordyceps annullata	500 µM	Induced production of four 2,3-dihydrobenzofurans, annullatins A–D (**302–305**), and one aromatic polyketide, annullatin E (**306**).	[113]
Cordyceps indigotica	1 mM	Induced production of six novel aromatic polyketides namely indigotides C–F (**307–310**), 13-hydroxyindigotide A (**311**), and 8-*O*-methylindigotide B (**312**) along with indigotides A (**94**) and B (**95**)	[114]
Eutypella sp. MCCC 3A00281	1 mM	Induced production of 26 eremophilane-type sesquiterpenoids namely eutyperemophilanes A–Z (**313–338**).	[115]
Fusarium oxysporum sp. conglutinans	500 µM	Induced production of two fusaric acid derivatives namely 5-butyl-6-oxo-1,6-dihydropyridine-2-carboxylic acid (**339**) and 5-(but-9-enyl)-6-oxo-1,6-dihydropyridine-2-carboxylic acid (**340**).	[116]
Gibellula formosana	1 mM	Enhanced production of isariotin A (**341**).	[38]
Paraconiothyrium brasiliense	500 µM	Induced production of JBIR-54 (**342**).	[117]
Phomopsis sp. 0391	500 µM	Induced production of new metabolite 13-angeloyloxy-diplosporin (**343**) and six known 3-methoxymethyl-agistatine D (**344**), gynuraone (**345**), mellein (**18**), cytosporone B (**346**), dothiorelone A (**347**), and dothiorelone C (**348**).	[118]
Torrubiella luteorostrata	1 µM	Induced production of three new prenylated tryptophan analogs, luteorides A–C (**349–351**).	[119]

Table S4. The examples of VPA or SVP affecting production of fungal secondary metabolites.

Fungal Species	VPA/SVP and its Concentration	Compounds Induced/Enhanced/Decreased	Ref.
Aspergillus clavatus	VPA, 60 µM	Enhanced production of cytochalasin E (**38**).	[25]
Aspergillus fumigatus GA-L7	VPA, 500 µM	Induced production of fumiquinazoline C (**352**).	[120]
Cordyceps militaris	VPA, 50 µM	Induced production of cordycepin (**353**).	[121]
Diaporthe sp.	VPA, 100 µM	Induced production of three novel cytotoxic secondary metabolites namely xylarolide A (**354**), diportharine A (**355**) and xylarolide B (**356**), along with one known compound xylarolide (**357**).	[122]
Diaporthe sp. PF20	VPA, 100 µM	Enhanced production of piperine (**358**).	[123]
Dimorphosporicola tragani CF-090383	VPA, 100 µM	Induced production of dendrodolides E (**99**), G (**100**) and I (**101**).	[67]
Doratomyces microspora	VPA, 50 µM	Enhanced production of *p*-hydroxy benzaldehyde (**359**), phenyl acetic acid (**360**), phenyllactic acid (**361**), indole-3-carboxylic acid (**362**), indole-3-acetic acid (**363**), cyclo-(proline-methionine) (**364**), and cyclo-(phenylalanine-proline) (**365**).	[27]
Drechslera sp.	VPA, 500 µM	Increased production of benzophenone (**366**).	[40]
Macrophomina phaseolina	SVP, 1 mM	Induced production of 3-acetyl-2-methyl dihydro-furan-2(3*H*)-one (**367**) and 2-methyl-3-methylthio-butanoic acid (**368**).	[124]
Penicillium chrysogenum HLS111	SVP, 10 µM	Induced production of three new heterdimeric tetrahydroxanthone–chromanone lactones chrysoxanthones A–C (**369–371**).	[125]

Table S5. The examples of NaBut affecting production of fungal secondary metabolites.

Fungal Species	Concentration of NaBut	Compounds Induced/Enhanced/Decreased	Ref.
Aspergillus clavatus	9 µM	Enhanced production of cytochalasin E (**38**), patulin (**39**), and pseurotin A (**40**).	[25]
Cochliobolus lunatus TA26-46	10 mM	Induced production of novel brominated resorcylic acid lactones: 5-bromozeaenol (**372**) and 3,5-dibromozeaenol (**373**), together with four known analogures aigialomycin B (**374**), zeaenol (**375**), LL-Z1640-1 (**376**), and LL-Z1640-2 (**377**).	[127]
Diaporthe sp. PF20	100 µM	Enhanced production of piperine (**358**).	[123]
Leucostoma persoonii	100 µM	Induced production of cytosporone R (**380**). Enhanced production of cytosporones B (**346**), C (**378**) and E (**379**).	[128]
Penicillium brevicompactum	10 mM	Enhanced production of anthranilic acid (**381**) and ergosterol peroxide (**382**).	[129]
Phomopsis sp. XP-8	1 mM	Decreased production of pinoresinol (**383**), pinoresinol monoglucoside (**384**), pinoresinol diglucoside (**385**).	[130]
Phomopsis asparagi DHS-48	50 µM	Induced production of new compounds phaseolorin J (**386**) and phomoparagin D (**387**), along with known chromones phaseolorin D (**388**), chaetochromone B (**389**), and pleosporalin D (**390**), and cytochalasins J (**391**), J1 (**392**), J2 (**393**), J3 (**394**), H (**395**) and phomopchalasin D (**396**).	[131]
Trichoderma harzianum XS-20090075	10 µM	Induced production of three new terpenoids harzianolic acid (**397**), harzianone E (**398**) and 3,7,11-trihydroxy-cycloneran (**399**), together with 11 known sesquiterpenoids methyl 3,7-dihydroxy-15-cycloneranate (**400**), catenioblin C (**401**), ascotrichic acid (**402**), cyclonerotriol (**403**), (10*E*)-12-acetoxy-10-cycloneren-3,7-diol (**404**), cyclonerodiol (**405**), cyclonerodiol oxide (**406**) and epicyclonerodiol oxide (**407**), ophioceric acid (**408**), *ent*-trichoacorenol (**409**) and trichoacorenol (**410**).	[132]

Table S6. The examples of nicotinamide affecting production of fungal secondary metabolites.

Fungal Species	Concentration of nicotinamide	Compounds Induced/Enhanced/Decreased	Ref.
Chaetomium cancroideum	50 μM	Enhanced production of five polyketides included chaetophenols B (**293**) and C (**294**), chaetophenol G (**411**), and cancrolides A (**412**) and B (**413**).	[134]
C. mollipilium	100 μM	Induced production of new polyketides mollipilin A–E (**414–418**), along with two known compounds mollipilin F (**419**) and aureonitol (**420**).	[135]
Eupenicillium sp. LG41	1.5 mg/L	Induced production of two decalin-containing compounds eupenicinicols C (**421**) and D (**422**) along with their biosynthetic precursors, eujavanicol A (**423**) and eupenicinicol A (**424**).	[136]
Fusarium graminearum	500 μg/mL	Decreased production of ergosterol peroxide (**382**) and deoxynivalenol (**425**).	[137]
Graphiopsis chlorocephala	10 μM	Induced production of benzophenones cephalanones A–F (**426–431**) and 2-(2,6-dihydroxy-4-methylbenzoyl)-6-hydroxybenzoic acid (**432**).	[138]
Penicillium brasilianum	100 μM	Decreased production of brasiliamide A (**261**), verruculogen TR2 (**262**), and penicillic acid (**162**).	[27]
P. brevicompactum	100 μM	Induced production of phenolic metabolites: *p*-hydroxybenzaldehyde (**359**), phenyl acetic acid (**360**), *p*-anisic acid (**433**), *p*-anisic acid methyl ester (**434**), benzyl anisate (**435**), syringic acid (**436**), sinapic acid (**437**), acetosyringone (**438**), and gentisaldehyde (**439**).	[129]

Table S7. The examples of other histone deacetylase modifiers affecting production of fungal secondary metabolites.

Modifier and Its Concentration	Fungal Species	Compounds Induced/Enhanced/Decreased	Ref.
Dihydrocoumarin, 5 mM	*Monascus ruber* M7	Increased production of monasfluol B (**452**), acetyl-monasfluol B (**453**) and azaphilone C (**454**). Decreased production of citrinin (**455**).	[45]
Entinostat, 500 µM	*Hypomyces* sp. CLG4	Induced production of (3*R*)-6-hydroxymellein (**154**), (3*R*)-6-methoxymellein (**157**), 6-demethylkigelin (**456**), (3*R*)-6,7-dimethoxymellein (**457**), pyrolin (**458**), and terrain (**459**).	[141]
2-Hexyl-4-pentynoic acid, 37.5 mg/L	*Aspergillus versicolor*	Increased production of diorcinol (**53**), curvularin (**460**), and cyclo-(L-Trp-L-Phe) (**461**).	[48]
Octanoylhydroxamic acid, 500 µM	*Drechslera* sp.	Increased production of benzophenone (**366**).	[40]
Quercetin, 100 µM	*Aspergillus amstelodami* VR177L	Induced production of vinblastine (**462**).	[142]
Quercetin, 100 µM	*Penicillium concavoradulozum* VE89L	Induced production of vinblastine (**462**).	[142]

Table S8. The examples of two types of chemical epigenetic modifiers affecting production of fungal secondary metabolites.

Fungal Species	Modifier ant Its Concentration	Compounds Induced/Enhanced/Decreased	Ref.
Alternaria sp.	5-Aza (250 µM) + SAHA (500 µM)	Induced production of alternariol (**26**), alternariol-5-*O*-methyl ether (**27**), 3′-hydroxyalternariol-5-*O*-methyl ether (**28**), altenusin (**29**), tenuazonic acid (**30**), and altertoxin II (**31**).	[51]
Aspergillus sp. SCSIOW2	5-Aza (1 mM) + SBHA (1 mM)	Induced production of three new eremophilane-type sesquiterpenes, dihydrobipolaroxin B (**487**), dihydrobipolaroxin C (**488**), and dihydrobipolaroxin D (**489**), along with one known analogue, dihydrobipolaroxin (**490**).	[146]
Aspergillus sp. SCSIOW3	5-Aza (1 mM) + SBHA (1 mM)	Induced production of one new diphenylether-*O*-glycoside namely diorcinol 3-*O*-α-D-ribofuranoside (**491**), along with seven known compounds (7*S*)-sydonic acid (**33**), (*S*)-sydowic acid (**37**), diorcinal (**492**), 3,3′-dihydroxy-5,5′-dimethyldibenzofuran (**493**), cordyol (**494**), gibellulin B (**495**), and cyclo-(L-Trp-L-Phe) (**496**).	[147]
Aspergillus clavatus	GlcNAc (0.5 µM) + NaBut (9 µM)	Increased production of pseurotin A (**40**).	[25]
Aspergillus fumigatus	5-Aza (500 µM) + SBHA (500 µM)	Induced production of ten alkaloids: 3-dehydroxymethylbisdethio-3,10a-bis(methylthio) gliotoxin (**497**), bisdethiobis (methylthio) gliotoxin (**498**), fumitremorgin C (**499**), fumiquinazoline J (**500**), pyripyropene A (**501**), chaetominine (**502**), fumigaclavine A (**503**), 9-deacetoxyfumigaclavine C (**504**), fumigaclavine C (**505**), and 3-hydroxyacetyl indole (**506**); six polyketides: helvolinic acid (**507**), rhizoctonic acid (**508**), monomethylsulochrin (**509**), 3-hydroxy-1,6-dimethoxy-8-methyl 9*H*-xanthen-9-one (**510**), questin (**511**) and 6-hydroxy-8-methoxy-3-methylisocoumarin (**512**); and three benzene derivatives: circinoporic acid (**513**), koaburaside (**514**), and methyl shikimate (**515**).	[148]
Aspergillus versicolor	5-Aza (100 µM) + SAHA (100 µM)	Induced production of two new nucleoside derivatives, kipukasins K (**516**) and L (**517**), and one new bisabolane sesquiterpene, aspergillusene E (**518**), along with four known nucleoside derivatives, kipukasin I (**519**), kipukasin H (**520**), kipukasin D (**521**), and kipukasin E (**522**), and ten known bisabolane sesquiterpenes, (7*R*)-hydroxysydonic acid (**32**), (7*S*)-sydonic acid (**33**), (*E*)-5-(hydroxymethyl)-2-(6′-methylhept-2′-en-2′-yl)phenol (**523**), (*Z*)-5-(hydroxymethyl)-2-(6′-methylhept-2′-en-2′-yl)phenol (**524**), 7-deoxy-7,14-didehydrosydonol (**525**), (7*R*)-sydonol (**526**), (7*R*)-methoxysydonol (**527**), (7S)-sydonol (**47**), (7S)-methoxysydonol (**528**), and aspergiterpenoid A (**529**).	[149]
Eutypella sp.	5-Aza (1 mM) + SBHA (1 mM)	Induced production of 21 sesquiterpenes including 17 undescribed eutypeterpenes A-Q (**530–546**), four known sesquiterpenes: xylariterpenoids A (**547**) and B (**548**),	[150]

		eudesma-3-en-11,15-diol (**549**), and eudesma-4-en-11,15-diol (**550**).	
Gibellula formosana	SBHA (1 mM) + RG-108 (1 mM)	Induced production of two new highly oxidized ergosterols, formosterols A (**551**) and B (**552**), and five new isariotin analogs, 12′-*O*-acetylisariotin A (**553**), 1-*epi*-isariotin A (**554**), and isariotins K–M (**555–557**), together with six known compounds isariotin A (**341**), formosterol C (**558**), isariotin C (**559**), isariotin E (**560**), TK-57-164A (**561**), and beauvericin (**562**).	[38]
Isaria tenuipes	SBHA (500 µM) + RG–108 (500 µM)	Induced production of a novel polyketide tenuipyrone (**563**) along with two plausible precusors cephalosporolides B (**564**) and F (**565**) of tenuipyrone (**563**).	[151]
Leucostoma persoonii	5-Aza (50 µM) + NaBut (100 µM)	Induced production of cytosporones B (**346**), C (**378**), E (**379**) and R (**380**).	[128]
Muscodor yucatanensis Ni30	5-Aza (50 µM) + SAHA (50 µM)	Enhanced production of ergosterol (**104**) and xylaguaianol C (**105**).	[69]
Pestalotiopsis acaciae	5-Aza (500 µM) + SAHA (500 µM)	Induced production of three novel aromatic compounds: 20-hydroxy-6′-hydroxymethyl-4′-methylphenyl-2,6-dihydroxy-3-(2-isopentenyl)benzoate (**566**), 4,6-dihydroxy-7-hydroxymethyl-3-methylcoumarin (**567**) and 4,6-dihydroxy-3,7-dimethylcoumarin (**568**).	[152]
Ustilago maydis	5-Aza (500 µM) + SBHA (500 µM)	Induced production of ustilagic acids B (**569**) and C (**570**).	[153]
Xylaria psidii	5-Aza (5 µM) + SAHA (10 µM)	Increased production of resveratrol (**132**).	[75]
